# Improved wideband electromagnetic performance in La^3+^ substituted Mn–Zn spinel ferrites: structure–microstructure regulation of grain-boundary non-Debye relaxation

**DOI:** 10.1039/d6ra09155e

**Published:** 2026-11-05

**Authors:** Sabbir Hasan Munna, Kazi Afsana Rahman, Md. Alamgir Hossain

**Affiliations:** a Solid State Physics Laboratory, Dept. of Physics, Khulna University of Engineering & Technology Khulna-9203 Bangladesh liton@phy.kuet.ac.bd

## Abstract

Grain-boundary related electrical relaxation and microstructural evolution are important factors in the frequency-dependent response of Mn–Zn ferrite. Here, La^3+^ substituted Mn–Zn ferrites, were synthesized by a solid-state route (calcination at 800 °C for 4 h and sintering at 1200 °C for 6 h). Rietveld refinement confirms a spinel-dominant cubic phase for all compositions. A minor orthorhombic phase appears above a La^3+^ threshold and remains below about 4%. The lattice parameter shows only small variation (about 8.41–8.45 Å). SEM and density analysis show grain refinement from about 1.87 µm to 1.07 µm and a non-monotonic porosity evolution (about 16–36%). Room-temperature hysteresis loops confirm soft magnetic behavior. The saturation magnetization increases strongly and reaches 33.96 emu g^−1^ at an intermediate La^3+^ level. Wideband measurements show composition-dependent relatively stable real permeability and strongly reduced magnetic loss at high frequency. Dielectric and impedance spectra show strong low-frequency dispersion consistent with Maxwell–Wagner interfacial polarization. Electric modulus and Cole–Cole plots exhibit depressed arcs, indicating non-Debye relaxation with distributed relaxation times dominated by grain boundaries. Nyquist fitting separates grain and grain-boundary responses and shows composition-tunable interfacial barrier behavior. Overall, the results demonstrate that La^3+^ substitution modifies the structural and microstructural state of Mn–Zn ferrites, establishing a composition-dependent balance between magnetic properties, interfacial relaxation, and high-frequency electromagnetic response.

## Abstract 2

Grain-boundary related electrical relaxation and microstructural evolution are important factors in the frequency-dependent response of Mn–Zn ferrite.

## Graphical abstract

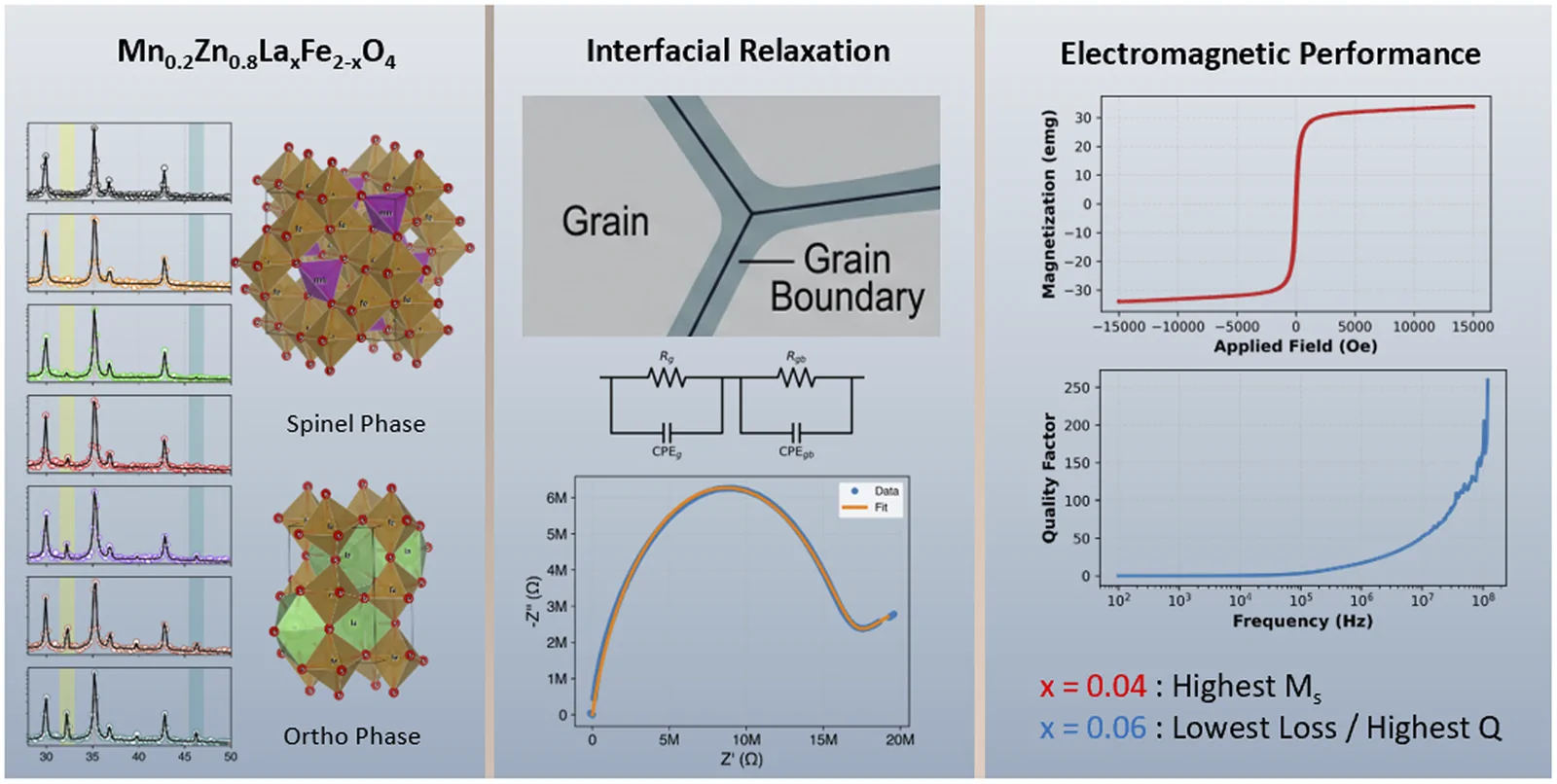

## 1 Introduction

Doped Mn–Zn spinel ferrites are widely studied because they combine soft ferrimagnetism with relatively high electrical resistivity.^1–3^ This combination is important for modern transformers, inductor cores, and for impedance-based sensing.^4–6^ In recent years, switching electronics has moved toward higher frequencies to reduce component size and increase power density.^7–9^ This shift increases the need for ferrite ceramics that keep useful permeability while limiting magnetic loss in the MHz region.^8,10^ At the same time, it is now accepted that chemistry alone does not control performance. Processing-driven microstructure, especially grain size, porosity and grain-boundary chemistry, can strongly control both magnetization dynamics and electrical relaxation under alternating fields.^11–13^ Beyond conventional soft-magnetic ferrites, alternative material architectures have also been investigated for electromagnetic applications. For example, MXene-based magnetic nanohybrids have demonstrated electromagnetic interference (EMI) shielding and strain-sensing capabilities, while graphene–reinforced polymer composites have been explored for mitigating electromagnetic radiation pollution.^14,15^ These distinct material platforms highlight the broader interest in developing materials with tailored electromagnetic performance.

Many previous studies have mainly focused on the crystal structure and static magnetic properties of doped Mn–Zn ferrites using techniques such as XRD, SEM or TEM, and VSM, which clearly show that densification, grain growth, and pore evolution play important roles rather than being minor details.^16–24^ They influence saturation magnetization, coercivity, and domain-wall mobility through pinning and demagnetizing fields.^25–28^ A common trend is that improved densification and well-developed grains often support better soft magnetic response, while porous or fine-grained microstructures can increase loss through internal stresses and reduced magnetic connectivity.^29^ However, these works usually do not extend to a continuous wideband response. As a result, they do not directly show how the same microstructural changes control permeability stability and loss suppression when frequency increases into the MHz range.

Electrical spectroscopy is included in some reports, but the frequency windows and analysis depth are not consistent.^30,31^ Low-frequency impedance studies, typically up to a few MHz, support the standard grain and grain-boundary barrier picture for ferrites.^32,33^ In this model, grains are relatively more conductive, and grain boundaries are more resistive.^11^ This contrast produces Maxwell–Wagner interfacial polarization and strong low-frequency dielectric dispersion, often explained with Koop's two-layer concept.^34–36^ Because grain boundaries are chemically and structurally non-uniform, relaxation is not purely Debye-like.^37^ It often appears as depressed arcs in Cole–Cole or Nyquist plots.^38^ Yet, in many cases, the grain and grain-boundary separation remain qualitative. Detailed equivalent-circuit fitting using constant phase elements is often not fully reported, and CPE exponents needed to quantify non-Debye dispersion are frequently missing.^39^ This limits strict comparison across dopants and across composition series.

Other studies have focused on much higher frequency responses in the microwave band, where GHz-range impedance and dielectric measurements can highlight the limits of fast polarization mechanisms while slow interfacial effects become less dominant.^18,40,41^ These works are valuable for understanding intrinsic high-frequency behavior. However, they do not replace continuous characterization across the power-electronics window. For many devices, the most critical region lies well below the microwave band, especially between about 1 MHz and 120 MHz. In this region, magnetic domain processes overlap with grain-boundary-controlled electrical barriers, and the response still depends strongly on microstructure and defects. When one set of studies covers only up to a few MHz and another set covers mainly GHz behavior, the transition from interfacial low-frequency relaxation to intrinsic high-frequency response remains weakly mapped.

La^3+^ substitution adds further uncertainty because published magnetic trends are not fully consistent.^42–45^ Some La^3+^ substituted Mn–Zn ferrite studies report a peak in saturation magnetization at an intermediate La^3+^ level, while others report a monotonic decrease with La^3+^ addition. This disagreement is physically plausible because La^3+^ is large and non-magnetic, and its effect depends on solubility, defect compensation, and processing.^46^ If La^3+^ segregates at grain boundaries, it can inhibit grain growth and alter densification, which changes domain-wall mobility and loss.^47^ If it enters the lattice and modifies Fe^2+^/Fe^3+^ balance or cation distribution between A and B sites, it can change the net sublattice moment and can also increase spin canting, reducing the collinear magnetization component.^48,49^ Limited solubility can also lead to minor secondary-phase formation at higher substitution.^50^ These linked effects mean that La^3+^ trends cannot be judged from magnetization alone. They require a combined phase, microstructure, and broadband relaxation interpretation.

Although La^3+^ substituted Mn–Zn ferrites have been widely investigated, prior research typically evaluates their static structural, magnetic, or dielectric properties in isolation. Systematic studies quantitatively linking microstructural evolution to wideband electrical relaxation and high-frequency magnetic performance remain limited. Specifically, the exact relationship between La^3+^ induced secondary-phase formation, grain refinement, and the distribution of interfacial relaxation processes is not fully understood. To advance beyond standard static characterization, this work mathematically isolates the dynamic interfacial response across a wideband frequency range (100 Hz to 120 MHz). Using quantitative Nyquist and Cole–Cole equivalent-circuit modeling, we separate the intrinsic grain response from the grain-boundary barrier network. By correlating these fitted parameters directly with microstructural evolution, we demonstrate that La^3+^ actively redistributes grain-boundary barrier resistance. This redistribution shifts the non-Debye interfacial relaxation window, directly suppressing MHz-range magnetic loss and defining the balance between permeability, loss tangent, and high-frequency quality factor.

## 2 Materials and methods

### 2.1 Materials and composition design

Polycrystalline La^3+^-substituted Mn–Zn ferrites with nominal composition Mn_0.20_Zn_0.80_La_*x*_Fe_2−*x*_O_4_ (*x* = 0.00, 0.01, 0.02, 0.03, 0.04, 0.06, 0.08) (MZLF) were prepared by a conventional solid-state reaction (SSR) route as shown in Fig. 1.^51^ For each composition, 20 g batches were formulated from MnO_2_, ZnO, La_2_O_3_ and Fe_2_O_3_ powders weighed in stoichiometric proportions. Manganese dioxide (LR technical grade, 70% assay; Loba Chemie, product code 4504D) was used as the Mn source. According to the supplier specification, this powder contains 70% MnO_2_ with maximum impurity levels of 0.05% chloride, 0.10% sulphate, 0.05% iron, 0.50% water-insoluble matter, and 0.005% total heavy metals (as Pb). Zinc oxide (special grade, 99.0%), lanthanum oxide (GPR, 99.0%; BDH Chemicals), and iron(iii) oxide (99.0%; Giant Chem Solutions) were used as received. The same powder batches were used across the full composition series.

**Fig. 1:**
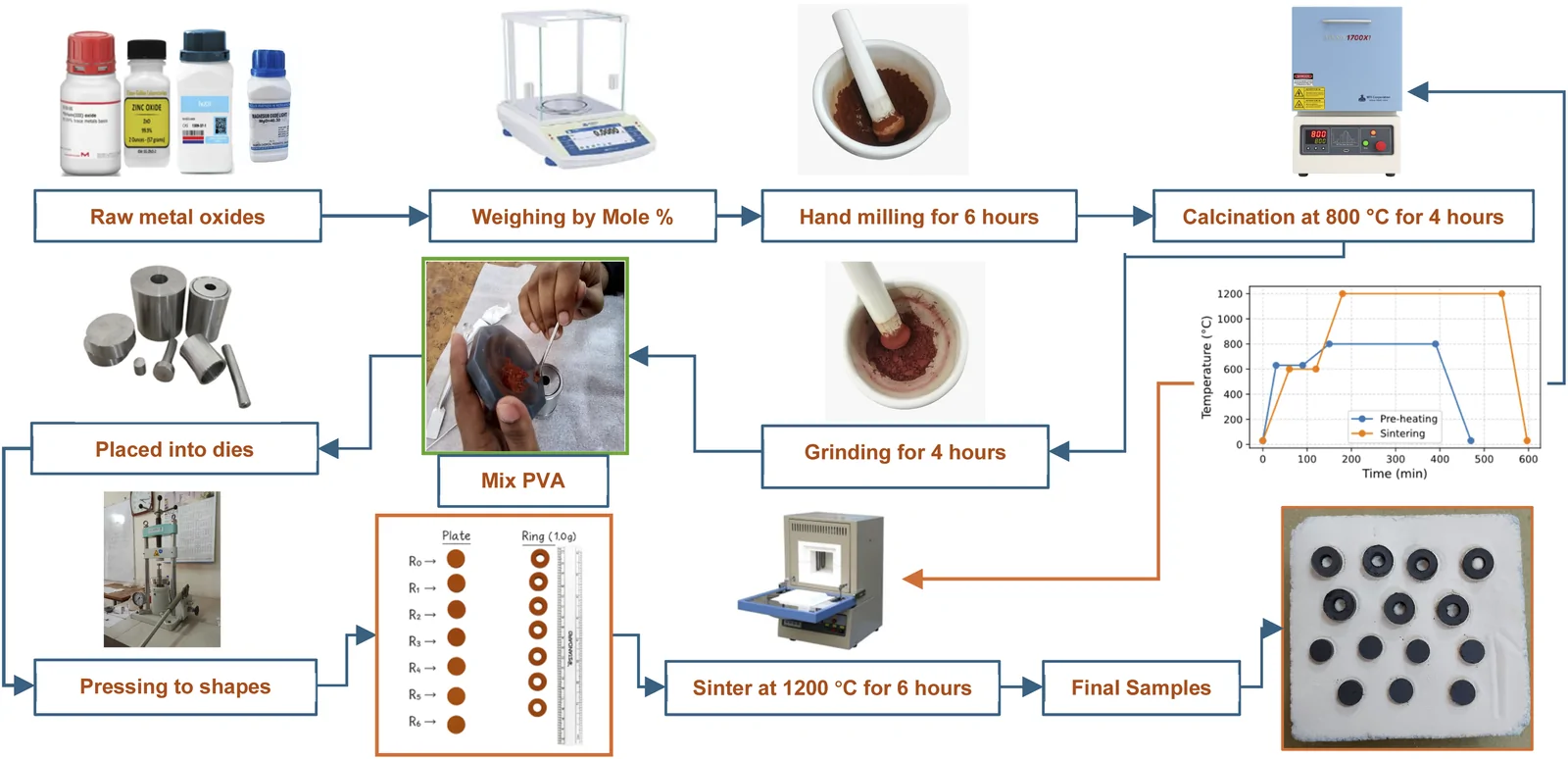
Schematic flowchart outlining the solid-state reaction synthesis process for the La-substituted Mn–Zn ferrite samples.

Stoichiometric powder mixtures were hand-milled in an agate mortar for 6 h to promote homogenization. The mixed powders were placed in covered alumina crucibles and calcined in static air using the following schedule: ambient (≈30 °C) to 630 °C in 60 min, dwell for 60 min; ramp to 800 °C in 60 min, dwell for 240 min; followed by natural furnace cooling (cooling rate ≈ 10 °C min^−1^ in the 800–200 °C interval). The calcined cake was re-ground in an agate mortar for approximately 4 h to obtain a fine powder.

An aqueous poly(vinyl alcohol) (PVA) binder solution (5 wt%) was added dropwise to produce a pressable granulate (PVA content < 3 wt% relative to oxide solids). The intermediate thermal dwell at 600 °C employed in both thermal profiles (calcination and sintering) was selected to ensure complete binder burnout before the main densification stage. Because the PVA binder acts as an electromagnetically transparent matrix with negligible high-frequency response, the measured complex permittivity and permeability of the pressed samples are directly attributed to the intrinsic electromagnetic behavior of the solid ferrite active material.^52^

Discs (diameter 8.40–8.98 mm; thickness 1.80–2.28 mm) and rings (outer diameter 10.82–11.41 mm; inner diameter 4.89–5.24 mm; thickness 2.29–3.11 mm) were uniaxially pressed in hardened steel dies at 20 kN with a 20 s dwell (applied stress ≈ 315–360 MPa for discs). Green density was not measured separately. Instead, identical powder conditioning, binder addition, die set, press load, and dwell time were maintained for all compositions to keep the green state as comparable as possible.

Green compacts were placed on alumina setters and sintered in static air using the schedule: ambient to 600 °C in 60 min, dwell for 60 min; ramp to 1200 °C in 60 min, dwell for 360 min; followed by natural furnace cooling (approximately 10 °C min^−1^ during the main cooling span). Minor surface irregularities were removed by light grinding where required. Masses were measured using an analytical balance (0.1 mg resolution). Bulk density was determined from measured mass and geometric volume, and open/closed porosity was estimated by comparing bulk density with X-ray density calculated from refined lattice parameters.

### 2.2 Experimental methodology

Phase constitution was examined by powder X-ray diffraction (Bruker D2 Phaser; *θ*–2*θ* geometry; goniometer radius 141 mm) using Cu Kα radiation (*λ*_1_ = 1.5406 Å, *λ*_2_ = 1.54439 Å) without a monochromator or analyzer. Finely ground powder for each sample was lightly pressed and back-loaded into a flat specimen holder to reduce preferred orientation. Data were collected over 2*θ* = 10.00–79.82° with a step size of 0.2006° using the instrument's fast acquisition mode. Fixed divergence and anti-scatter slits were 10.5 mm.

Rietveld refinements were performed using the FullProf suite.^53^ Initial structural models were identified using “Match!” software and reference patterns from the Crystallographic Open Database (COD).^54^ Refinable parameters included scale factor, background, peak-shape terms, lattice parameters, and selected profile and structural terms as required to achieve stable convergence. Refinement quality was assessed using *R*_p_, *R*_wp_, and *χ*^2^. Where applicable, phase fractions were obtained from refined scale factors with unit-cell volume correction.

Microstructure and morphology were investigated by scanning electron microscopy (JEOL JCM-7000 NeoScope, secondary electron imaging) at an accelerating voltage of 15 kV, working distance ≈ 12.4 mm, and high-vacuum mode. Polished pellet surfaces were sputter-coated with a thin Ag layer to reduce charging and mounted on Al stubs using carbon adhesive. Representative fields (at least five randomly selected areas per sample) were imaged at 2500–10 000× (field of view ≈ 25.6 µm × 19.2 µm). Grain size distributions were obtained from secondary electron micrographs by image segmentation in ImageJ.^55^ To minimize observer bias across the varying polished and fractured surface topographies, a semi-automatic pipeline was utilized with locked downstream algorithmic controls, including Gaussian pre-filtering for noise suppression and morphological size-filtering to reject artifacts. From these controlled masks, particle/grain size was extracted using Feret diameter statistics across a statistically robust sampling pool (*N* > 100 grains per composition). To further isolate the true grain scale from segmentation extremes, the resulting datasets were mathematically modeled using log-normal probability distribution functions. Full details of the image-processing protocols are provided in SI Section ST3.

Energy-dispersive X-ray spectroscopy (EDXS) was performed in area-analysis mode at 15 kV and a working distance ≈ 12.5 mm using the instrument SDD detector. Typical conditions were probe current high-PC, process time *T*_2_, and live time 30 s (real time 30.9 s; dead time ≈ 2%), with count rates on the order of 10^3^ counts per s. EDXS was used to confirm elemental presence and approximate compositional homogeneity, recognizing that quantification of light elements and matrix effects can introduce systematic uncertainties.

Room-temperature magnetic hysteresis loops were recorded using a Lake Shore 8400 vibrating sample magnetometer in continuous acquisition mode. Fields up to ±15 kOe were applied using a programmed field step of 100 Oe. Moment calibration was performed using a 5000 Oe reference field with a sensitivity of −2017.49 emu V^−1^. Approximately 30 ± 2 mg of each sample (pellet fragments) was used, and mass magnetization was calculated using the measured mass. No demagnetization correction was applied (demagnetization factor set to 0) to maintain consistent comparative conditions across the composition series. Coercivity and remanent magnetization were extracted using the instrument's automated routine. Where required, post-processing smoothing was carried out using a Savitzky–Golay filter.^56^

Electrical response was measured by impedance spectroscopy using a Wayne Kerr 6500B precision impedance analyzer over 100 Hz to 120 MHz at 300 K. Both pellet faces (diameter ≈ 8.4–9.0 mm) were fully sputtered with Au to form electrodes, and the electrode area was defined by the pellet diameter. An excitation amplitude of 100 mV rms was used. Open and short compensation was performed before data acquisition. Complex impedance and permittivity were analyzed using equivalent-circuit fitting of Nyquist plots to separate grain and grain-boundary contributions. Additional analysis was performed using dielectric modulus formalisms and Cole–Cole representations to assess relaxation behavior.

Data handling (XRD formatting and refinement summaries, SEM image analysis, magnetic loop processing, impedance fitting, and figure preparation) was performed using Python-based scripts. The computational environment specification and analysis scripts can be provided as supplementary material to support reproducibility.

## 3 Results and discussion

### 3.1 Phase formation and structural evolution

This section discusses how La^3+^ substitution affects phase stability and crystal structure in the MZLF series. XRD patterns and Rietveld refinement are used to identify the dominant spinel phase and any secondary phases. Changes in peak position, lattice parameter, and phase fraction are then linked to solubility limits and dopant-induced lattice distortion.

#### 3.1.1 Phase identification and crystallographic models

Powder XRD patterns (Fig. 2a) confirm that the MZLF series is dominated by the cubic spinel phase (*Fd*3̄*m*) across the full substitution range, the 3D crystal structure of which is schematically depicted in SI Fig. S1. Rietveld refinement data (SI Table S1) show a single-phase spinel response for *x* = 0.00, 0.01 (*Fd*3̄*m* = 100%). A minor orthorhombic secondary phase (*Pnma*, Fig. 2b) becomes detectable at *x* = 0.02 and persists up to *x* = 0.08. While the refined fractions for *x* = 0.02 (1.01%) and *x* = 0.03 (1.07%) are at the standard quantification limit of laboratory XRD and must be treated cautiously as trace amounts, the secondary phase grows systematically at higher substitution levels, reaching 1.73% (*x* = 0.04) and 4.06% (*x* = 0.06) before slightly decreasing to 3.81% (*x* = 0.08). The secondary-phase onset suggests a solubility limit for La^3+^ (and, more generally, rare-earth RE^3+^) substitution in the spinel lattice under the present processing conditions, as commonly reported for RE-doped ferrites where excess dopant segregates and/or forms a minor secondary phase beyond a threshold composition.^45,57^ The complete set of individual Rietveld refinement profiles, including residual difference curves for all compositions, is provided in Fig. S2.

**Fig. 2:**
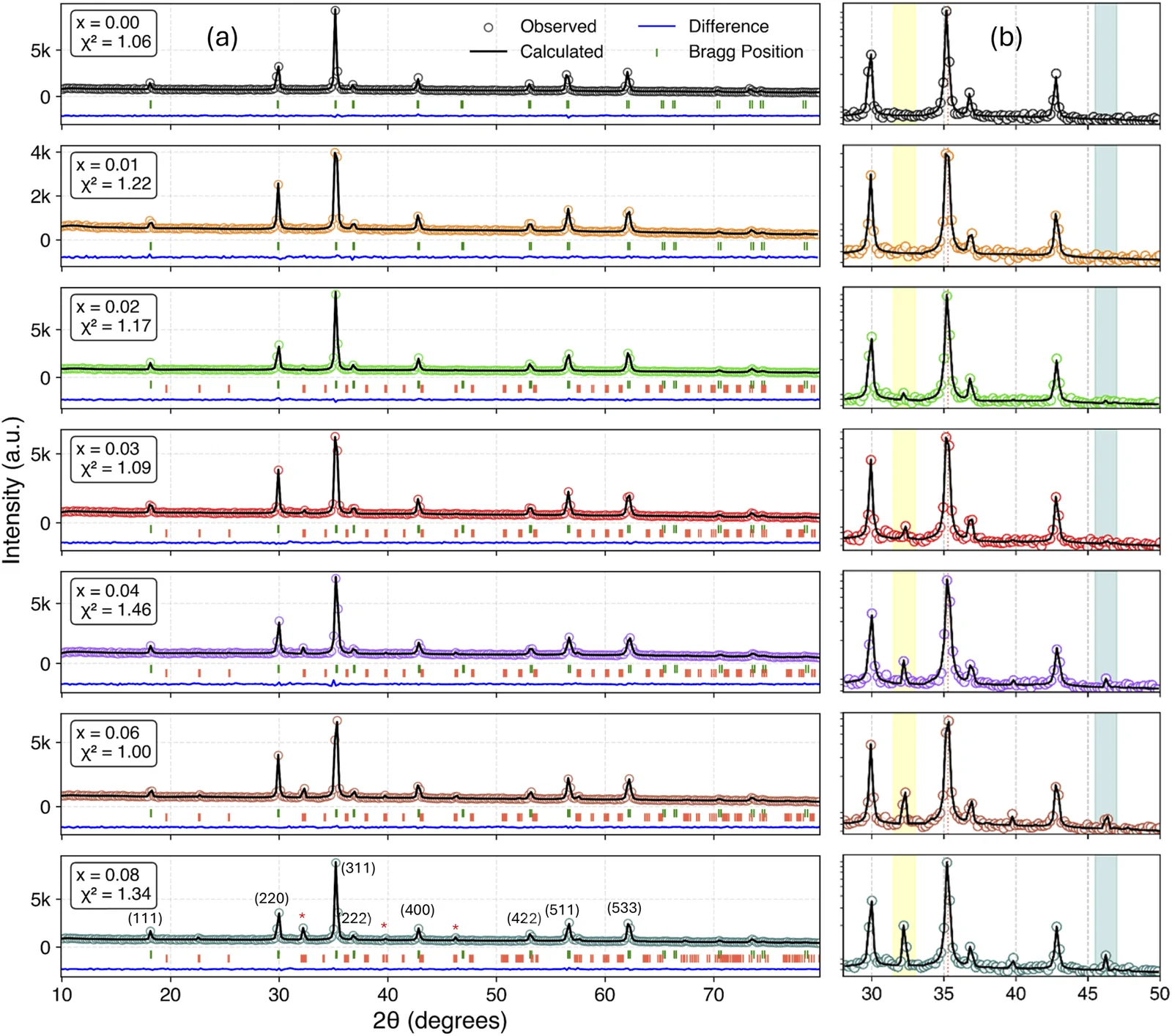
Rietveld refined XRD profiles of MZLF composites showing (a) the full pattern refinements from 10° to 80° and (b) a magnified view highlighting the evolution of secondary *Pnma* phase reflections.

Refinement quality (*R*_p_, *R*_wp_, and *χ*^2^) remains acceptable across the series, supporting the reliability of the extracted phase fractions. The refined unit-cell volume shows a small, non-monotonic variation (*e.g.*, 605.19 Å^3^ at *x* = 0.00 and 600.47 Å^3^ at *x* = 0.04), consistent with competing effects of La^3+^-induced lattice strain, charge-compensation defects, and cation redistribution, together with partial La^3+^ partitioning into the minor *Pnma* phase. The slight reduction of the *Pnma* fraction at *x* = 0.08 compared with *x* = 0.06 falls within the normal uncertainty of low-level phase quantification caused by peak overlap and scale-factor stability. The clear appearance of *Pnma* begins at *x* = 0.02 and a minor fraction remains at higher La^3+^ content.

The exact lattice parameter *a*_0_ of the cubic spinel was also estimated using the Nelson–Riley extrapolation method.^58^ The Nelson–Riley lattice-parameter trend is summarized in Fig. 3a, while representative linear fitting plots are shown in Fig. S3, and the corresponding fitting parameters and statistics are listed in Table S2. It is noted that for certain compositions (*e.g.*, *x* = 0.03 and 0.04), the linear regression yields very low *R*^2^ values. As established in recent literature, this reflects a near-zero Nelson–Riley slope rather than excessive scatter, indicating that the underlying lattice parameter carries little residual systematic error to extrapolate away.^59^ Because the regression slope approaches zero, minor random scatter from peak-fitting dominates the variance, inherently driving *R*^2^ downward. Therefore, while the Nelson–Riley intercept provides a useful comparative estimation of *a*_0_, the full-pattern Rietveld refinement remains the primary and most robust indicator of structural evolution. The extracted *a*_0_ values vary only slightly within 8.408–8.445 Å and show a non-monotonic response with *x*. The undoped sample gives *a*_0_ = 8.445 Å. After low La^3+^ addition, *a*_0_ decreases to 8.408 Å at *x* = 0.01. For *x* ≥ 0.02, *a*_0_ recovers and remains near 8.42–8.44 Å (for example, 8.436 Å at *x* = 0.02 and 8.436 Å at *x* = 0.08). The observed non-Vegard behavior may arise from ionic size mismatch, defect-mediated charge compensation, and cation redistribution between A and B sites.^60^ It is also consistent with partial La^3+^ partitioning into the minor *Pnma* phase at higher *x*, which reduces the amount of La^3+^ entering the spinel lattice. Although La^3+^ has a larger ionic radius than Fe^3+^, La^3+^ substitution does not always produce a monotonic lattice expansion in spinel ferrites. The radius mismatch can promote cation redistribution between A and B sites and generate internal stress and local lattice distortion, which can reduce the refined average lattice parameter and inhibit lattice growth, especially at low La^3+^ content.^61^

**Fig. 3:**
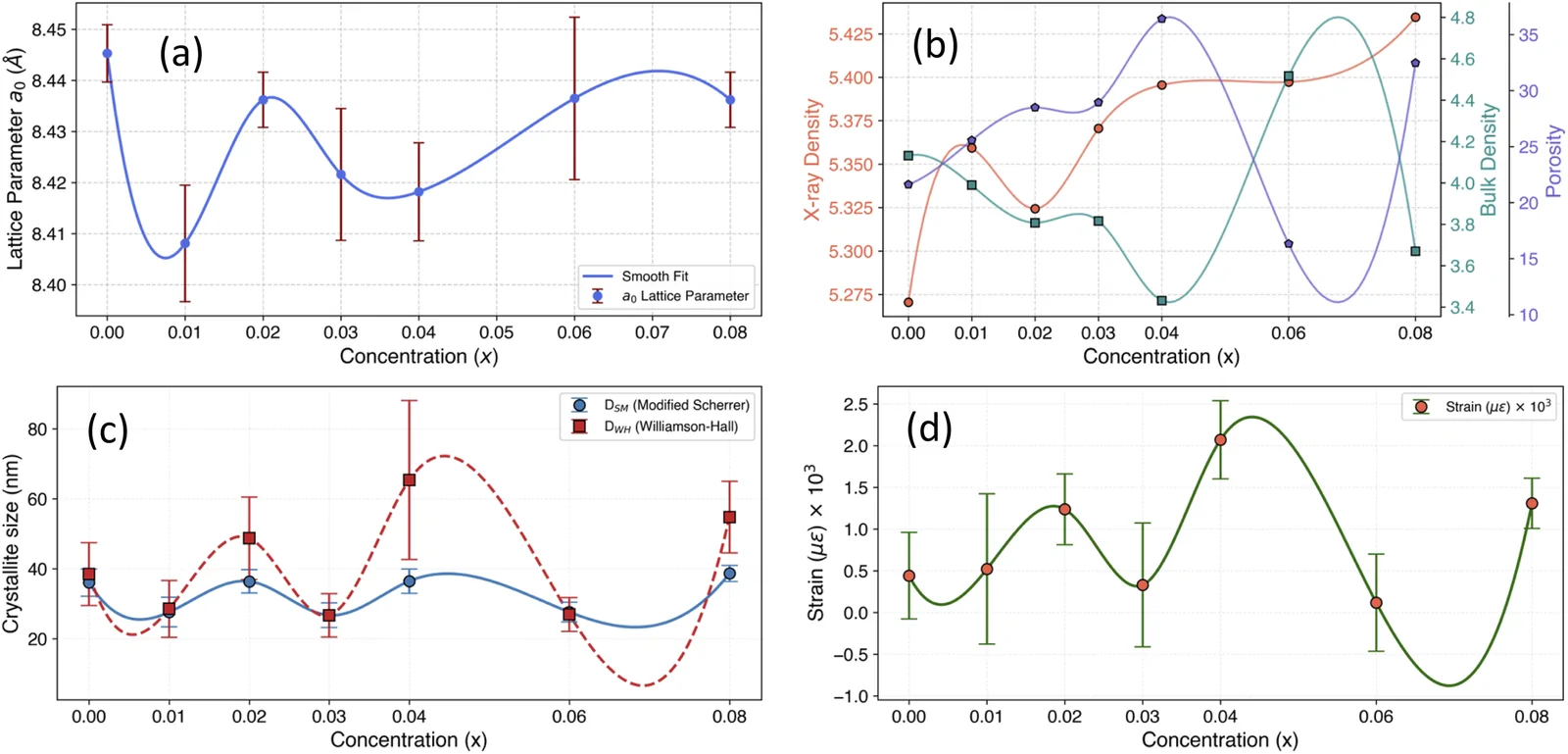
Compositional dependence of structural, physical, and microstructural parameters of MZLF samples as a function of lanthanum concentration: (a) variation of lattice parameter *a*_0_, (b) multi-axis plot showing the correlation between X-ray density, bulk density, and percentage porosity, (c) comparison of crystallite size estimated *via* modified Scherrer and Williamson–Hall methods, and (d) evolution of the internal lattice.

#### 3.1.2 Crystallite-scale microstructure from XRD line broadening

XRD line broadening was evaluated using the modified Scherrer (MS) and Williamson–Hall (WH) formalisms to separate domain-size and microstrain contributions.^62^ Crystallite sizes obtained from both methods are shown as a function of La^3+^ content in Fig. 3c. At *x* = 0.00 the values are *D*_MS_ = 36.05 nm and *D*_WH_ = 38.50 nm. These decrease to 27.63 nm (MS) and 28.54 nm (WH) at *x* = 0.01, showing smaller coherent domains at low La^3+^ substitution. At higher La^3+^ levels the WH sizes increase more strongly than the MS sizes, as seen at *x* = 0.04 where *D*_MS_ = 36.46 nm and *D*_WH_ = 65.34 nm, and at *x* = 0.08 where *D*_MS_ = 38.69 nm and *D*_WH_ = 54.72 nm. The larger WH sizes are consistent with additional strain broadening that is accounted for by the WH analysis but not by the MS method. The related linear regression plots are shown in Fig. S4.

The WH microstrain (*ε*) is strongly composition-dependent (Fig. 3d). Strain increases from 441*µε* at *x* = 0.00 to 1238*µε* at *x* = 0.02 and reaches 2071*µε* at *x* = 0.04, then drops sharply to 118*µε* at *x* = 0.06, indicating that defect-induced distortion does not scale linearly with *x*. This non-monotonicity is consistent with competing effects of La^3+^-driven lattice mismatch and charge compensation, together with partial La^3+^ partitioning into the minor secondary phase at higher *x* that may reduce effective distortion in the main spinel lattice. Because *ε* uncertainties are comparatively large for several compositions, strain is treated as a semi-quantitative indicator of defect-related broadening rather than an absolute value.

Defect descriptors derived from size-strain analysis further support La^3+^-induced disorder. The size-based proxy (*δ* = 1/*D*^2^) increases when crystallite size is smaller, as seen in the rise of *δ*_SM_ from 76.96 at *x* = 0.00 to 130.97 at *x* = 0.01. Strain-sensitive metrics are highest at the point of maximum *ε*, where *ρ*_WS_ = 18.39 and *ρ*_*ε*_ ≈ 1.20 (*x* = 0.04) against *ρ*_WS_ = 6.65 and *ρ*_*ε*_ ≈ 0.05 at *x* = 0.00.^63,64^ Although these values depend on model assumptions, their trends consistently indicate increased defect-related strain and dislocation density proxies in selected La^3+^ compositions, providing a structural basis for subsequent changes in magnetic and dielectric relaxation.

The refined lattice parameter was also used to calculate X-ray density (*ρ*_X_), which was compared with the measured bulk density (*ρ*_B_) to estimate porosity (Table 1). While *ρ*_X_ varies only modestly ≈5.27–5.43 g cm^−3^, *ρ*_B_ changes strongly with composition, confirming that La^3+^ mainly affects densification and pore evolution rather than unit-cell density. Porosity (*Φ*) increases from 21.61% at *x* = 0.00 to a maximum of 36.40% at *x* = 0.04 (*ρ*_B_ = 3.431 g cm^−3^), then decreases to 16.31% at *x* = 0.06 (*ρ*_B_ = 4.517 g cm^−3^) before rising again to 32.46% at *x* = 0.08. The non-monotonic densification trend is consistent with La^3+^-modified grain-boundary chemistry and sintering kinetics. This provides a microstructural basis for interpreting the composition-dependent permeability, dielectric dispersion, and grain-boundary resistance presented later.

**Table 1:** Summary of the structural parameters for the synthesized samples^a^

*x*	*a*_0_	*V*	*D*_SM_	*D*_WH_	*ρ*_XRD_	*ρ*_B_	*Φ*	*δ*_SM_	*δ*_WH_	*ρ*_WS_
0.00	8.4453	602.34	34.82	36.99	5.2705	4.1317	21.6065	76.96	67.46	6.65
0.01	8.4081	594.42	32.75	36.80	5.3594	3.9896	25.5585	130.97	122.75	10.61
0.02	8.4362	600.39	34.66	44.32	5.3244	3.8077	28.4854	75.46	42.12	14.73
0.03	8.4216	597.28	38.00	48.65	5.3706	3.8162	28.9418	139.82	139.90	7.16
0.04	8.4182	596.56	33.46	38.44	5.3956	3.4314	36.4039	75.22	23.42	18.39
0.06	8.4365	600.46	33.39	32.60	5.3973	4.5167	16.3146	130.85	137.41	2.53
0.08	8.4362	600.39	37.39	42.96	5.4346	3.6704	32.4624	66.82	33.39	13.88

a *x*: doping concentration (mol%); *a*_0_: lattice parameter (Å); *V*: unit cell volume (Å^3^); *D*_SM_ and *D*_WH_: crystallite size (nm); *ρ*_XRD_: theoretical density (g cm^−3^); *ρ*_B_: bulk density (g cm^−3^); *Φ*: porosity (%); *δ*_SM_, *δ*_WH_ and *ρ*_WS_: dislocation density (10^15^ lines per m^2^).

Spinel structure evaluation and phase stability were further assessed using geometric parameters derived from the cubic spinel lattice. The calculated A and B site hopping lengths (*L*_A_ and *L*_B_) quantify the effective cation-to-cation transport path lengths for electron hopping within the tetrahedral (A) and octahedral [B] sublattices, while the A–O and B–O bond lengths (*d*_AX_ and *d*_BX_) describe the local polyhedral geometry that controls exchange and hopping barriers. Across *x* = 0.00–0.08, *L*_A_ and *L*_B_ vary only slightly and show a small non-monotonic response, with a modest contraction at low La^3+^ content (*x* = 0.01) followed by recovery toward the undoped values at higher *x*, consistent with the small lattice-parameter changes and La^3+^-induced local strain compensation. The extended bond-distance set (Table S7) shows the same narrow variations, indicating that the cubic spinel framework remains geometrically stable even as a minor *Pnma* phase appears beyond *x* ≥ 0.02. The characteristic bond angles (*θ*_1_–*θ*_5_) remain essentially constant within the reported precision (Table S8). La^3+^ substitution therefore appears to act mainly on bond lengths and hopping distances, without producing a clear tilt or angular distortion of the average spinel network. Tables S6–S8 summarize these parameters, and Fig. S5 visualizes the composition-dependent variations in bond distances and bond angles to highlight the small but systematic geometric changes associated with La^3+^ substitution and the observed phase stability window.

### 3.2 Microstructure and morphology

#### 3.2.1 Qualitative morphology evolution

SEM micrographs reveal a clear composition-dependent change in fracture and polished-surface morphology. The undoped sample (*x* = 0.00) shows a flat polished grain matrix containing isolated pores. Addition of a small amount of La^3+^ (*x* = 0.01) produces dense micro-pitting on otherwise polished grains. At *x* = 0.02 the microstructure becomes more compact and fine-grained, with pores largely restricted to narrow intergranular junctions (Fig. 4a and b). More pronounced topography appears at intermediate La^3+^ contents. Deep crater-like voids form at *x* = 0.03, while a coral-like interlocking network with open channels develops at *x* = 0.04, consistent with incomplete densification and pore coalescence (Fig. 4d and e). This trend matches the porosity maximum derived from density measurements at *x* = 0.04. Densification recovers partially at higher substitution. At *x* = 0.06 the fracture surface is dense and block-like with few open channels, whereas at *x* = 0.08 it is shallower but strongly micro-textured, with voids present mainly as fine crevices (Fig. 4f and g).

**Fig. 4:**
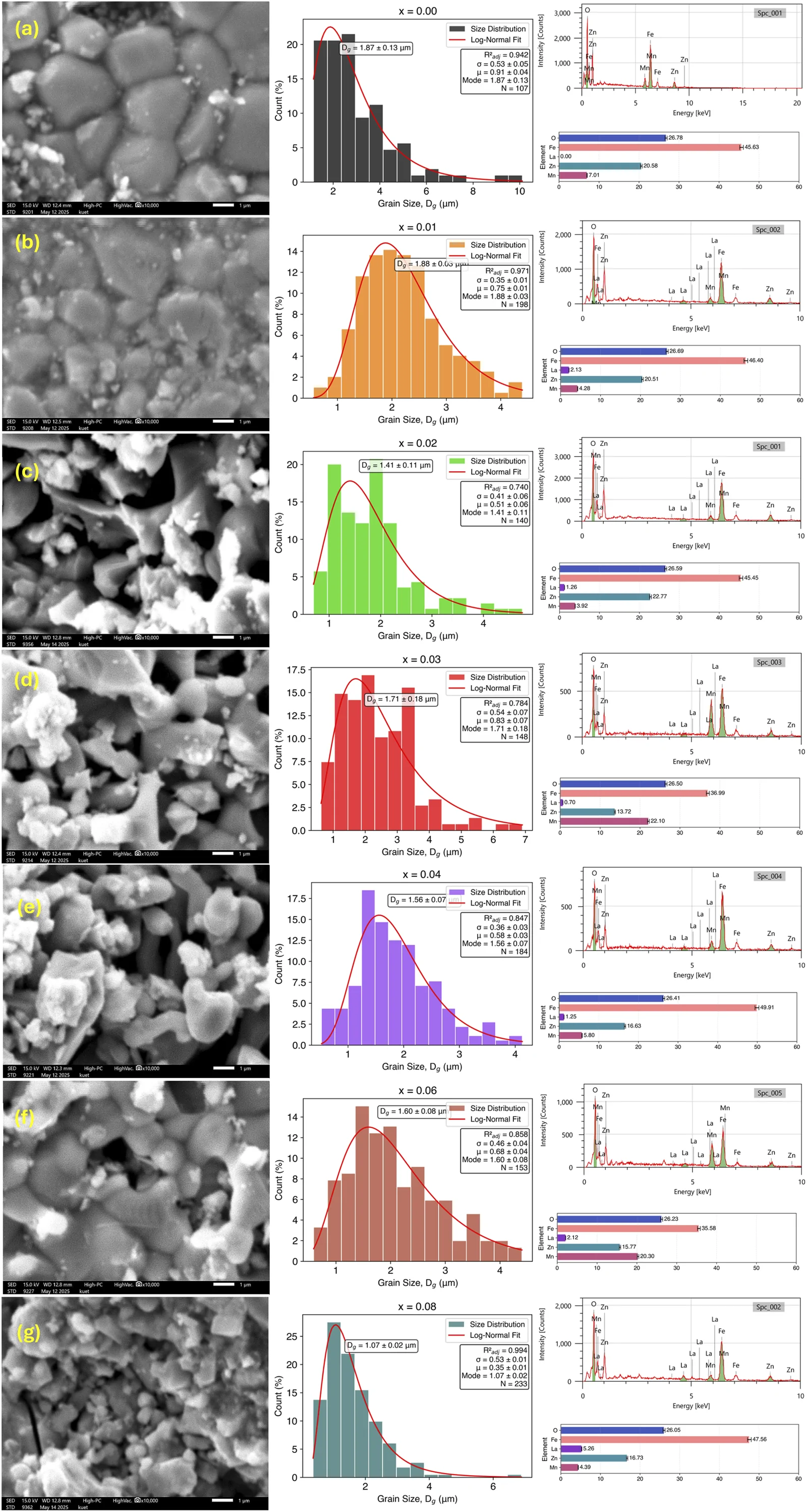
Microstructural morphology, grain size distribution histograms, and energy-dispersive X-ray spectroscopy (EDXS) elemental profiles of MZLF composites (a–d), displaying SEM micrographs (left), corresponding log-normal grain size distribution fits (middle), and elemental concentration spectra with bar charts (right).

Quantitative grain statistics (Table S8 and Fig. 4a–g, middle) show overall grain refinement with increasing La^3+^ content, from 1.872 µm at *x* = 0.00 to 1.072 µm at *x* = 0.08. A local increase occurs at *x* = 0.03 (1.706 µm), most likely because larger facets are exposed on the fracture surface. Image-derived pore descriptors (Table S9 and Fig. S6a–g) confirm that both pore fraction and pore geometry change noticeably with La^3+^ content. The absolute porosity measured by SEM is lower than the bulk porosity because SEM records a two-dimensional surface fraction, whereas density-based porosity reflects the three-dimensional volume and includes closed pores.^65,66^ Full details of the segmentation procedures, parameter choices, and additional masks or thickness maps used to maintain consistency across the series are given in the SI (ST3).

#### 3.2.2 EDXS elemental analysis and compositional homogeneity

EDXS area analysis was performed to confirm the presence of Mn, Zn, La, Fe, and O in the series and to evaluate approximate compositional uniformity. The normalized mass-percent results are summarized in Table S10, and representative spectra and scan areas are shown in Fig. 4 (top-right) and S7. For *x* = 0.00, La is absent as expected, and the detected Mn, Zn, Fe, and O signals confirm the base ferrite chemistry. For La^3+^ substituted compositions, La is detected in all doped samples.

While EDXS confirms elemental presence, the quantitative agreement with nominal stoichiometry is not uniform across the series. Some compositions show large deviations in observed Mn and Zn values, for example *x* = 0.03 and *x* = 0.06, where Mn appears strongly overestimated and Zn underestimated. This behavior can be attributed to standard limitations of SEM–EDXS for complex oxides. These include underestimation of oxygen due to absorption of low-energy X-rays, matrix effects, and normalization artifacts that can inflate heavier-element fractions when oxygen is underestimated.^67,68^ The interaction volume at 15 kV can mix signals from multiple grains and phases, and local compositional heterogeneity can also influence the result.^69^ Furthermore, lanthanide-related peak overlap and quantification difficulty has been reported in related oxide systems, and alternative methods such as TEM-EELS are often needed for high-accuracy lanthanide quantification.^70,71^

Therefore, in this work EDXS is used primarily as a qualitative to semi-quantitative confirmation tool. The main structural conclusions are drawn from XRD/Rietveld phase analysis, while the microstructure conclusions are drawn from SEM morphology and image statistics. Even with these constraints, the combined SEM/EDXS results support that La^3+^ substitution modifies densification, pore morphology, and grain-scale statistics in a way that is consistent with the bulk density trends and the phase evolution inferred from diffraction.

### 3.3 Static magnetic properties

Room-temperature *M*–*H* loops for the La^3+^ substituted Mn–Zn ferrites are shown in Fig. 5a, and the extracted magnetic parameters are summarized in Table 2. All compositions exhibit narrow hysteresis loops with comparatively low coercivity and low remanence, which is characteristic of soft ferrimagnetic behavior.^12,72^ This soft response is expected for Mn–Zn ferrites sintered at high temperature, where domain-wall motion contributes strongly to magnetization reversal and where magnetocrystalline anisotropy is relatively low compared with hard ferrites. Across the composition series, the loops approach technical saturation within the applied field window (±15 kOe). However, several samples still show a gradual high-field approach to saturation, which indicates that rotation processes and anisotropy-related terms remain relevant at high field.

**Fig. 5:**
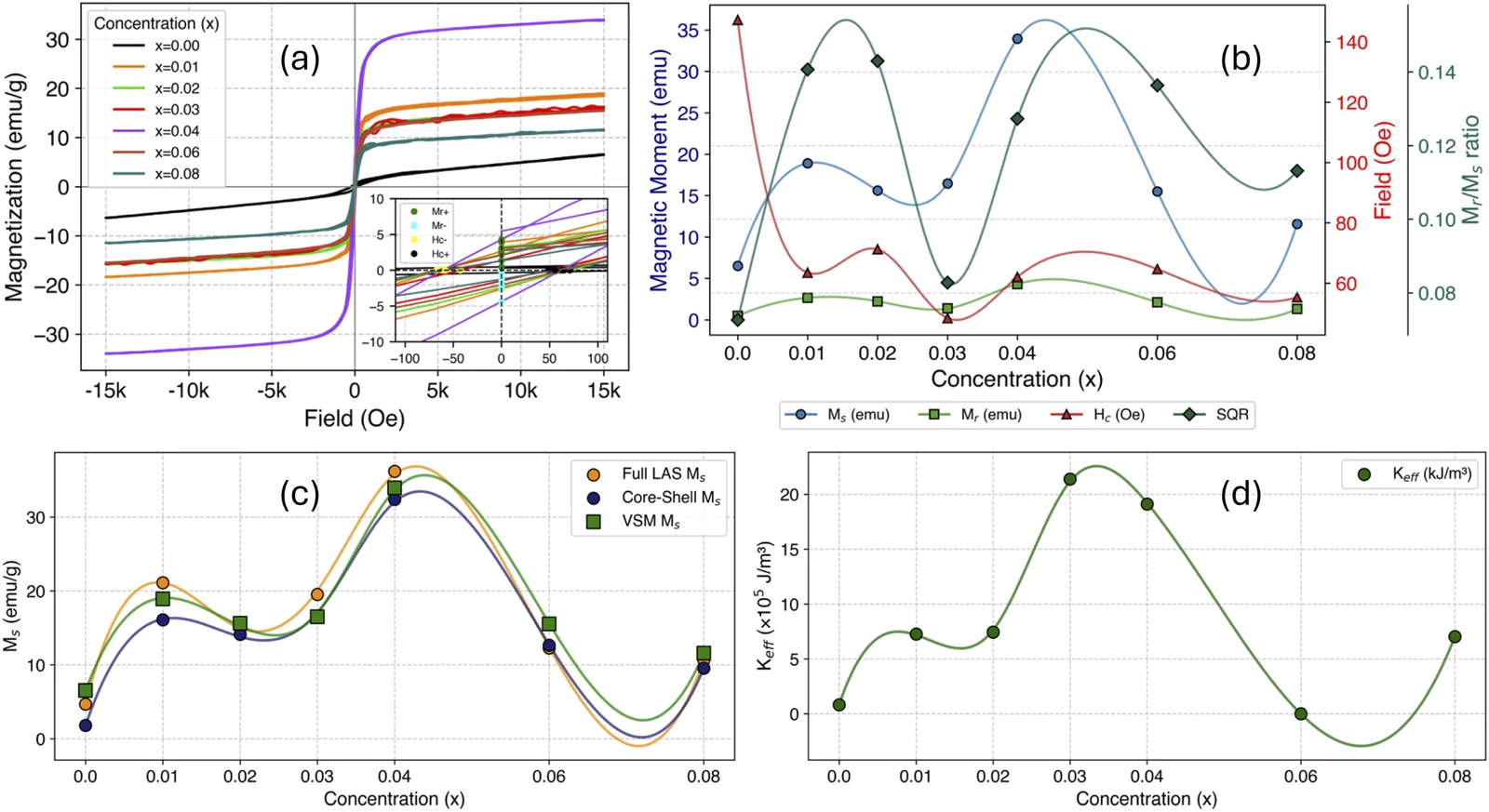
Room-temperature magnetic properties of MZLF composites showing (a) magnetic hysteresis (*M*–*H*) loops measured up to 15 kOe with an inset highlighting the low-field coercive region, (b) compositional dependence of saturation magnetization, remanent magnetization, coercivity, and squareness ratio, (c) comparison of experimental VSM values against theoretical predictions from the Law of Approach to Saturation (LAS) and core–shell models, and (d) evolution of the calculated effective magnetic anisotropy constant.

**Table 2:** Static magnetic parameters from VSM hysteresis loops at room temperature^a^

*x*	0.00	0.01	0.02	0.03	0.04	0.06	0.08
*M*_s_	6.53	18.93	15.61	16.50	33.96	15.53	11.59
*M*_r_	0.49	3.44	2.84	1.79	5.25	2.59	1.62
*H*_c_	139.09	63.72	70.47	51.83	63.41	65.41	56.72
SQR	0.08	0.18	0.18	0.11	0.15	0.17	0.14

a *M*_s_: saturation magnetization (emu g^−1^); *M*_r_: remanent magnetization (emu g^−1^); *H*_c_: coercivity (Oe); SQR: squareness ratio (*M*_r_/*M*_s_).

#### 3.3.1 Saturation magnetization trend and mechanistic interpretation

The saturation magnetization (*M*_s_) shows a strong and non-monotonic dependence on La^3+^ content. The *x* = 0.00 sample shows *M*_s_ = 6.53 emu g^−1^. With La^3+^ substitution, *M*_s_ increases sharply for *x* = 0.01 to 18.93 emu g^−1^, then remains relatively high for *x* = 0.02 (15.61 emu g^−1^) and *x* = 0.03 (16.50 emu g^−1^). The highest *M*_s_ is obtained for *x* = 0.04, where *M*_s_ increases to 33.96 emu g^−1^. At higher La^3+^ contents, *M*_s_ decreases, where *x* = 0.06 shows 15.53 emu g^−1^ and *x* = 0.08 decreases to 11.59 emu g^−1^.

This non-linear trend suggests competing effects of La^3+^ addition on the net ferrimagnetic moment. In spinel ferrites, the net magnetization is governed by the difference between the B-site and A-site sublattice moments (*M*_B_ − *M*_A_).^73^ A modest La^3+^ substitution can indirectly modify cation distribution between A and B sites, influence Fe^2+^/Fe^3+^ ratio, and alter superexchange strength through local lattice distortion.^74^ These factors can increase *M*_B_ − *M*_A_ and raise *M*_s_. At higher La^3+^ contents, several mechanisms can reduce *M*_s_. First, La^3+^ is non-magnetic, so substitution can dilute magnetic sublattices if La^3+^ enters (or disrupts) Fe-containing sites.^75,76^ Second, local distortion can increase spin canting on the B sublattice (Yafet–Kittel type triangular spin arrangement), which reduces the collinear component of magnetization even when the number of magnetic ions remains similar.^18,77,78^ Third, the appearance of a secondary *Pnma* phase for *x* ≥ 0.02 shows that La^3+^ incorporation into the spinel lattice remains incomplete at higher substitution levels, and the accompanying La^3+^-rich grain-boundary segregation together with minor secondary-phase formation may lower the effective ferrimagnetic volume fraction of the dominant spinel phase.

It is also important to consider microstructural effects. The *x* = 0.04 composition shows the highest bulk porosity, which would typically reduce the measured mass-normalized magnetization due to demagnetizing fields and reduced magnetic connectivity. The fact that *M*_s_ is still maximized at *x* = 0.04 suggests that the intrinsic sublattice contribution dominates over the porosity penalty for this composition.

Coercivity (*H*_c_) and remanence (*M*_r_) reflect both intrinsic anisotropy and extrinsic microstructure. The *x* = 0.00 sample shows the highest coercivity, with an average *H*_c_ of 159.91 Oe and low remanence (*M*_r_ ≈ 0.49 emu g^−1^). With La^3+^ substitution, coercivity decreases substantially. For *x* = 0.03, *H*_c_ decreases to 47.64 Oe, which is the minimum in the series. The *x* = 0.04 sample shows *H*_c_ = 62.92 Oe, and *x* = 0.08 shows *H*_c_ = 57.49 Oe.

The remanent magnetization also changes with composition. For *x* = 0.00, *M*_r_ = 0.495 emu g^−1^. For *x* = 0.04, *M*_r_ increases to 5.248 emu g^−1^, consistent with its higher *M*_s_. However, the squareness ratio *M*_r_/*M*_s_ remains relatively low across the series (*e.g.*, SQR ≈ 0.076 for *x* = 0.00 and ≈0.155 for *x* = 0.04). Low squareness is typical for soft ferrites and indicates that magnetization reversal is dominated by reversible processes and domain-wall motion rather than strong pinning and irreversible rotation.

Microstructure provides a consistent qualitative basis for the coercivity trend. Smaller grains can increase *H*_c_ when domain walls are pinned at grain boundaries, whereas larger grains may lower *H*_c_ by promoting easier domain-wall motion, up to the point where multi-domain behavior is fully developed. Porosity and microcracks may also act as pinning centers and increase coercivity. In the present series, *x* = 0.08 shows the smallest grain size from SEM statistics, which is consistent with a moderate *H*_c_ rather than the lowest *H*_c_. The minimum coercivity at *x* = 0.03 likely reflects a favorable balance of grain size, reduced effective pinning, and lower anisotropy-related barriers. Because grain size, pore morphology, and densification evolve simultaneously across the series, these *H*_c_ variations represent concurrent microstructural effects rather than the isolated influence of a single parameter.

#### 3.3.2 Anisotropy indicators and LAS fitting

To analyze the high-field magnetization behavior, LAS fitting was performed in the high-field region (8–10 kOe), using a full LAS model and a core–shell form.^38,79^ The fitting outcomes are summarized in Fig. 6 and Table S13, including the preferred model and the effective anisotropy constant (*K*_eff_), calculated under a uniaxial anisotropy assumption.

**Fig. 6:**
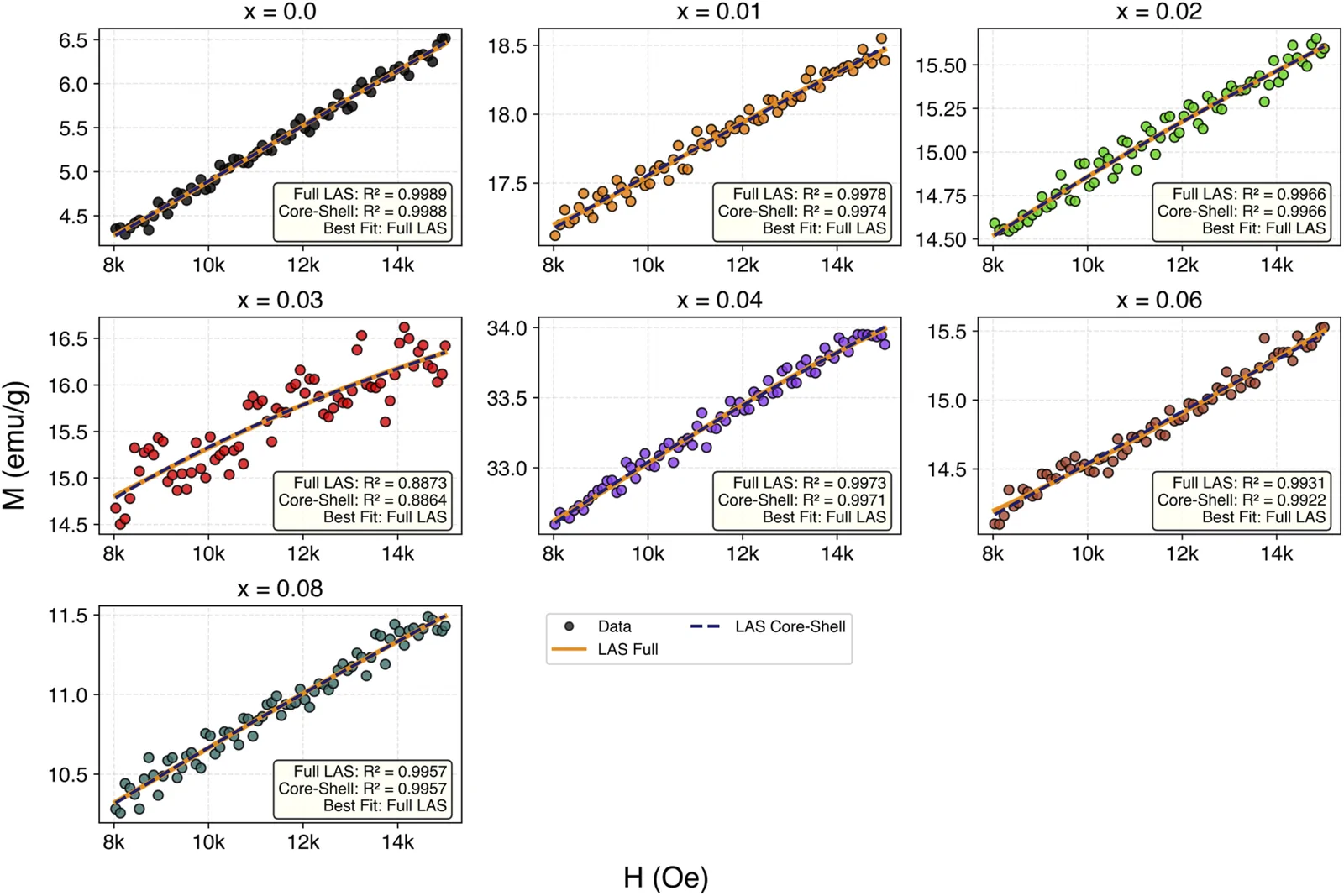
Law of Approach to Saturation (LAS) fitting plots of high-field magnetization curves comparing the LAS full model (solid orange line) and the core–shell model (dashed blue line) for MZLF composites across different lanthanum concentrations.

For most compositions, the full LAS model provides a better fit in the chosen field range. The extracted *K*_eff_ values vary strongly with composition (Fig. 5d). The *x* = 0.00 sample shows *K*_eff_ ≈ 0.82 kJ m^−3^. With La^3+^ addition, *K*_eff_ increases, where *x* = 0.01 increases to 7.27 kJ m^−3^ and *x* = 0.02 increases to 7.43 kJ m^−3^. The highest *K*_eff_ is observed at *x* = 0.03, where *K*_eff_ increases to 21.38 kJ m^−3^, while *x* = 0.04 shows 19.11 kJ m^−3^. At *x* = 0.08, *K*_eff_ decreases to 7.03 kJ m^−3^. The anisotropy field values show the same general behavior, where *x* = 0.02 and *x* = 0.03 increase to ∼14 kOe, while *x* = 0.08 decreases to ∼8 kOe.

These anisotropy trends may reflect La^3+^ induced lattice distortion, changes in local symmetry, and spin canting, all of which can increase anisotropy-related energy barriers. However, LAS fitting is sensitive to the chosen field window, the assumed anisotropy symmetry, and the presence of porosity and secondary phases. Therefore, *K*_eff_ values are interpreted as comparative indicators rather than absolute intrinsic constants. This caution is consistent with common practice in ferrite LAS analysis, where different models (full LAS, core–shell, or rotation-only fits) can yield different parameter sets.^79^

### 3.4 Broadband magnetic dynamics

#### 3.4.1 Real and imaginary parts of permeability dispersion

The frequency-dependent magnetic response was evaluated from 100 Hz to 120 MHz. The real part of permeability (*µ*′) and the imaginary part (*µ*″) are summarized in Tables S14, S15 and visualized in Fig. 7a and b for all compositions. Across the series, *µ*′ remains relatively stable over a broad frequency range, while *µ*″ decreases strongly with frequency, indicating that magnetic losses are dominated by low-frequency relaxation and are suppressed at higher frequencies.

**Fig. 7:**
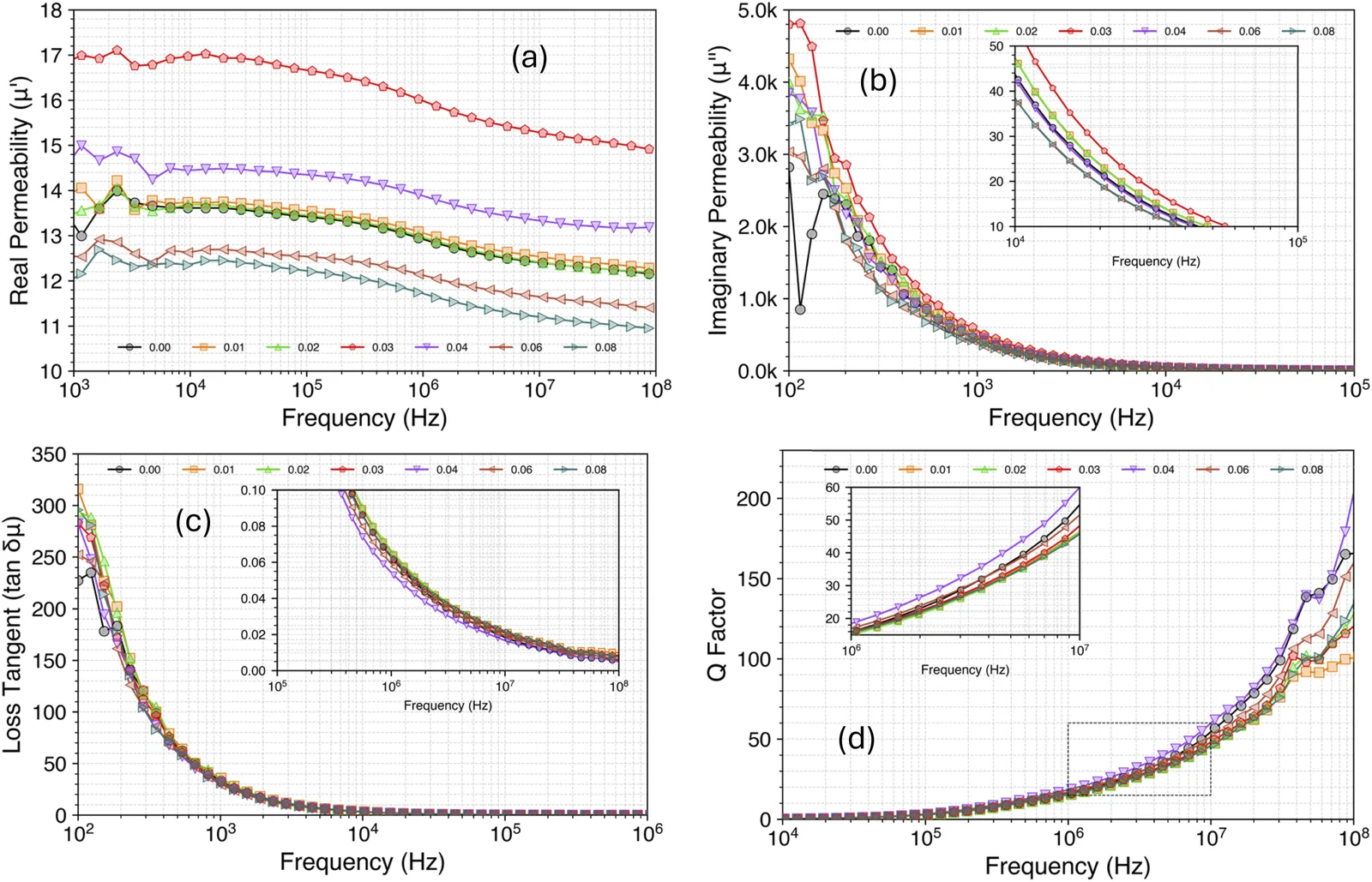
Frequency and compositional dependence of magnetic permeability parameters and loss factors for MZLF samples: (a) variation of real permeability with frequency, (b) frequency spectrum of imaginary permeability with a high-frequency magnified inset, (c) quality factor as a function of frequency with a low-frequency inset, and (d) variation of magnetic loss tangent with frequency showing a high-frequency inset.

At low frequency (100 Hz), *µ*′ varies significantly with La^3+^ content. The *x* = 0.03 sample shows the highest *µ*′ of 16.58 at 100 Hz, while *x* = 0.06 shows the lowest *µ*′ of 10.76. As frequency increases to 120 MHz, *µ*′ decreases modestly for each composition, consistent with a gradual reduction of domain-wall contribution and increased dominance of spin rotation.^80^ For example, *x* = 0.03 decreases from *µ*′ = 16.58 at 100 Hz to *µ*′ = 14.82 at 120 MHz, while *x* = 0.00 decreases from *µ*′ = 12.45 to 12.09 over the same interval. This mild dispersion suggests that the materials maintain usable permeability across the measured band.

The *µ*″ response shows a much stronger frequency dependence. For *x* = 0.03, *µ*″ decreases from 5560.79 at 100 Hz to 0.14 at 120 MHz. For *x* = 0.06, *µ*″ decreases from 4001.05 at 100 Hz to 0.07 at 120 MHz. This strong reduction reflects the transition from low-frequency loss processes associated with domain-wall motion and interfacial friction to higher-frequency regimes where loss mechanisms are weaker under the present excitation conditions. This domain-wall to spin-rotation crossover is a standard interpretation in soft ferrites, and it is consistent with the relatively stable *µ*′ at high frequency.^31,80^

#### 3.4.2 Magnetic loss tangent and quality factor

Magnetic loss tangent (tan *δ*_m_) and quality factor (*Q*) provide device-relevant loss metrics for high-frequency ferrite components. The tan *δ*_m_ values are summarized in Table S16 and Fig. 7c, while *Q* is summarized in Table S17 and Fig. 7d. At high frequency, all compositions show very low magnetic loss. At 100 MHz, tan *δ*_m_ lies in the narrow range 0.005–0.010, where the minimum occurs at *x* = 0.04 (tan *δ*_m_ = 0.005) and the maximum occurs at *x* = 0.01 (tan *δ*_m_ = 0.010). At 120 MHz, tan *δ*_m_ further decreases to 0.004–0.009, with the lowest loss shared by *x* = 0.04 and *x* = 0.06 (tan *δ*_m_ = 0.004), while *x* = 0.01 remains the highest (tan *δ*_m_ = 0.009). This confirms that La^3+^ substitution can suppress high-frequency magnetic loss in selected compositions, which is consistent with the expected reduction of domain-wall loss contribution and the increasing dominance of spin-rotation response at higher frequency in soft ferrites.^80^

The quality factor increases strongly with frequency for all samples, highlighting compositions with reduced dynamic loss. At 120 MHz, *x* = 0.06 shows the maximum *Q* of 259.65, followed by *x* = 0.04 with *Q* = 242.23, while *x* = 0.00 shows a lower *Q* of 151.22. These results indicate that *x* = 0.06 and *x* = 0.04 offer the most favorable loss-permeability balance near the upper end of the measured band. This is important for high-frequency power components, because higher *Q* is associated with lower effective core loss and improved energy efficiency under AC excitation.^81^

At low frequency, tan *δ*_m_ values are very large (for example, tan *δ*_m_ ≈ 245.25 at 100 Hz for *x* = 0.00). Such large values are expected when *µ*″ is extremely high at very low frequency and when losses are dominated by slow, quasi-static processes related to domain-wall motion and interfacial friction. For device relevance, the mid-to-high frequency region is more meaningful, and the present dataset shows strong loss suppression in this region for all compositions, with the best high-frequency performance observed near *x* = 0.04–0.06.

#### 3.4.3 Cole–Cole arcs, magnetic modulus, and relaxation times

Magnetic relaxation was examined using the magnetic modulus formalism and Cole–Cole representations. The real and imaginary parts of the magnetic modulus (*Mµ*′ and *Mµ*″) are summarized in Tables S18, S19 and plotted in Fig. 8a and b. In this representation, the modulus remains close to zero at low frequency and increases at higher frequency, which helps emphasize the relaxation window associated with domain-wall and spin-rotation dynamics.

**Fig. 8:**
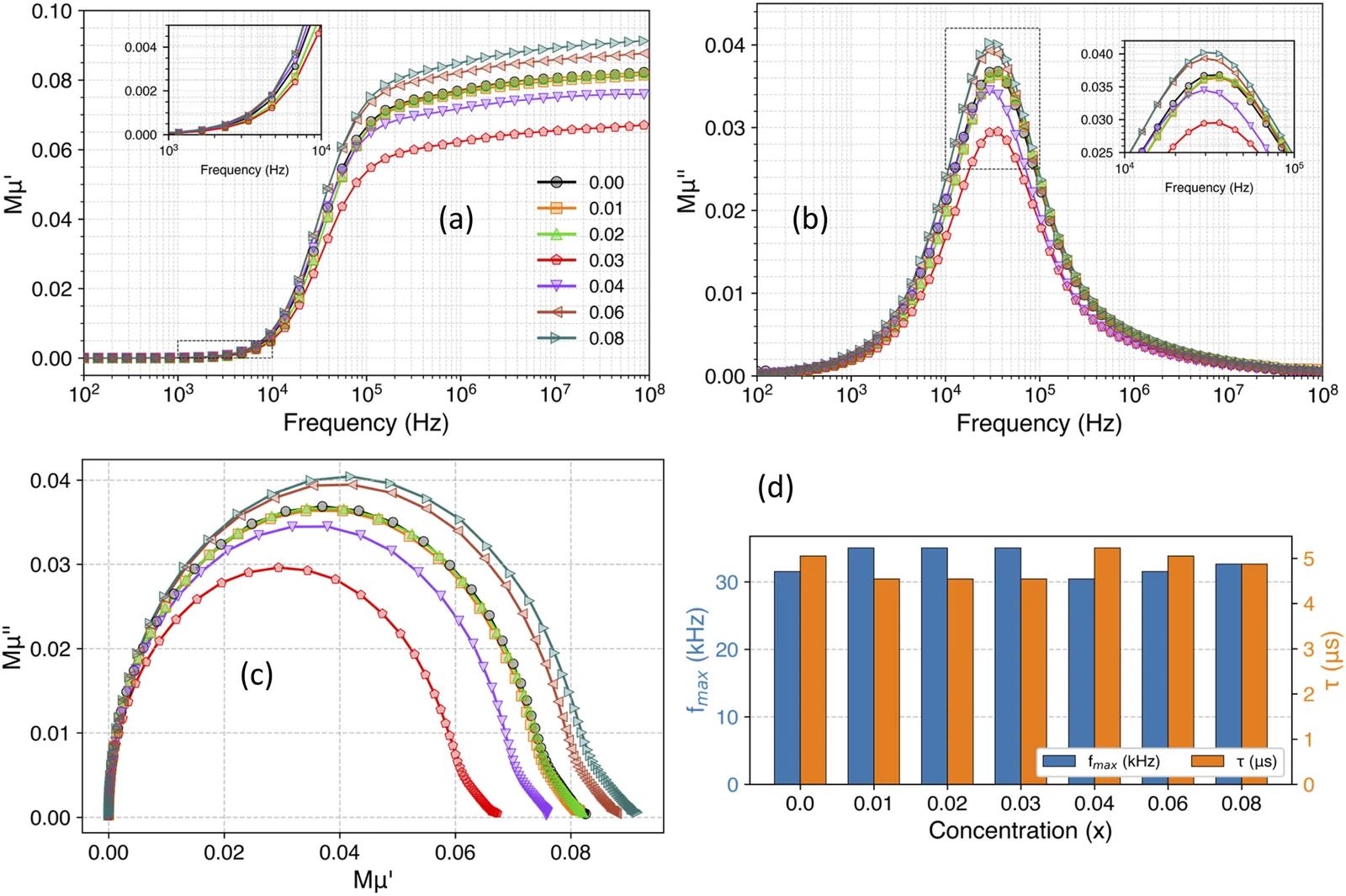
Frequency and compositional dependence of complex magnetic modulus parameters for MZLF samples: (a) variation of real magnetic modulus with frequency showing a low-frequency magnified inset, (b) imaginary magnetic modulus with a magnified peak inset, (c) magnetic modulus Cole–Cole plots, and (d) variation of maximum relaxation frequency and relaxation time.

The Cole–Cole plots of magnetic modulus are shown in Fig. 7c. All compositions form smooth semicircular arcs with their centers depressed below the real axis. This depressed-arc feature indicates a distribution of relaxation times and confirms non-Debye-type magnetic relaxation. Such behavior is expected for polycrystalline ferrites with heterogeneous microstructures, where multiple contributions overlap, including domain-wall pinning at grain boundaries, porosity-related demagnetizing effects, and local compositional disorder.

Relaxation frequencies (*f*_max_), and relaxation times (*τ*) were extracted from the frequency position of the modulus loss peak (*Mµ*″ maximum) using *τ* = 1/(2π*f*_max_), and the results are summarized in Table S20. The relaxation times lie in the microsecond range and show only weak composition dependence. For *x* = 0.00, *f*_max_ = 36 279 Hz and *τ* = 4.39 × 10^−6^ s. For *x* = 0.06, *f*_max_ creases to 31 529 Hz and *τ* increases to 5.05 × 10^−6^ s. This shift indicates slightly slower magnetic relaxation at *x* = 0.06, which can be linked to stronger resistive damping of domain-wall motion and/or a modified pinning potential distribution. The arcs for all compositions return to the baseline in the modulus Cole–Cole plane, indicating that the measurement window (100 Hz to 120 MHz) captured the complete dominant magnetic relaxation semicircle. This contrasts with the dielectric modulus response, where arc truncation can occur when the main relaxation lies outside the accessible frequency band.

### 3.5 Wideband dielectric response

#### 3.5.1 Dielectric dispersion and Maxwell–Wagner/Koop's model interpretation

The dielectric response of the MZLF series was investigated from 100 Hz to 120 MHz. The real part of the dielectric constant (*ε*′) and the imaginary part (*ε*″) are summarized in Tables S21, S22, respectively, and are plotted in Fig. 9a and b. All compositions show strong dielectric dispersion, characterized by very high low-frequency permittivity followed by a steep decrease with increasing frequency. This behavior is typical for polycrystalline ferrites and is commonly interpreted using Maxwell–Wagner interfacial polarization combined with Koop's two-layer model.^82–84^

**Fig. 9:**
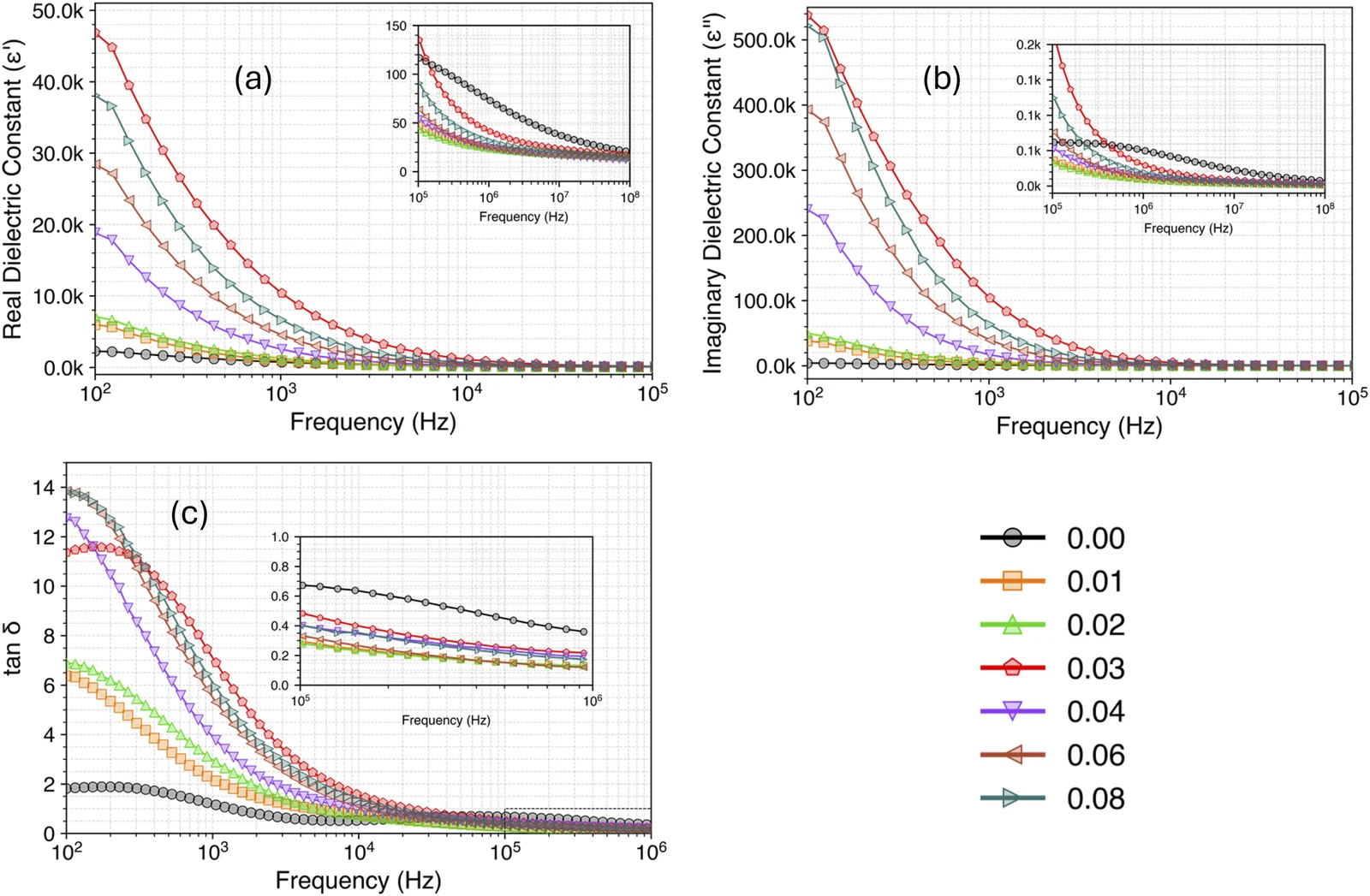
Frequency and compositional dependence of complex dielectric parameters for MZLF samples: (a) variation of real dielectric constant with frequency showing a high-frequency magnified inset, (b) frequency spectrum of imaginary dielectric constant with a high-frequency magnified inset, and (c) variation of dielectric loss tangent with frequency.

At 100 Hz, *ε*′ varies strongly with composition. The *x* = 0.00 sample shows *ε*′ = 2372.52. With La^3+^ substitution, *ε*′ increases, where *x* = 0.01 increases to 6498.85 and *x* = 0.02 increases to 7735.17. The largest *ε*′ is observed for *x* = 0.03, where *ε*′ increases to 50 599.24. For *x* = 0.04, *ε*′ decreases to 20 659.49, while *x* = 0.06 increases again to 31 134.19 and *x* = 0.08 increases to 42 076.90. As frequency increases, *ε*′ decreases markedly for all compositions. At 120 MHz, *ε*′ decreases to 19.11 for *x* = 0.00 and decreases to 15.99 for *x* = 0.03, indicating that high-frequency permittivity values converge into a narrow range.

The *ε*″ response shows the same interfacial character. At 100 Hz, *ε*″ is 4315.90 for *x* = 0.00 and increases strongly for La^3+^ substituted compositions, reaching 5.74 × 10^5^ for *x* = 0.03 and 5.82 × 10^5^ for *x* = 0.08. At 120 MHz, *ε*″ decreases to 6.77 for *x* = 0.00 and decreases to 3.43 for *x* = 0.03. The large low-frequency *ε*″ values indicate strong energy dissipation associated with grain-boundary dominated charge accumulation and hopping conduction, while the sharp reduction at high frequency reflects the inability of charge carriers and interfaces to follow the alternating field.

Within the Maxwell–Wagner/Koop's framework, the grains are comparatively more conductive, while the grain boundaries are more resistive. At low frequency, charge carriers pile up at these resistive interfaces, producing high interfacial polarization and high apparent *ε*′. With increasing frequency, interfacial charge build-up becomes incomplete, and the response transitions toward the intrinsic grain contribution, producing lower and more stable *ε*′ and *ε*″. The strong composition dependence at low frequency indicates that La^3+^ substitution modifies the grain-boundary barrier height and defect chemistry, consistent with the impedance-derived grain and grain-boundary resistances discussed in a later section.

#### 3.5.2 Dielectric loss tan *δ* trends and stabilization at high frequency

The dielectric loss tangent (tan *δ*) is summarized in Table S23 and plotted in Fig. 9c. All compositions show high loss at low frequency and reduced loss at high frequency. At 100 Hz, tan *δ* for *x* = 0.00 is 1.82 and increases for La^3+^ substituted compositions, reaching 11.36 for *x* = 0.03 and 13.85 for *x* = 0.06. This indicates that low-frequency loss is dominated by conductive and interfacial processes, which intensify with La^3+^ substitution for most compositions.^85^

As frequency increases tan *δ* decreases sharply, at 100 kHz tan *δ* decreases to 0.68 for *x* = 0.00 and decreases to 0.48 for *x* = 0.03, indicating partial suppression of interfacial dissipation. At 1 MHz, tan *δ* decreases to 0.35 for *x* = 0.00 and decreases to 0.21 for *x* = 0.03. Above 1 MHz, tan *δ* becomes nearly frequency independent within the reported table values. For example, at 10–120 MHz, tan *δ* remains at 0.35 for *x* = 0.00 and remains at 0.21 for *x* = 0.03. This high-frequency stabilization suggests that the dominant loss mechanism transitions away from slow interfacial polarization toward faster polarization modes with weaker dissipation in the measured band. From a device perspective, this behavior indicates that dielectric losses are a limiting factor at low frequency, but at MHz-range operation, the samples exhibit comparatively stable loss tangents, which is relevant for high-frequency components where combined magnetic and dielectric dissipation contributes to heating.

To separate electrode/interfacial effects from bulk relaxation and to emphasize conductivity-related relaxation, the dielectric response was further examined using the electric modulus formalism. The real modulus (*M*′) and imaginary modulus (*M*″) are summarized in Tables S24, S25 and plotted in Fig. 10a and b. In modulus representation, low-frequency values are near zero for most compositions, indicating that long-range charge motion and electrode polarization effects are suppressed in M* and that bulk relaxation processes become more visible.

**Fig. 10:**
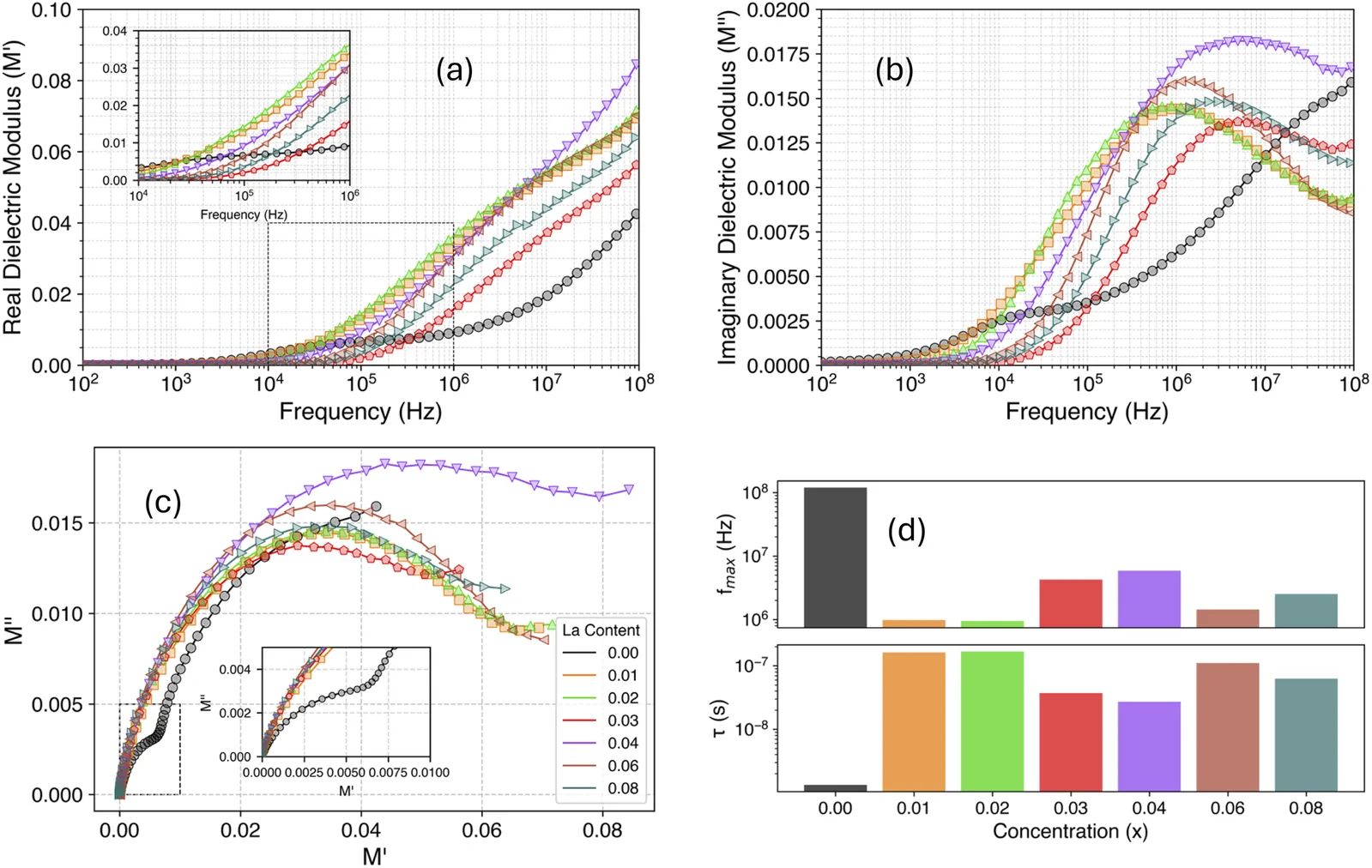
Frequency and compositional dependence of complex dielectric modulus parameters for MZLF samples: (a) variation of real dielectric modulus with frequency showing a high-frequency magnified inset, (b) frequency spectrum of imaginary dielectric modulus tracking relaxation peaks, (c) dielectric modulus Cole–Cole plots with a low-value origin inset, and (d) variation of maximum relaxation frequency and relaxation time with lanthanum concentration.

*M*′ increases with frequency for all samples and approaches a plateau at high frequency, consistent with a transition from long-range conduction to localized relaxation. *M*″ shows broad peaks and depressed-arc features when plotted in the Cole–Cole plane (Fig. 10c). These depressed arcs indicate non-Debye relaxation with a distribution of relaxation times, which is typical for heterogeneous ceramics with grain and grain-boundary contributions.

The Cole–Cole modulus analysis indicates a dominant grain-boundary contribution across the main frequency window, visible as the primary semicircular arc. A secondary grain-core contribution appears as a compressed arc near the origin at higher frequencies (inset region), which indicates that the grain interior has higher conductivity and relaxes at higher frequency than the grain boundaries. This separation is consistent with Koop's model and supports the use of equivalent-circuit separation in impedance analysis.^86^

Relaxation parameters extracted from the modulus peak positions are summarized in Table S26 and plotted in Fig. 10d. The dielectric relaxation frequency (*f*_max_) and relaxation time show strong composition dependence. For *x* = 0.00, *f*_max_ reaches 120 MHz and *τ* decreases to 1.33 × 10^−9^ s, suggesting the dominant polarization process may have shifted beyond the low-frequency interfacial regime to likely initiate the high-frequency Lorentz-type resonance state. For *x* = 0.01, *f*_max_ decreases to ∼981.3 kHz and *τ* increases to 1.62 × 10^−7^ s. For *x* = 0.02, *f*_max_ ≈ 947.5 kHz and *τ* increases to 1.68 × 10^−7^ s. For *x* = 0.03, *f*_max_ increases to 4.28 MHz and *τ* decreases to 3.72 × 10^−8^ s. For *x* = 0.04, *f*_max_ increases to 5.87 MHz and *τ* decreases to 2.71 × 10^−8^ s. For *x* = 0.06, *f*_max_ decreases to 1.44 MHz and *τ* increases to 1.11 × 10^−7^ s. For *x* = 0.08, *f*_max_ increases to 2.53 MHz and *τ* decreases to 6.29 × 10^−8^ s.

These *τ* values show that La^3+^ substitution shifts dielectric relaxation into different frequency windows, which supports the conclusion that La^3+^ modifies grain-boundary barrier heights and hopping dynamics. The non-monotonic trend again suggests competing mechanisms, including lattice distortion, defect chemistry changes, and porosity/microstructure effects. Similar shifts in modulus relaxation with rare-earth substitution have been reported in ferrite ceramics and are often linked to changes in Fe^2+^/Fe^3+^ hopping probability and grain-boundary resistive networks.

### 3.6 Impedance spectroscopy and equivalent circuit interpretation

Nyquist plots and equivalent circuit Nyquist plots (Fig. 11) were fitted using *R*_s_–(*R*_g_‖CPE_g_)–(*R*_gb_‖CPE_gb_), and the extracted parameters are listed in Table S27. *R*_s_ ranges from 6.38 to 37.54 Ω across the series, confirming comparatively minor contact and fixture contributions. The microstructure-sensitive barriers *R*_g_ and *R*_gb_ vary strongly with La^3+^ substitution, fundamentally altering the dominant transport mechanism. The undoped ferrite is heavily grain-boundary dominated (*x* = 0.00: *R*_g_ = 717.71 kΩ; *R*_gb_ = 2754.43 kΩ), which is consistent with classic Maxwell–Wagner type interfacial blocking. However, La^3+^ substitution rapidly redistributes this barrier network. For all substituted compositions (*x* ≥ 0.01), a barrier inversion occurs where the grain interior becomes substantially more resistive than the grain boundary. This boundary weakening reaches a minimum at *x* = 0.04 (*R*_gb_ = 240.74 kΩ). At the highest substitution level (*x* = 0.08), overall resistivity increases significantly, but the grain interior heavily dominates the barrier network (*R*_g_ = 6824.02 kΩ; *R*_gb_ = 1015.71 kΩ). The physical assignment of these elements is validated by the extracted effective capacitances: grain capacitances (*C*_g,eff_) remain strictly in the picofarad range (83.48–146.27 pF), whereas grain-boundary capacitances (*C*_gb,eff_) exhibit values three orders of magnitude larger in the nanofarad range (13.34–72.31 nF). The fitted CPE depression factors (*α*) indicate that La^3+^ substitution also modifies the relaxation distribution. The grain interiors maintain non-Debye, distributed relaxation across the entire series (*α*_g_ = 0.74–0.81). In contrast, the grain boundaries show highly variable behavior. While low and intermediate substitutions exhibit distributed boundary relaxation (*α*_gb_ = 0.63–0.77), the grain boundaries at *x* = 0.04 and *x* = 0.08 transition to ideal Debye-like capacitive responses (*α*_gb_ = 1). Correspondingly, the fitted relaxation frequencies capture a systematic slowing of the grain-core response with La^3+^ addition, where *f*_g_ drops monotonically from 15.16 kHz at *x* = 0.00 to 2.01 kHz at *x* = 0.08. Grain-boundary relaxation (*f*_gb_) remains highly localized in the low-frequency window (23.49–91.44 Hz), peaking at *x* = 0.04 and *x* = 0.06 in direct agreement with the minimized *R*_gb_ and enhanced interfacial polarization observed in the low-frequency dielectric spectra.

**Fig. 11:**
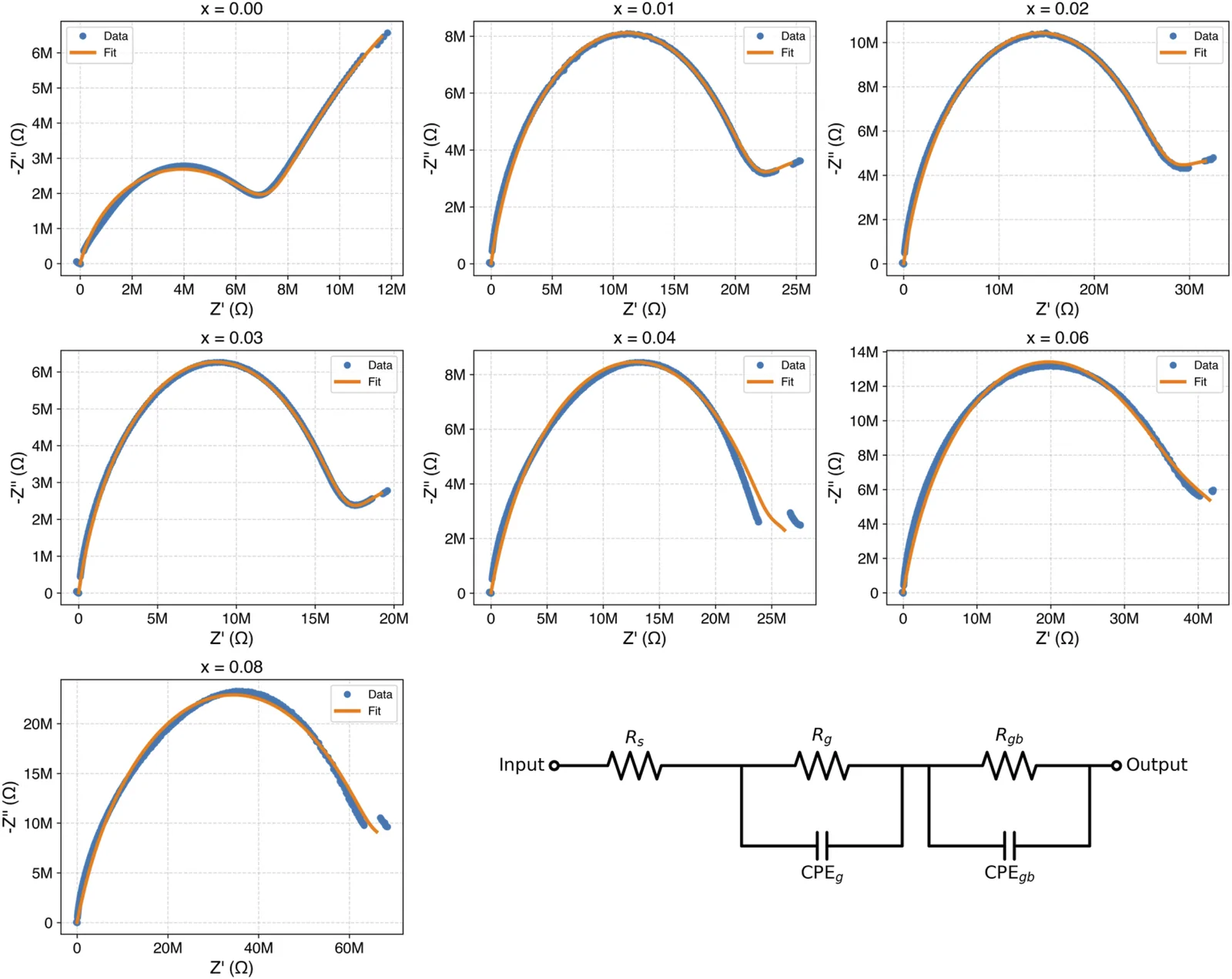
Nyquist plots of MZLF samples with varying lanthanum concentrations showing the experimental data (blue dots) alongside their theoretical fits (orange lines), and the equivalent electrical circuit model used for the simulation.

### 3.7 Magneto-dielectric coupling

To couple the magnetic and dielectric dispersions into one compact view, we evaluated impedance-form indicators based on the ratio 
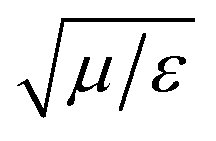
. Achieving a balanced magneto-dielectric response through microstructural and interfacial design is a proven strategy for optimizing impedance matching and tuning electromagnetic wave attenuation bandwidths.^87,88^ For instance, recent studies demonstrate that tuning the synergy between dielectric polarization and magnetic loss across multiple interfaces,^88^ or modulating semiconductor-network conductivity,^87^ profoundly shifts the frequency dispersion and enhances overall wave dissipation. In heterogeneous ferrites, this impedance ratio is particularly useful because it quickly shows whether the dynamic response within a given frequency window is dominated by dielectric interfacial polarization (large *ε*′) or by magnetic permeability (*µ*′). For the real-part approximation, we use 
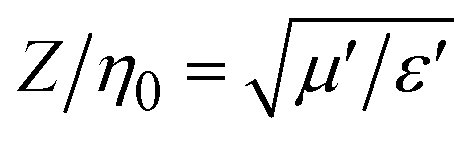
, where *η*_0_ is the free-space impedance.^38,89,90^

At low frequency, *Z*/*η*_0_ is very small for all compositions because Maxwell–Wagner/Koop's interfacial polarization strongly amplifies *ε*′ while *µ*′ changes only moderately (Fig. 12a). For example, at 100 Hz *Z*/*η*_0_ decreases from 0.0724 (*x* = 0.00) to 0.0181 (*x* = 0.03), while *ε*′ increases from 2372.5 to 50 599 with *µ*′ changing from 12.45 to 16.58. Therefore, the low *Z*/*η*_0_ values in the Hz–kHz range should be interpreted as signatures of strong grain-boundary charge accumulation rather than impedance matching to free space. With increasing frequency, *ε*′ collapses rapidly toward an intrinsic range (10.68–19.11 at 120 MHz) while *µ*′ remains weakly dispersive (10.92–14.82), so *Z*/*η*_0_ increases and approaches unity. At 120 MHz, *Z*/*η*_0_ reaches 0.795 for *x* = 0.00 and peaks at 1.114 for *x* = 0.04, consistent with the combination of low *ε*′ = 10.68 and moderate *µ*′ = 13.24 at this frequency. In practical terms, this upward shift implies that the MHz-range electromagnetic balance becomes less dominated by dielectric polarization, which is favorable for reducing strong dielectric “overloading” in magneto-dielectric designs.

**Fig. 12:**
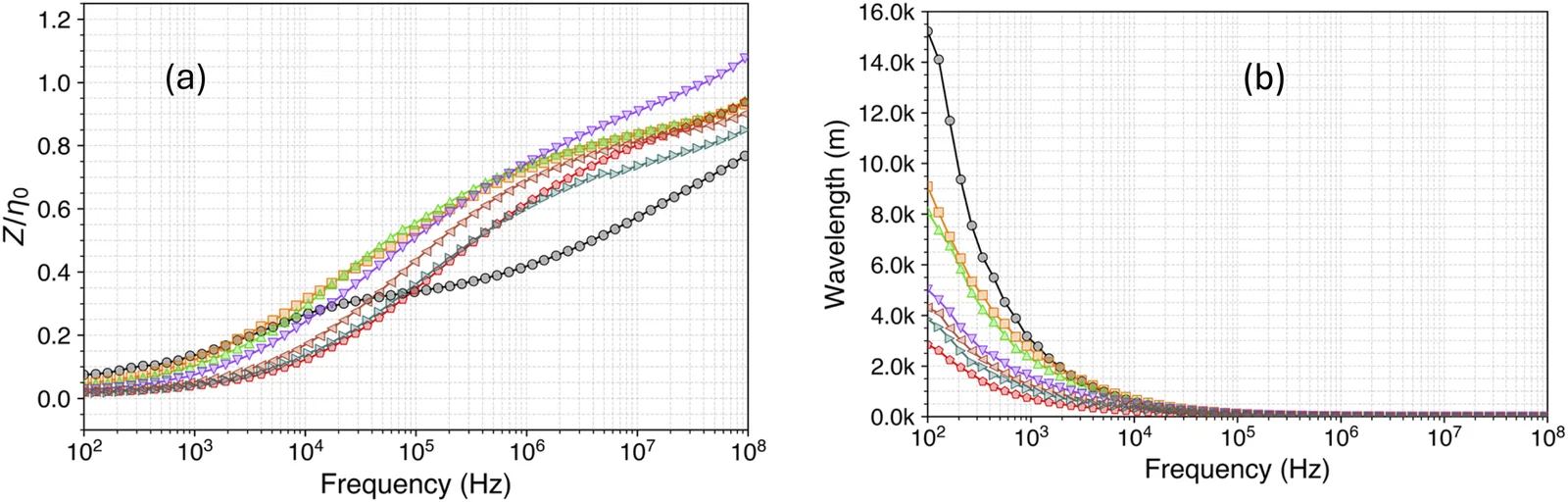
Frequency and compositional dependence of relative wave impedance and propagation wavelength for MZLF samples: (a) variation of normalized impedance with frequency, and (b) variation of wavelength with frequency.

Finally, the effective wavelength *λ*_eff_ provides a propagation-scale representation of the same coupled response.^38,90^ Using 
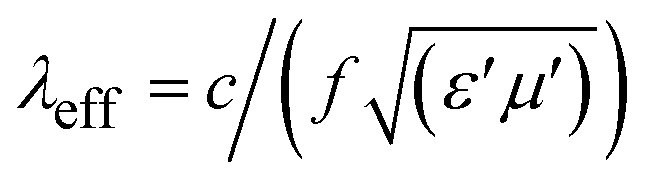
 as a practical approximation, *λ*_eff_ decreases strongly with frequency and is highly composition sensitive in the Maxwell–Wagner regime.^38^ At 100 Hz, *λ*_eff_ is 17 442 m for *x* = 0.00, 3273 m for *x* = 0.03, and 4372 m for *x* = 0.08 (Fig. 12b), reflecting the large low-frequency variation in *ε*′. At high frequency the compositions converge as *ε*′ and *µ*′ converge, and *λ*_eff_ falls into a narrow band (120 MHz: about 0.16–0.20 m across the series). Overall, these coupled indicators confirm that La^3+^ substitution primarily tunes the grain-boundary polarization network at low frequency, while the MHz-range response becomes less composition sensitive once interfacial processes freeze out.

### 3.8 Limitations and future directions

This study provides a wideband structure–microstructure–property correlation for La^3+^ substituted Mn–Zn ferrites. The measurement window (100 Hz to 120 MHz) captures the main relaxation processes in this series and enables consistent comparison across compositions, although additional fixture-corrected work at the highest frequencies would further improve the accuracy of derived transport quantities. The main interpretations are supported by impedance fitting and modulus analysis, while some secondary parameters (for example WH strain, LAS, and SEM-based 2D porosity) should be viewed as comparative descriptors rather than absolute constants. Future work will strengthen the mechanism-to-application link by adding temperature-dependent impedance for activation energies, complementary defect and cation-state probes (such as XPS or Mössbauer), and targeted sintering optimization to improve densification while preserving the beneficial grain-boundary controlled response.

## 4 Conclusion

This study established the composition-dependent balance between structural evolution, interfacial relaxation, and wideband electromagnetic properties in La^3+^ substituted Mn–Zn spinel ferrites. Rietveld refinement and quantitative structural analysis confirmed the dominance of the cubic spinel phase (*Fd*3̄*m*), with trace secondary phase formation (*Pnma*) influencing the microstructural evolution at *x* ≥ 0.02. Rather than acting solely as a bulk lattice modifier, wideband equivalent-circuit modeling demonstrates that La^3+^ substitution actively redistributes the electrical resistance of the grain-boundary barrier network. This interfacial modification serves as the central governing factor for the wideband electromagnetic response. By shifting the frequency window of non-Debye interfacial relaxation, La^3+^ substitution effectively suppresses high-frequency magnetic dissipation. Consequently, the material properties diverge based on composition: the *x* = 0.04 composition maximizes static saturation magnetization (*M*_s_ = 33.96 emu g^−1^), while the *x* = 0.06 composition exhibits the most favorable high-frequency dynamic response, yielding the lowest magnetic loss tangent and highest quality factor near 120 MHz. While practical core-loss measurements are required to definitively establish device-level performance, these isolated material parameters demonstrate that targeted La^3+^ substitution is a highly effective strategy for tuning the high-frequency dissipation window in structural ferrites.

## Author contributions

Sabbir Hasan Munna: sample preparation, materials characterization, data analysis and interpretation, and manuscript writing (original draft), coding and software (data processing, fitting, and visualization). Kazi Afsana Rahman: data analysis, interpretation, and manuscript writing (review and editing). Prof. Dr Md. Alamgir Hossain: supervision, project administration, and overall guidance on experimental design and manuscript preparation.

## Conflicts of interest

The authors declare that they have no known competing financial interests or personal relationships that could have appeared to influence the work reported in this paper.

## Supplementary Material

RA-OLF-D6RA09155E-s001

## Data Availability

The data supporting the findings of this study are available in the supplementary information (SI). Supplementary information: experimental data and analysis presented in the figures and tables. See DOI: https://doi.org/10.1039/d6ra09155e.
