# Supplementary material for: Improved wideband electromagnetic performance in La3+ substituted Mn–Zn spinel ferrites: structure–microstructure regulation of grain-boundary non-Debye relaxation
Source: RSC Adv. 2026 Nov 5. Online ahead of print. doi: 10.1039/d6ra09155e (PMC13736792; doi:10.1039/d6ra09155e)
Supplement: Supplementary file 1 [file RA-OLF-D6RA09155E-s001.pdf]

Supplementary Information (SI) for RSC Advances.
This journal is © The Royal Society of Chemistry

Improved Wideband Electromagnetic Performance in La^{3+} Substituted Mn-Zn Spinel Ferrites: Structure-Microstructure Regulation of Grain-Boundary Non-Debye Relaxation

Sabbir Hasan Munna[1], Kazi Afsana Rahman[1], Md. Alamgir Hossain[1]

Solid State Physics Laboratory, Dept of Physics
Khulna University of Engineering & Technology
Khulna-9203, Bangladesh
Corresponding Author mail: liton@phy.kuet.ac.bd

This Supplementary Information provides additional experimental details, raw and processed datasets, and extended analysis results that support the main manuscript. It includes XRD background subtraction and peak indexing outputs, Nelson-Riley lattice parameter fitting plots, modified Scherrer and Williamson-Hall fitting plots, Rietveld refinement outputs and reliability factors, SEM image-processing and segmentation parameters, grain size distribution histograms, pore-network statistics, VSM post-processing steps, impedance Nyquist fitting and equivalent-circuit parameters, and frequency-dependent magnetic and dielectric property tables. All measurements were performed at room temperature unless stated otherwise. Reported uncertainties correspond to fitting or regression uncertainty as described in the Methods section. The datasets are organized by composition (x) to allow direct comparison across the series.

Technical Details (Algorithms, Libraries, and Key Equations)

ST1 Computational environment and core libraries

All data processing, fitting, and figure generation were performed in Python. The core scientific stack was used for numerical operations, regression, optimization, and plotting.

1. Data handling

   NumPy: Array math, regression, uncertainty propagation
   Pandas: Tabular data I/O, cleaning, merging, summarizing
   SciPy: Signal processing (peak finding), optimization, interpolation
   Scikit-learn: Linear regression utilities (when applied), model metrics
   Matplotlib: Publication plots

2. XRD-specific processing

   Baseline correction: Asymmetric least squares (AsLS) / Whittaker smoothing
   Peak finding: scipy.signal.find_peaks
   Peak refinement: Quadratic sub-pixel interpolation around maxima

3. Image analysis (SEM)

ImageJ: Feret diameter grain-size extraction (manual + semi-automatic segmentation)

Python post-processing: Histogram fitting, log-normal fitting, and pore-metric calculation

4. Impedance / frequency response processing

Complex arithmetic with NumPy complex arrays

Cole–Cole and modulus representations computed from $\square^{*}$, $\square^{*}$

Equivalent circuit fitting: nonlinear optimization (SciPy) or custom fitting routine

5. Software and versions:

Category	Software/Package	Version (minimum)
Language	Python	>=3.10
Interactive environment	JupyterLab	>=4.4.9
Interactive environment	IPykernel	>=7.0.0
Interactive environment	ipywidgets	>=8.1.8
Interactive environment	ipympl	>=0.9.8
Core scientific computing	NumPy	>=2.2.6
Core scientific computing	Pandas	>=2.3.3
Core scientific computing	SciPy	>=1.15.3
Core scientific computing	Statsmodels	>=0.14.5
Core scientific computing	Numba	>=0.65.0
Core scientific computing	Scikit-learn	>=1.7.2
Plotting and visualization	Matplotlib	>=3.10.7
Plotting and visualization	Seaborn	>=0.13.2
Plotting and visualization	Plotly	>=6.5.0
Image processing (SEM)	OpenCV-Python	>=4.11.0.86
Image processing (SEM)	scikit-image	>=0.25.2
Image processing (SEM)	Pillow	>=12.2.0
XRD / baseline correction	pybaselines[full]	>=1.2.1
Materials utilities	pymatgen	>=2025.10.7
File I/O	openpyxl	>=3.1.5
Utilities	requests	>=2.33.1
Diagram utility	schemdraw	>=0.21
Linear algebra utility	pentapy	>=1.4.1
Package manager	pip	>=25.2

ST2 XRD processing and analysis algorithms

ST2.1 Background subtraction (AsLS baseline correction)

XRD intensity I(2θ) was baseline-corrected by AsLS fitting. This approach models the baseline b by minimizing a weighted combination of residual error and baseline roughness:

Minimize: $S = \sum_{i=1}^{m} w_i (y_i - b_i)^2 + \lambda \sum_{i=1}^{m} \left(\Delta^2 b_i\right)^2$

where: y_i is measured intensity, b_i is baseline, w_i are asymmetric weights updated iteratively, λ is the smoothing parameter controlling baseline stiffness, Δ^2 is the second-difference operator.

Output: Corrected intensity: $I_{corr} = I - b$

ST2.2 Peak detection and indexing

Peaks were detected using SciPy find_peaks with fixed thresholds: height = 147, prominence = 50, width = 1

For each identified peak:

1. Convert 2θ to θ: θ = (2θ)/2
2. Convert θ to radians
3. Compute d-spacing by Bragg's law: $n\lambda = 2d\sin\theta$, with n = 1 and λ = 1.5406 Å (Cu Kα), $d = \frac{\lambda}{2\sin\theta}$
4. Compute $\frac{1}{d^2}$
5. Index peaks using $\sin^2\theta$ ratios for cubic spinel: $\frac{\sin^2\theta}{\sin^2\theta_1} = \frac{h^2+k^2+l^2}{h_1^2+k_1^2+l_1^2}$

ST2.3 Lattice parameter (Nelson–Riley extrapolation)

The apparent lattice parameter ai was computed for each indexed peak and extrapolated using the Nelson–Riley function:

$$f(\theta) = \frac{1}{2}\left[\left(\frac{\sin\theta}{\cos^2\theta}\right) + \left(\frac{\theta}{\cos^2\theta}\right)\right]$$

A linear regression of a_i versus $f(\theta)$ was performed. The intercept at $f(\theta) = 0$ gives the refined exact lattice parameter a_0.

Reported outputs: a_0, standard error, slope, R^2, adjusted R^2, number of peaks.

ST2.4 Crystallite size and microstrain from line broadening

Peak position refinement: Peak maxima were refined using quadratic interpolation over three points around the peak maximum:

$$x_{vertex} = x_2 + \left[\frac{(y_1 - y_3)}{[2\,(y_1 - 2y_2 + y_3)]} \right] \cdot \square x$$

FWHM estimation: FWHM was estimated by linear interpolation on both sides of the peak to find half-maximum crossing points, then: $FWHM = x_{right} - x_{left}$

Broadening term: Instrumental Broadening Correction: To obtain the true sample broadening ($\square$), the observed peak width ($\square_{obs}$) was corrected for inherent instrumental broadening ($\square_{inst}$). The instrumental broadening profile as a function of $2\square$ was determined using a highly crystalline Corundum (Al_2O_3) standard reference material. The true structural broadening was subsequently extracted and utilized for all downstream Modified Scherrer and Williamson-Hall linear regressions.

Modified Scherrer (MS) method: A log-linear regression was used:

$$\ln(\square) = m \ln\left(\frac{1}{\cos\square}\right) + c$$

Crystallite size: $D_{SM} = \frac{K\square}{e^c}$, with K = 0.9 and $\square$ = 1.5406 Å.

Williamson–Hall (WH) method: A linear regression was used:

$$\square \cos\square = 4\,\square \sin\square + (K\square/D_{WH})$$

From the fit: $\square = \frac{slope}{4}$, and $D_{WH} = \frac{(K\,\square)}{intercept}$

ST2.5 Dislocation density proxies and Burgers vector

Burgers vector for cubic spinel (approximation): $b = \frac{a_\circ}{\sqrt{2}}$

Metrics:

1) Size-based dislocation proxy: $\square = \frac{1}{D^2}$

2) Williamson–Smallman dislocation density: $\square_{WS} = \frac{2\sqrt{3}\,\square}{b\,D}$

3) Strain-only proxy: $\square_{\square} = \left(\frac{\square}{b}\right)^2$

ST2.6 Density and porosity

Unit-cell volume: $V = a_\circ^3$

X-ray density: $\square_x = \frac{ZM}{N_A V}$

where: Z = 8 for spinel, M is molar mass of formula unit, N_A is Avogadro constant.

Bulk density: $\square_b = \frac{m}{V_{pellet}}$

Porosity: $P(\%) = \left[\frac{\square_x - \square_b}{\square_x}\right] \times 100$

ST2.7 Nearest and next-nearest cation–cation separations

a. $b = \sqrt{2}\left(\frac{a}{4}\right)$

b. $c = \sqrt{11}\left(\frac{a}{8}\right)$

c. $d = \sqrt{3}\left(\frac{a}{4}\right)$

d. $e = \sqrt{3}\left(\frac{3a}{8}\right)$

e. $f = \sqrt{6}\left(\frac{a}{4}\right)$

ST2.8 Metal–oxygen distances

a. $p = a\left(\frac{5}{8} - u\right)$

b. $q = a\sqrt{3}\left(u - \frac{1}{4}\right)$

c. $r = a\sqrt{11}\left(u - \frac{1}{4}\right)$

d. $s = a\sqrt{3}\left(\frac{u}{3} - \frac{1}{8}\right)$

ST2.9 Bond angles

a. $\square_1 = \cos^{-1}\left(\frac{p^2 + q^2 - c^2}{2pq}\right)$

b. $\square_2 = \cos^{-1}\left(\frac{p^2 + r^2 - e^2}{2pr}\right)$

c. $\square_3 = \cos^{-1}\left(\frac{2p^2 - b^2}{2p^2}\right)$

d. $\square_4 = \cos^{-1}\left(\frac{p^2 + s^2 - f^2}{2ps}\right)$

e. $\square_5 = \cos^{-1}\left(\frac{p^2 + q^2 - d^2}{2rq}\right)$

ST2.10 Ionic radii

a. $r_A = (u - \tfrac{1}{4}) \cdot a \cdot \sqrt{3} - r(O^{2-})$

b. $r_B = \left(\frac{1}{4} - u\right) \cdot a - r(O^{2-})$

ST2.11 Hopping length

a. $L_A \square\ d = (\sqrt{3})\left(\frac{a}{4}\right)$

b. $L_B \square\ b = (\sqrt{2})\left(\frac{a}{4}\right)$

ST3 SEM grain-size and pore-network analysis

ST3.1 Grain size by Feret diameter

Grain size distributions were obtained in ImageJ using Feret diameter. A log-normal model was used to fit the distribution:

$$p(d) = \left[\frac{1}{d \square \sqrt{2\square}}\right] \exp\left(-\frac{\left(\ln d - \square\right)^2}{2\square^2}\right)$$

Reported: Mean grain size, uncertainty from fit, adjusted R^2, particle count N.

ST3.2 Pore segmentation strategy

Because contrast and topography vary strongly across samples, segmentation was method-adaptive:

1. Sauvola local threshold for polished surfaces
2. Watershed + EDT markers for dense micro-void fields
3. Manual fixed-intensity threshold for highly 3D fracture surfaces

Common steps: Gaussian blur (noise suppression), Morphological closing (bridge microcracks), Size filtering (remove speckle artifacts), Controlled hole filling (retain real micropores)

ST3.3 Pore metrics

Porosity (%): pore pixels / total pixels

Equivalent diameter (for each pore): $D_{eq} = 2 \square(A/\square)$

Local thickness: Mean diameter of maximal inscribed circles (thickness map)

Percolation: Connectivity test for any pore spanning top-to-bottom or left-to-right

Euler characteristic χ: Topology descriptor (isolated pores vs looped networks)

ST3.4 Technical Details

Rationale for method-adaptive porosity segmentation: A single global threshold is not robust for this sample set because the imaging contrast mechanism changes with topography. Polished surfaces (x = 0.00–0.01) show weak intensity gradients and pore contrast is mainly at grain boundaries, while fracture-dominated samples (x = 0.03–0.08) show strong

shadow valleys and specular facets. Therefore, a method-adaptive segmentation route was used to avoid systematic bias: under-segmentation of pores in compact samples and over-segmentation of shadows in rough fracture samples.

Segmentation routes used per morphology class. Three routes were applied depending on the microstructure type:

1) Sauvola local thresholding for flat/polished fields to correct mild illumination gradients while preserving boundary-defined pores (used for x = 0.00 and x = 0.01).
2) Watershed segmentation with distance-transform markers for dense micro-void fields to separate touching pores without merging adjacent features (used for x = 0.02 and x = 0.04).
3) Manual (fixed-intensity) thresholding for highly 3D fracture morphologies to isolate true deep void valleys while suppressing false positives from reflective facets (used for x = 0.03, x = 0.06, and x = 0.08).

Post-processing and comparability control: all routes used the same downstream controls: Gaussian pre-filtering (noise suppression without shifting boundaries), morphological closing (bridge micro-cracks), size filtering (remove isolated pixel artifacts), and constrained hole filling (avoid removing real isolated micro-pores trapped inside grains). This fixed pipeline keeps the extracted metrics comparable across samples, while the front-end segmentation is adapted only to handle contrast differences.

Extracted pore-network metrics and physical meaning from each segmented pore mask (Fig. Sa-g), the following descriptors were extracted (Table S9):

a) Porosity (%): 2D area fraction of pores in the analyzed field.
b) Pore size: mean and median equivalent diameter (μm), capturing the typical pore scale.
c) Local thickness (μm): mean and median channel-width indicator, sensitive to deep crater-like voids.
d) Percolation (True/False): checks whether a single connected pore spans the image (top–bottom or left–right).
e) Euler characteristic (unitless): topology indicator; large positive values indicate mostly isolated pores.

Across all compositions, percolation was not detected within the analyzed fields (Table 11), and the Euler characteristic remained positive, indicating pore space dominated by isolated features rather than a continuous open network in the 2D window. This supports later electrical interpretation, where transport is attributed mainly to grains and grain boundaries rather than continuous open-pore channels.

ST4 Magnetic hysteresis (VSM) processing

ST4.1 Smoothing and parameter extraction

M–H curves were smoothed using Savitzky–Golay filtering: window length = 7, polynomial order = 3

$$M_i = \sum_{k=-m}^{m} c_k \, M_{i+k}, \qquad m = \frac{w-1}{2}$$

Extracted parameters: M_s (positive and negative branches), M_r, H_c, loop area.

$$\text{Loop Area} = \oint |M \cdot dH| \approx \sum \left| \frac{M_i + M_{\{i+1\}}}{2} \times (H_{\{i+1\}} - H_i) \right|$$

Magnetic moment per formula unit (μ_B/f.u.): $\mu_B = \frac{(M_s \times M_{mol})}{(N_A \times \mu_B)}$

Exchange bias field: $H_{EB} = -\frac{(H_{c-} + H_{c+})}{2}$

Magnetic anisotropy constant (k erg/g): $K_A = \frac{(|M_s| \times |H_c|)}{(0.96 \times 1000)}$

Maximum energy product (kJ/m^3): $BH_{max} = \frac{\mu_0 \times M_s^2}{2000}$

ST4.2 Law of approach to saturation (LAS)

Full LAS model: $M(H) = M_s\left[1 - \left(\frac{a}{H}\right) - \left(\frac{b}{H^2}\right)\right] + kH$

Core–shell model: $M(H) = M_s\left[1 - \frac{1}{15} \cdot \frac{H_A^2}{\sqrt{H}\left(H^{\frac{3}{2}} + H_R^{\frac{3}{2}}\right)}\right] + kH$

Effective anisotropy constant (uniaxial assumption): $K_{eff} = \frac{M_s H_a}{2}$

ST5. Broadband permeability and dielectric processing

ST5.1 Complex permeability and permittivity: $\mu^* = \mu' - j\mu''$ and $\varepsilon^* = \varepsilon' - j\varepsilon''$

Loss tangents: $\tan\delta_m = \frac{\mu''}{\mu'}$ and ,$\tan_e = \tan\delta = \frac{\varepsilon''}{\varepsilon'}$

Quality factor: $Q = \frac{1}{\tan\delta_m}$

ST5.2 Modulus formalism

Electric modulus: $M^* = \frac{1}{\varepsilon^*} = M' + jM''$

Magnetic modulus: $M_\mu^* = \frac{1}{\mu^*}$

Relaxation time from peak frequency: $\square = \frac{1}{2\square f_{max}}$

ST5.3 Derived electromagnetic indicators

Matching impedance: $\frac{Z}{\square_0} = \sqrt{\frac{\square'}{\square'}}$

Complex magneto-dielectric modulus: $M^*_{MD} = \sqrt{\frac{\square^*}{\square^*}}$, M'_{MD} and M''_{MD} reported.

Effective wavelength (unit: m)

State the exact equation used in your script here. Common forms include: $\square_{eff} = \frac{c}{f\sqrt{\square'\square'}}$

ST6. Impedance spectroscopy and equivalent circuit fitting

ST6.1 Equivalent circuit: R_s -(R_g||CPE_g) - (R_{gb}||CPE_{gb})

CPE impedance: $Z_{CPE} = \frac{1}{Q\,(j\square)^a}$

where: Q is the pseudo-capacitance parameter, a ($0 < a \square 1$) is the dispersion exponent.

Extracted outputs: R_s, R_g, R_{gb}, a_g, a_{gb}, characteristic frequencies f_g and f_{gb} (as reported).

Supplementary Tables:

XRD Analysis

Table S1: Refinement quality factors and phase evolution of MZLF sample series

x	r_p	r_{wp}	r_{exp}	χ^2	Fd$\bar{3}$m				Pnma			
					Bragg R-factor	RF-factor	Fract (%)	Vol	Bragg R-factor	RF-factor	Fract (%)	Vol
**0.00**	15.03	10.70	1.04	1.062	3.980	5.260	100.00	605.19	x	x	x	x
**0.01**	20.60	14.70	13.30	1.222	3.890	4.140	100.00	602.95	x	x	x	x
**0.02**	19.00	12.20	11.30	1.171	3.570	3.620	98.99	602.63	125.0	47.2	1.01	241.596
**0.03**	17.20	11.90	11.70	1.093	8.470	6.970	98.93	602.58	85.1	46.9	1.07	241.28
**0.04**	20.00	14.30	11.80	1.462	3.730	4.300	98.27	600.47	36.6	28.7	1.73	241.291
**0.06**	16.02	11.40	11.40	0.999	5.780	4.680	95.94	601.96	39.5	31.8	4.06	242.148
**0.08**	18.10	12.70	10.90	1.337	4.860	4.870	96.19	602.01	35.7	37.4	3.81	242.529

Note: x: Substitutional or dopant concentration in the MZLF series. r_p: Profile residual factor (%). r_{wp}: Weighted profile residual factor (%). r_{exp}: Expected profile residual factor (%). χ^2: Goodness-of-fit indicator, calculated as $(r_{wp}/r_{exp})^2$. **Fd-3m**: Cubic phase structural parameters (spinel structure type). **Pnma**: Orthorhombic secondary phase structural parameters. **Bragg R-factor**: Indicator of the fit between calculated and observed integrated Bragg intensities. **RF-factor**: Structure factor residual component based on observed amplitude. **Fract (%)**: Relative weight fraction percentage of the respective crystalline phase. Vol: Unit cell volume measured in cubic angstroms ($Å^3$). x: Denotes that the secondary Pnma phase is absent or below the detection limit of conventional X-ray diffraction.

Table S2: Precise lattice parameter extraction via Nelson-Riley linear regression extrapolation

**x**	a_o	$\pm a_o$	**slope**	R^2	R^2_{adj}	**n**
**0.00**	8.4453	0.0056	-0.0040	0.4891	0.3869	7
**0.01**	8.4081	0.0114	-0.0135	0.6952	0.6444	8
**0.02**	8.4362	0.0054	-0.0020	0.2172	0.0607	7
**0.03**	8.4216	0.0129	-0.0012	0.0133	-0.1511	8

**0.04**	8.4182	0.0096	0.0007	0.0088	-0.1564	8
**0.06**	8.4365	0.0159	-0.0127	0.4355	0.3226	7
**0.08**	8.4362	0.0054	-0.0020	0.2172	0.0607	7

Note: **x**: Compositional dopant fraction; a_o: error-corrected lattice (Å); $\pm a_o$: Standard error of the lattice parameter (Å); **Slope**: Gradient of the linear fit (dimensionless); R^2: Coefficient of determination (dimensionless); R^2_{adj}: Adjusted coefficient of determination (dimensionless); **n**: Number of diffraction peaks.

Table S3: Crystallite size and microstrain parameters for MZLF compositions determined via Modified Scherrer and Williamson-Hall methods

x	n_{peaks}	$δ_{SM}$	$\pm δ_{SM}$	D_{WH}	$\pm D_{WH}$	**strain μ**	$\pm$ **strain μ**
**0.00**	9	36.05	3.93	38.50	8.95	441.35	518.24
**0.01**	7	27.63	4.25	28.54	8.08	522.02	900.82
**0.02**	9	36.40	3.35	48.72	11.75	1237.52	424.61
**0.03**	9	26.74	3.53	26.74	6.19	330.21	742.32
**0.04**	10	36.46	3.47	65.34	22.71	2071.30	468.34
**0.06**	10	27.64	2.79	26.98	4.82	117.73	583.09
**0.08**	10	38.69	2.29	54.72	10.22	1309.50	300.89

Note: n_{peaks} represents the number of diffraction peaks analyzed; $δ_{SM}$ and D_{WH} denote the average crystallite size (nm) calculated via the Modified Scherrer and Williamson-Hall methods, respectively; **strain μ** indicates the lattice microstrain.

Table S4: Dislocation density indicators derived from crystallite size and microstrain

x	$δ_{SM}$	$δ_{WH}$	$ρ_{WS}$	$\pm ρ_{WS}$	$□_{□}$	$\pm □_{□}$
**0.00**	76.96	67.46	6.65	7.96	0.05	0.13
**0.01**	130.97	122.75	10.61	18.55	0.08	0.26
**0.02**	75.46	42.12	14.73	6.18	0.43	0.29

**0.03**	139.82	139.90	7.16	16.19	0.03	0.14
**0.04**	75.22	23.42	18.39	7.62	1.20	0.54
**0.06**	130.85	137.41	2.53	12.55	0.00	0.04
**0.08**	66.82	33.39	13.88	4.11	0.48	0.22

Note: $\square_{SM}$ and $\square_{WH}$ are size-based proxies, $\square_{WS}$ is Williamson-Smallman dislocation density, $\square_{\square}$ is a strain-only proxy. All values are reported in femto-lines m^{-2}.

Table S5: Hopping lengths (L_A, L_B), bond lengths, and calculated site radii

x	L_A	L_B	d_{AX}	d_{BX}	d_{AXE}	d_{BXE}	d_{BEU}	r_A	r_B
**0.00**	3.6569	2.9859	5.2943	2.0619	6.1134	5.8266	2.9876	0.5162	0.6607
**0.01**	3.6408	2.9727	5.2710	2.0528	6.0864	5.8010	2.9744	0.5078	0.6516
**0.02**	3.6530	2.9826	5.2886	2.0597	6.1068	5.8204	2.9844	0.5142	0.6584
**0.03**	3.6467	2.9775	5.2795	2.0561	6.0962	5.8103	2.9792	0.5108	0.6549
**0.04**	3.6452	2.9763	5.2773	2.0553	6.0938	5.8079	2.9780	0.5101	0.6540
**0.06**	3.6509	2.9810	5.2857	2.0585	6.1034	5.8171	2.9827	0.5131	0.6573
**0.08**	3.6530	2.9826	5.2886	2.0597	6.1068	5.8204	2.9844	0.5142	0.6584

Note: Hopping length L_a and L_b are reported in Å. Bond lengths d_{AXE}, d_{BXE}, and $d_{BEU}$are reported in Å. Site ionic radius r_A and r_B are reported in Å.

Table S6: Calculated interatomic bond distances (b-s) for the spinel structure

x	b (Å)	c (Å)	d (Å)	e (Å)	f (Å)	p (Å)	q (Å)	r (Å)	s (Å)
**0.00**	2.985864	3.501236	3.656922	5.485383	5.171669	2.060653	1.916227	3.669296	3.686178
**0.01**	2.972712	3.485814	3.640814	5.461221	5.148889	2.051576	1.907787	3.653133	3.669941

**0.02**	2.982647	3.497464	3.652982	5.479473	5.166096	2.058433	1.914162	3.665342	3.682206
**0.03**	2.977485	3.491411	3.646660	5.469990	5.157156	2.054870	1.910850	3.658999	3.675833
**0.04**	2.976283	3.490001	3.645188	5.467781	5.155074	2.054041	1.910078	3.657521	3.674349
**0.06**	2.982753	3.497588	3.653112	5.479667	5.166280	2.058506	1.914231	3.665472	3.682337
**0.08**	2.982647	3.497464	3.652982	5.479473	5.166096	2.058433	1.914162	3.665342	3.682206

Note: All bond distances (b, c, d, e, f, p, q, r, s) are reported in Å.

Table S7: Calculated bond angles ($\square_1 - \square_5$) for the spinel ferrite structure

**x**	$\square_1$ (°)	$\square_2$ (°)	$\square_3$ (°)	$\square_4$ (°)	$\square_5$ (°)
**0.00**	123.3400	144.9469	92.8537	125.9180	112.8241
**0.01**	123.3400	144.9469	92.8537	125.9180	112.8241
**0.02**	123.3400	144.9469	92.8537	125.9180	112.8241
**0.03**	123.3400	144.9469	92.8537	125.9180	112.8241
**0.04**	123.3400	144.9469	92.8537	125.9180	112.8241
**0.06**	123.3400	144.9469	92.8537	125.9180	112.8241
**0.08**	123.3400	144.9469	92.8537	125.9180	112.8241

Note: All angles ($\square_1 - \square_5$) are reported in degrees (°).

SEM segmentation and microstructure metrics

Table S8: SEM grain size statistics from Feret diameter analysis with log-normal fitting

x	D_g	$\pm D_g$	R^2_{adj}	N
0.00	1.872	0.127	0.942	107
0.01	1.883	0.031	0.971	198
0.02	1.408	0.111	0.740	140
0.03	1.706	0.179	0.784	148
0.04	1.560	0.066	0.847	184

**0.06**	1.598	0.081	0.858	153
**0.08**	1.072	0.020	0.994	233

Note: Grain size D_g reported in micrometer (μm), goodness of fit R^2_{adj} is dimensionless, grain count is **N**.

Table S9: SEM image-derived porosity and pore-network descriptors using method-adaptive segmentation

x	**Method**	ϕ	N_P	D_{eq}	$\tilde{D}_{eq}$	T_{loc}	$\tilde{T}_{loc}$	P_V	P_H	χ
**0.00**	sauvola	11.98	458	1.54	1.2	0.4	0.36	False	False	447
**0.01**	sauvola	20.22	1320	1.0	0.63	0.33	0.25	False	False	1249
**0.02**	watershed	4.15	501	0.71	0.47	0.52	0.36	False	False	499
**0.03**	manual	8.02	131	1.71	1.02	1.59	1.05	False	False	122
**0.04**	watershed	10.75	413	0.96	0.44	1.03	0.72	False	False	408
**0.06**	manual	5.36	263	1.01	0.56	0.82	0.57	False	False	257
**0.08**	manual	5.53	697	0.65	0.42	0.6	0.4	False	False	695

Note: Here, **Method**: SEM pore-segmentation route (sauvola, watershed, or manual, categorical); Porosity (ϕ): 2D pore area fraction from SEM (%); N_P: Number of segmented pores (unitless); D_{eq} and $\tilde{D}_{eq}$: Mean and median equivalent pore diameter (μm); T_{loc} and $\tilde{T}_{loc}$: Mean and median local pore thickness (μm); P_V and P_H: Vertical and horizontal percolation flags (percolates top to bottom, percolates left to right, True/False); χ: Euler characteristic of the pore mask (unitless).

Table S10: EDS normalized elemental composition (semi-quantitative) for Mn-Zn-La ferrites

**x**		**0.00**	**0.01**	**0.02**	**0.03**	**0.04**	**0.06**	**0.08**
**Mn (wt.%)**	Obs.	7.01	4.28	3.92	22.10	5.80	20.30	4.39
	Theo.	4.60	4.58	4.57	4.55	4.53	4.50	4.47

	Rel.	52.52	-6.66	-14.18	385.58	27.83	350.70	-1.90
**Zn (wt.%)**	Obs.	20.58	20.51	22.77	13.72	16.63	15.77	16.73
	Theo.	21.89	21.81	21.74	21.66	21.59	21.44	21.29
	Rel.	-5.96	-5.98	4.77	-36.66	-22.98	-26.44	-21.42
**La (wt.%)**	Obs.	0.00	2.13	1.26	0.70	1.25	2.12	5.26
	Theo.	0.00	0.58	1.15	1.73	2.29	3.42	4.52
	Rel.	0.00	267.63	9.24	-59.72	-45.32	-37.85	16.36
**Fe (wt.%)**	Obs.	45.63	46.40	45.45	36.99	49.91	35.58	47.56
	Theo.	46.74	46.34	45.95	45.56	45.17	44.41	43.65
	Rel.	-2.38	0.13	-1.08	-18.82	10.49	-19.89	8.95
**O (wt.%)**	Obs.	26.78	26.69	26.59	26.50	26.41	26.23	26.05
	Theo.	26.78	26.69	26.59	26.50	26.41	26.23	26.05
	Rel.	0.00	0.00	0.00	0.00	0.00	0.00	0.00

Note: x represents the stoichiometric substitution level. Theoretical values (Theo.) are calculated based on the chemical formula $Mn_{0.2}Zn_{0.8}La_xFe_{2-x}O_4$. Observed values (Obs.) were obtained via EDS analysis and normalized to 100 wt.%. The relative error (Rel.) is calculated as:

$$\text{Rel. } (\%) = \frac{W_{obs} - W_{theo}}{W_{theo}} \times 100$$

Negative values indicate an elemental deficiency relative to the theoretical stoichiometric ratio.

Table S11: EDS acquisition conditions used for elemental analysis (magnification and count rate)

x	Magnification	Count Rate (cps)
0.00	×40	5343
0.01	×45	3623
0.02	×80	5565
0.03	×4000	1648
0.04	×3000	1859
0.06	×3000	1518
0.08	×10	3330

Note: Magnification is reported as × (unitless). Count rate is reported in counts per second (cps).

Supplementary discussion on VSM: branch symmetry, derived VSM metrics

The M-H loops were analyzed separately for the negative and positive branches to check symmetry and to reduce bias from small centering or drift effects (Fig. S8). For each composition, M_{s+} and M_{s-} represent the positive and negative saturation values obtained from the two branches. Their absolute magnitudes are very similar, with M_{s+}/M_{s-} ratios close to unity (about 0.99–1.03) (Table S12). This indicates that the loops are essentially symmetric in saturation and that the samples do not show a strong vertical shift. In the same way, H_{c-} and H_{c+} are the coercivities extracted from the negative and positive M = 0 crossings, while M_{r+} and M_{r-} are the remanent magnetizations extracted at H = 0 from each branch using linear interpolation. Small differences between the positive and negative branch values are expected in soft ferrites due to minor instrumental offsets, smoothing and interpolation near the crossing points, and microstructural pinning asymmetry. Therefore, the main text uses $H_{c\ avg}$ as a robust single coercivity descriptor, while the branch-resolved values are retained here for completeness and quality checking.

The hysteresis loop area, reported in emu·Oe, was calculated by numerical integration of $|M\ dH|$ over the full cycle and is shown composition-wise. This area serves as a comparative indicator of quasi-static hysteresis loss within this consistent measurement window. It scales with both loop height (magnetization) and loop width (coercivity). The parameter $H_{c\ field}$ is reported as the coercive-field value used internally in the post-processing workflow for subsequent derived calculations. In this dataset, Hc field is consistent with Hc avg unless otherwise stated, so it can be treated as the effective coercivity adopted for anisotropy- and energy-related estimates.

The maximum energy product, BH_{max} provides a useful internal comparison of the loop "strength" within the same analysis assumptions, but it should not be interpreted as a permanent-magnet performance metric because true BH_{max} is normally extracted from the second quadrant B-H curve and depends on the measured density and demagnetization. The magnetic moment per formula unit closely follows the M_s trend because it is proportional to M_s while M_{mol} varies only slightly with x. The anisotropy-related parameter K_A provides a convenient comparative indicator that combines magnetization and coercive field into a single anisotropy-linked scale, but it remains semi-quantitative because it is sensitive to the selected H_c value and to extrinsic factors such as porosity and microcracking that influence domain-wall pinning. For an ideally symmetric soft ferrite loop, H_{EB} should be near zero. Therefore, small non-zero HEB values in this series are interpreted mainly as indicators of minor loop centering offsets or weak branch asymmetry, rather than evidence of strong exchange-bias coupling.

Finally, the differential susceptibility dM/dH (emu/Oe) was calculated as a slope descriptor over the full measured field range (0 to 15 kOe) and is compared in Fig. S8. This parameter provides a compact measure of how quickly magnetization changes with field within the same experimental window. However, because it depends on the chosen field span and on any smoothing applied to the M–H data, it is used here only for internal trend checking and not as a primary performance metric.

The anisotropy field H_a was also estimated using the single-point detection (SPD) approach, and the resulting Ha values are summarized in Fig. S9 provides a rapid, fitting-light estimate of the field scale where the magnetization

approaches saturation, so it is useful for comparing compositions without relying on a full model fit. In this series, SPD-derived Ha follows the same composition-dependent trend observed from the high-field analysis, supporting that La substitution modifies the effective anisotropy and the approach-to-saturation behavior; however, Ha values should be treated as comparative indicators because they depend on the chosen SPD criterion and the experimental field window (±15 kOe).

VSM Analysis tables:

Table S12: Static magnetic parameters from M-H loop analysis

x	0.00	0.01	0.02	0.03	0.04	0.06	0.08
M_{s+} (emu/g)	6.53	18.93	15.61	16.50	33.96	15.53	11.59
M_{s-} (emu/g)	-6.35	-18.40	-15.80	-16.04	-34.03	-15.56	-11.51
M_{r+} (emu/g)	0.49	3.44	2.84	1.79	5.25	2.59	1.62
M_{r-} (emu/g)	-0.37	-3.37	-3.03	-1.97	-5.34	-2.53	-1.58
H_{c+} (Oe)	-180.73	-64.92	-66.36	-43.45	-62.43	-67.08	-58.26
H_{c-} (Oe)	139.09	63.72	70.47	51.83	63.41	65.41	56.72
M_{s+}/M_{s-}	1.03	1.03	0.99	1.03	1.00	1.00	1.01
$H_{c\ avg}$ (Oe)	159.91	64.32	68.42	47.64	62.92	66.25	57.49
Loop Area	1555.22	3734.74	2224.30	5983.40	3162.30	1277.27	2121.29
$H_{c\ field}$ (Oe)	159.91	64.32	68.42	47.64	62.92	66.25	57.49
$\square_B$/f.u.	0.28	0.81	0.67	0.71	1.47	0.68	0.51
K_A (k erg/g)	1228.90	1279.97	1079.43	746.98	2208.23	1085.13	703.45
BH_{max} kJ/m³	0.67	5.63	3.83	4.28	18.11	3.79	2.11
H_{EB}(Oe)	20.82	0.60	-2.05	-4.19	-0.49	0.83	0.77
dM/dH (emu/Oe)	0.0004	0.0012	0.0010	0.0010	0.0022	0.0010	0.0007

Table S13: Law of approach to saturation (LAS) fitting parameters and effective anisotropy constant

x	VSM M_s	Full M_s	CS M_s	Full a	Full b	CS H_A	CS H_R	Better Model	$K_{eff} \times 10^5$
0.00	6.53	4.69	1.81	6868.44	-24615252.02	9121.32	17760.10	Full LAS	0.82
0.01	18.93	21.11	16.10	2763.98	-9708531.33	9030.12	19777.61	Full LAS	7.27
0.02	15.61	15.17	14.13	1083.15	-2917099.66	10521.39	12699.41	Full LAS	7.43
0.03	16.50	19.53	16.55	3029.31	-8728070.44	25834.05	19695.91	Full LAS	21.38

**0.04**	33.96	36.18	32.38	1428.33	-4648957.51	11802.14	20475.65	Full LAS	19.11
**0.06**	15.53	12.28	12.66	0.00	-1216588.61	0.62	53999.90	Full LAS	0.00
**0.08**	11.59	10.65	9.54	1536.91	-4853124.66	14727.96	21935.58	Full LAS	7.03

Note. Full M_s: saturation magnetization from the full LAS model (emu/g); Full a: law of approach parameter related to structural defects; Full b and CS H_A: parameter related to magnetocrystalline anisotropy; CS M_s, CS H_A, CS H_R: fitting parameters for the Core-Shell (CS) model; Better Model: statistical determination of the superior fitting approach; R^2 Difference: variance between model correlations; VSM M_s: experimental saturation magnetization from VSM; K_{eff}: effective anisotropy constant. The "Full LAS" model was identified as the best fit for all concentrations.

Frequency dependent magnetic properties

Table S14: Real part of magnetic permeability (μ')

Frequency	0.00	0.01	0.02	0.03	0.04	0.06	0.08
**100Hz**	12.45	13.73	13.87	16.58	12.93	10.76	10.86
**1kHz**	14.36	13.45	13.55	16.91	14.42	12.35	11.77
**10kHz**	13.65	13.69	13.68	16.94	14.45	12.68	12.46
**100kHz**	13.41	13.54	13.45	16.67	14.34	12.53	12.22
**1MHz**	12.92	13.07	12.97	15.99	13.89	12.13	11.72
**10MHz**	12.41	12.54	12.41	15.29	13.34	11.65	11.20
**50MHz**	12.23	12.34	12.23	15.03	13.18	11.47	11.01
**100MHz**	12.12	12.26	12.18	14.87	13.21	11.39	10.94
**120MHz**	12.09	12.24	12.17	14.82	13.24	11.35	10.92

Table S15: Imaginary part of magnetic permeability (μ'')

Frequency	0.00	0.01	0.02	0.03	0.04	0.06	0.08
**100Hz**	3053.67	4436.95	4064.33	5920.52	4412.02	3010.80	3480.99

**1kHz**	473.89	489.46	477.41	534.48	432.57	370.43	359.02
**10kHz**	44.34	47.81	48.15	56.04	43.27	38.74	39.09
**100kHz**	4.57	4.88	4.88	5.69	4.36	3.97	4.03
**1MHz**	0.82	0.87	0.87	1.03	0.76	0.73	0.76
**10MHz**	0.23	0.27	0.27	0.32	0.23	0.22	0.24
**50MHz**	0.09	0.13	0.12	0.14	0.09	0.09	0.11
**100MHz**	0.08	0.12	0.09	0.12	0.06	0.07	0.08
**120MHz**	0.08	0.11	0.08	0.11	0.05	0.04	0.05

Table S16: Magnetic loss tangent ($\tan \delta_m$)

**Frequency**	**0.00**	**0.01**	**0.02**	**0.03**	**0.04**	**0.06**	**0.08**
**100Hz**	245.248	323.103	292.934	357.165	341.137	279.831	320.522
**1kHz**	32.991	36.400	35.226	31.601	30.005	29.994	30.500
**10kHz**	3.249	3.492	3.519	3.308	2.995	3.055	3.138
**100kHz**	0.341	0.361	0.362	0.341	0.304	0.317	0.330
**1MHz**	0.063	0.065	0.066	0.063	0.054	0.059	0.063
**10MHz**	0.018	0.021	0.022	0.021	0.017	0.019	0.022
**50MHz**	0.007	0.011	0.010	0.010	0.007	0.008	0.010
**100MHz**	0.007	0.010	0.007	0.008	0.005	0.006	0.007
**120MHz**	0.007	0.009	0.007	0.007	0.004	0.004	0.005

Table S17: Magnetic quality factor (Q)

**Frequency**	**0.00**	**0.01**	**0.02**	**0.03**	**0.04**	**0.06**	**0.08**
**100Hz**	0.00	0.00	0.00	0.00	0.00	0.00	0.00
**1kHz**	0.03	0.03	0.03	0.03	0.03	0.03	0.03
**10kHz**	0.31	0.29	0.28	0.30	0.33	0.33	0.32
**100kHz**	2.94	2.77	2.76	2.93	3.29	3.16	3.03
**1MHz**	16.00	15.35	15.17	15.88	18.54	17.00	15.78

10MHz	54.70	46.72	46.14	47.50	58.14	51.90	45.71
50MHz	139.81	93.75	99.26	103.72	144.06	121.39	101.61
100MHz	153.01	102.82	136.74	120.53	213.03	166.12	135.17
120MHz	151.22	106.87	147.42	136.61	242.23	259.65	199.48

Table S18: Real part of magnetic modulus ($M'_{\square}$)

Frequency	0.00	0.01	0.02	0.03	0.04	0.06	0.08
100Hz	0.0000	0.0000	0.0000	0.0000	0.0000	0.0000	0.0000
1kHz	0.0001	0.0001	0.0001	0.0001	0.0001	0.0001	0.0001
10kHz	0.0063	0.0055	0.0055	0.0049	0.0069	0.0076	0.0074
100kHz	0.0668	0.0654	0.0657	0.0537	0.0638	0.0725	0.0738
1MHz	0.0771	0.0762	0.0768	0.0623	0.0718	0.0821	0.0849
10MHz	0.0806	0.0797	0.0805	0.0654	0.0750	0.0858	0.0893
50MHz	0.0818	0.0810	0.0818	0.0665	0.0759	0.0872	0.0908
100MHz	0.0825	0.0815	0.0821	0.0672	0.0757	0.0878	0.0914
120MHz	0.0827	0.0817	0.0822	0.0675	0.0755	0.0881	0.0915

Table S19: Imaginary part of magnetic modulus ($M''_{μ}$)

Frequency	0.00	0.01	0.02	0.03	0.04	0.06	0.08
100Hz	0.0003	0.0002	0.0002	0.0002	0.0002	0.0003	0.0003
1kHz	0.0021	0.0020	0.0021	0.0019	0.0023	0.0027	0.0028
10kHz	0.0206	0.0193	0.0192	0.0163	0.0208	0.0233	0.0232
100kHz	0.0228	0.0236	0.0238	0.0183	0.0194	0.0230	0.0243
1MHz	0.0049	0.0050	0.0051	0.0040	0.0039	0.0049	0.0055
10MHz	0.0015	0.0017	0.0017	0.0014	0.0013	0.0017	0.0020
50MHz	0.0006	0.0009	0.0008	0.0006	0.0005	0.0007	0.0009
100MHz	0.0005	0.0008	0.0006	0.0006	0.0004	0.0005	0.0007
120MHz	0.0005	0.0008	0.0006	0.0005	0.0003	0.0003	0.0005

Table S20: Magnetic relaxation parameters

x	f_{max}(Hz)	τ (s)
0.00	36279.0	4.38×10^{-6}
0.01	35028.3	4.54×10^{-6}
0.02	35028.3	4.54×10^{-6}
0.03	36279.0	4.38×10^{-6}
0.04	33820.7	4.70×10^{-6}

0.06	31529.1	5.04×10^{-6}
0.08	32654.8	4.87×10^{-6}

Frequency Dependent Dielectric Properties

Table S21: Real part of dielectric constant (ε')

Frequency	0.00	0.01	0.02	0.03	0.04	0.06	0.08
100Hz	2372.52	6498.85	7735.17	50599.24	20659.49	31134.19	42076.90
1kHz	719.34	923.45	1253.87	10389.64	2516.53	4527.70	6600.63
10kHz	193.62	142.75	162.14	1139.63	249.02	445.42	652.03
100kHz	117.22	47.65	43.61	138.36	54.93	65.34	93.02
1MHz	74.35	24.97	23.82	42.18	25.26	25.73	32.22
10MHz	37.81	18.06	17.60	23.81	16.24	17.63	20.73
50MHz	24.20	15.47	15.11	18.87	12.84	15.21	16.81
100MHz	20.14	13.95	13.60	16.64	11.19	13.84	14.92
120MHz	19.11	13.42	13.02	15.99	10.68	13.37	14.30

Table S22: Imaginary part of dielectric constant (ε'')

Frequency	0.00	0.01	0.02	0.03	0.04	0.06	0.08
100Hz	4315.90	41585.61	54239.84	574554.42	264069.59	431093.30	582125.44
1kHz	1193.46	3317.11	5782.90	104210.17	16868.43	39706.60	62752.08
10kHz	152.69	207.92	302.91	5103.56	581.79	1494.26	2442.18
100kHz	62.09	37.28	33.78	222.54	54.23	79.04	129.12
1MHz	50.82	10.63	9.62	30.54	13.81	13.30	19.80
10MHz	22.94	4.04	3.76	8.65	5.24	4.20	6.63
50MHz	10.37	2.30	2.22	4.63	2.90	2.17	3.48
100MHz	7.38	1.80	1.81	3.69	2.25	1.66	2.66
120MHz	6.77	1.73	1.63	3.43	2.00	1.59	2.40

Table S23: Dielectric loss tangent ($\tan\delta$)

Frequency	0.00	0.01	0.02	0.03	0.04	0.06	0.08
100Hz	1.82	6.40	7.01	11.36	12.78	13.85	13.83
1kHz	1.19	2.19	3.00	7.10	3.89	5.48	6.09
10kHz	0.53	0.77	0.76	1.56	0.97	1.17	1.35
100kHz	0.68	0.29	0.28	0.48	0.40	0.33	0.40
1MHz	0.35	0.13	0.12	0.21	0.19	0.12	0.17
10MHz	0.35	0.13	0.12	0.21	0.19	0.12	0.17
50MHz	0.35	0.13	0.12	0.21	0.19	0.12	0.17
100MHz	0.35	0.13	0.12	0.21	0.19	0.12	0.17
120MHz	0.35	0.13	0.12	0.21	0.19	0.12	0.17

Table S24: Real part of electric modulus (M')

Frequency	0.00	0.01	0.02	0.03	0.04	0.06	0.08
100Hz	0.0002	0.0000	0.0000	0.0000	0.0000	0.0000	0.0000
1kHz	0.0006	0.0003	0.0002	0.0000	0.0001	0.0000	0.0000
10kHz	0.0025	0.0033	0.0026	0.0002	0.0015	0.0006	0.0004
100kHz	0.0035	0.0102	0.0111	0.0032	0.0091	0.0075	0.0051
1MHz	0.0063	0.0144	0.0146	0.0113	0.0167	0.0159	0.0138
10MHz	0.0117	0.0118	0.0116	0.0135	0.0180	0.0128	0.0140
50MHz	0.0150	0.0094	0.0095	0.0123	0.0167	0.0092	0.0118
100MHz	0.0160	0.0091	0.0096	0.0127	0.0172	0.0085	0.0116
120MHz	0.0165	0.0094	0.0094	0.0128	0.0169	0.0087	0.0114

Table S25: Imaginary part of electric modulus (M'')

Frequency	0.00	0.01	0.02	0.03	0.04	0.06	0.08
100Hz	0.27	0.03	0.03	0.00	0.00	0.00	0.00
1kHz	0.92	0.35	0.23	0.01	0.08	0.02	0.02
10kHz	3.74	4.13	3.63	0.26	1.93	0.55	0.51
100kHz	5.26	12.88	15.70	4.52	12.06	6.70	6.82
1MHz	9.34	18.26	20.62	15.71	22.09	14.14	18.53
10MHz	17.49	14.93	16.43	18.81	23.84	11.40	18.74
50MHz	22.31	11.89	13.49	17.11	22.15	8.20	15.81

**100MHz**	23.90	11.52	13.64	17.73	22.85	7.61	15.49
**120MHz**	24.55	11.95	13.36	17.89	22.42	7.80	15.29

Table S26: Dielectric relaxation parameters

x	f_{max}(Hz)	τ(s)
**0.00**	120000000.0	1.32×10^{09}
**0.01**	981394.0	1.62×10^{07}
**0.02**	947561.0	1.67×10^{07}
**0.03**	4280000.0	3.71×10^{08}
**0.04**	5870000.0	2.71×10^{08}
**0.06**	1440000.0	1.10×10^{07}
**0.08**	2530000.0	6.29×10^{08}

Table S27: Equivalent-circuit fitting parameters from Nyquist plots

x	R_s	R_g	R_{gb}	$α_g$	$α_{gb}$	$C_{g,eff}$	$C_{gb,eff}$	f_g	f_{gb}	**cost**
**0.00**	33.3186	717.7062	2754.4348	0.7780	0.7770	146.2670	24.5995	15.1610	23.4888	0.0334
**0.01**	25.4932	2143.0797	1081.6958	0.8080	0.7061	87.5541	33.8674	8.4821	43.4443	0.0423
**0.02**	24.8929	2748.5171	1336.0380	0.8051	0.7047	83.4839	24.2143	6.9362	49.1959	0.0367

**0.03**	6.3768	1676.6256	955.6642	0.8009	0.6728	92.7187	51.0532	10.2380	32.6206	0.0466
**0.04**	37.5364	2557.0520	240.7362	0.7417	1.0000	106.8242	72.3132	5.8265	91.4242	0.1410
**0.06**	17.7024	3606.2645	1304.8017	0.7725	0.6391	105.8854	13.3395	4.1680	91.4403	0.0529
**0.08**	33.5666	6824.0159	1015.7070	0.7483	1.0000	116.0374	47.2156	2.0099	33.1869	0.0479

Note. R_s: series or bulk resistance (Ω); R_g: grain resistance (kΩ); R_{gb}: grain boundary resistance (kΩ); $α_g$ and $α_{gb}$: depression factors (constant phase element exponents) for grain and grain boundary, respectively; $C_{g,eff}$ and $C_{gb,eff}$: effective capacitance (pF and nF respectevely); f_g and f_{gb} : relaxation frequencies (kHz and Hz respectively). Values were obtained by fitting Nyquist plots using a standard $(R_gCPE_g)(R_{gb}CPE_{gb})$ equivalent circuit.

Table S28: Normalized impedance magnitude ratio $(Z/□_{o})$

**Frequency**	**0.00**	**0.01**	**0.02**	**0.03**	**0.04**	**0.06**	**0.08**
**100Hz**	0.0724	0.0460	0.0424	0.0181	0.0250	0.0186	0.0161
**1kHz**	0.1413	0.1207	0.1040	0.0403	0.0757	0.0522	0.0422
**10kHz**	0.2655	0.3097	0.2905	0.1219	0.2409	0.1687	0.1382
**100kHz**	0.3383	0.5331	0.5554	0.3471	0.5110	0.4379	0.3624
**1MHz**	0.4169	0.7234	0.7379	0.6157	0.7416	0.6867	0.6032
**10MHz**	0.5728	0.8331	0.8399	0.8014	0.9061	0.8128	0.7350
**50MHz**	0.7109	0.8931	0.8998	0.8923	1.0130	0.8685	0.8092
**100MHz**	0.7758	0.9375	0.9463	0.9454	1.0864	0.9070	0.8560

**120MHz**	0.7953	0.9550	0.9667	0.9630	1.1138	0.9214	0.8741

Table S29: Effective wavelength ($\square_{eff}$)

Frequency	0.00	0.01	0.02	0.03	0.04	0.06	0.08
**100Hz**	17442.47	9930.26	9016.98	3273.43	5408.38	5085.06	4371.51
**1kHz**	2911.61	2655.98	2270.39	706.04	1553.85	1227.10	1045.04
**10kHz**	588.71	684.50	642.46	217.79	504.54	402.65	335.78
**100kHz**	75.35	117.62	123.33	62.21	106.45	104.42	88.62
**1MHz**	9.86	16.91	17.38	11.76	16.31	17.29	15.72
**10MHz**	1.39	2.00	2.04	1.58	2.05	2.10	1.98
**50MHz**	0.35	0.43	0.44	0.36	0.46	0.45	0.44
**100MHz**	0.19	0.23	0.23	0.19	0.24	0.24	0.23
**120MHz**	0.17	0.20	0.20	0.16	0.21	0.21	0.20

Supplementary Figs:

1. Crystal structure of MZLF

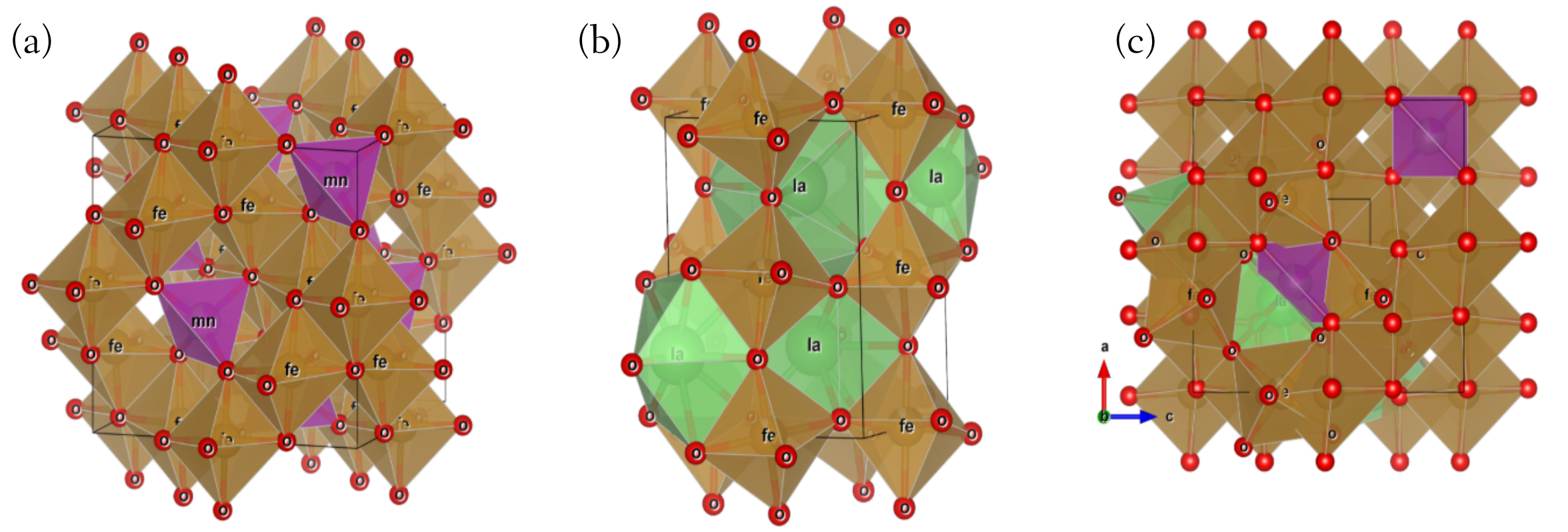

Fig. S1 Crystal structure representations of the synthesized materials. **(a)** Cubic spinel structure of MZLF crystallizing in the $Fd\bar{3}m$ space group, highlighting the octahedral networks (brown polyhedra) and tetrahedral sites (purple polyhedra). **(b)** Orthorhombic perovskite structure of $LaFeO_3$ crystallizing in the Pnma space group, showing octahedra (brown) coordinated with La sites (green polyhedra). **(c)** Structural model of the dual-phase composite viewed along the crystallographic axes, demonstrating the coexistence and interfacial distribution of both spinel (purple/brown) and perovskite (green/brown) phases within the lattice matrix. Oxygen atoms are represented as red spheres labeled 'O'.

2. Detailed sample wise Rietveld refinement plot

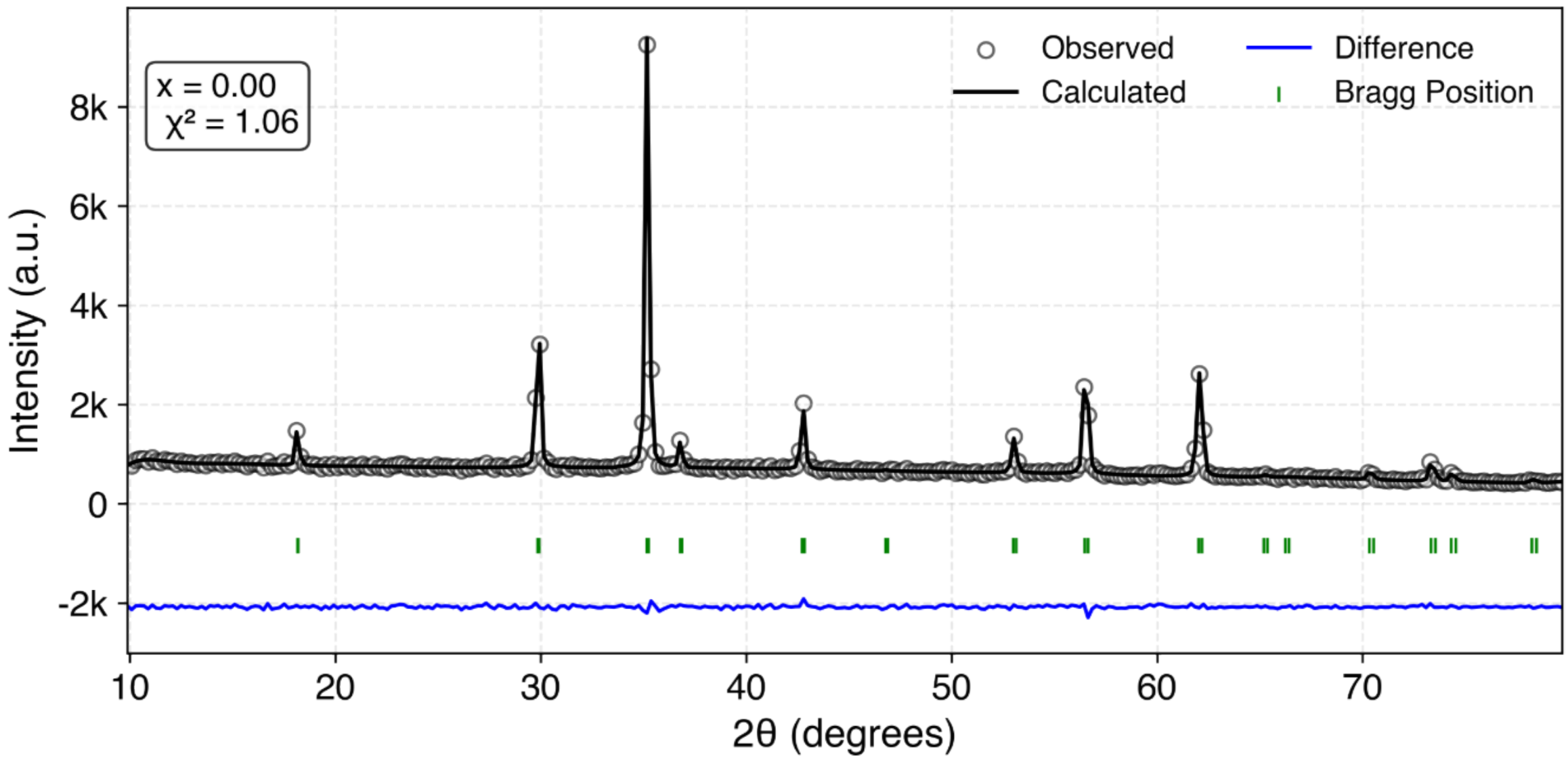

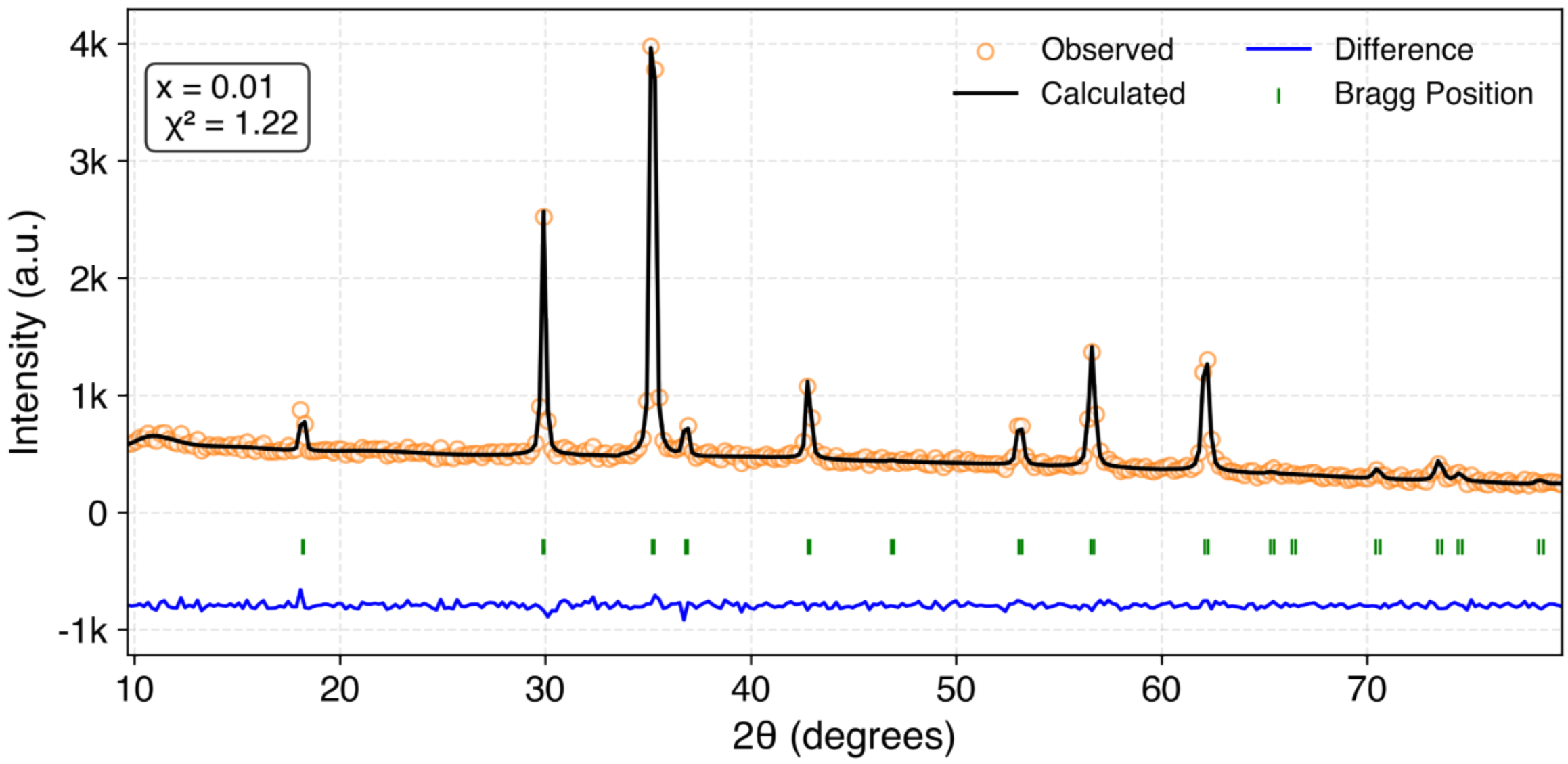

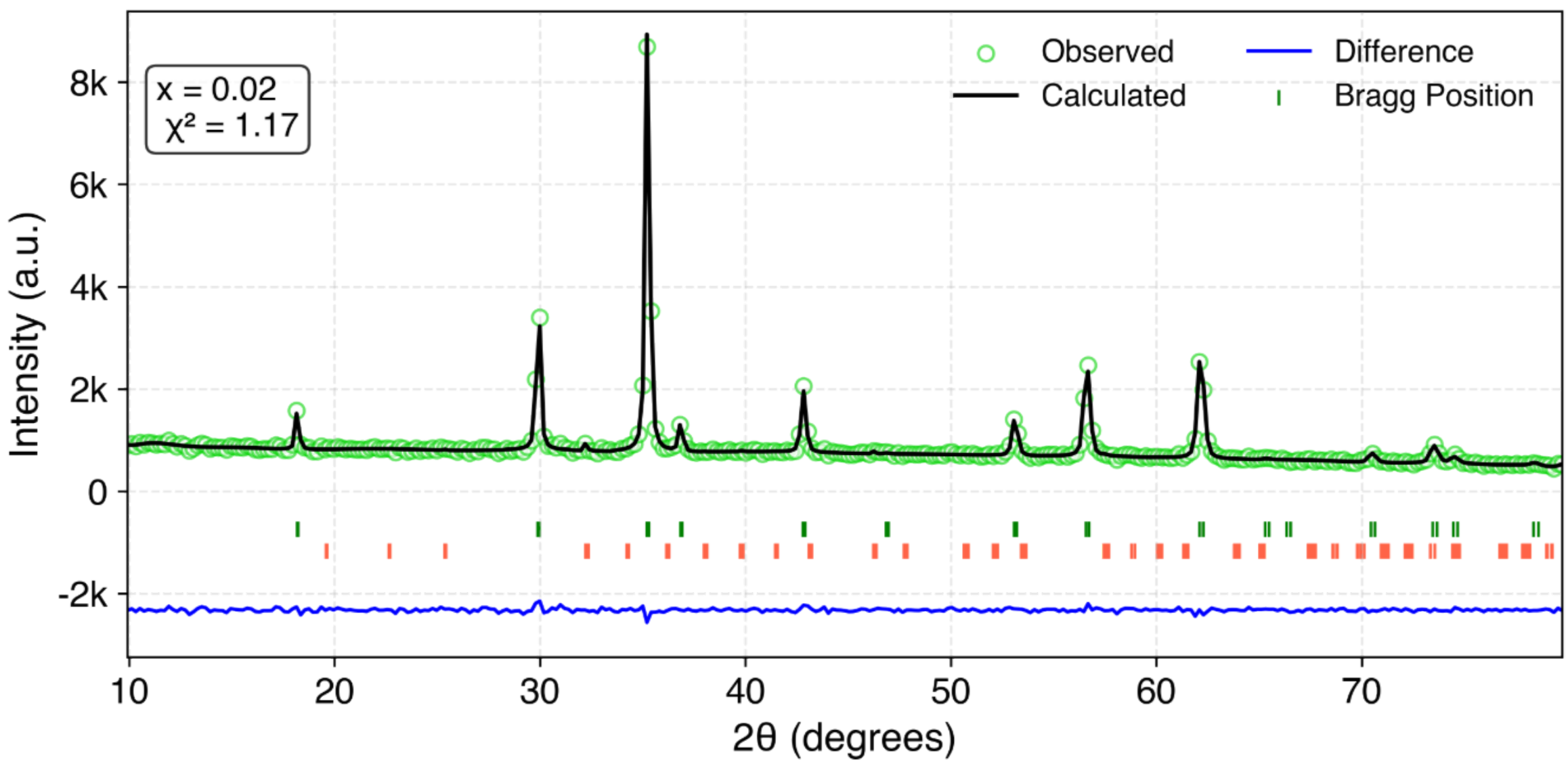

Fig S2 continued

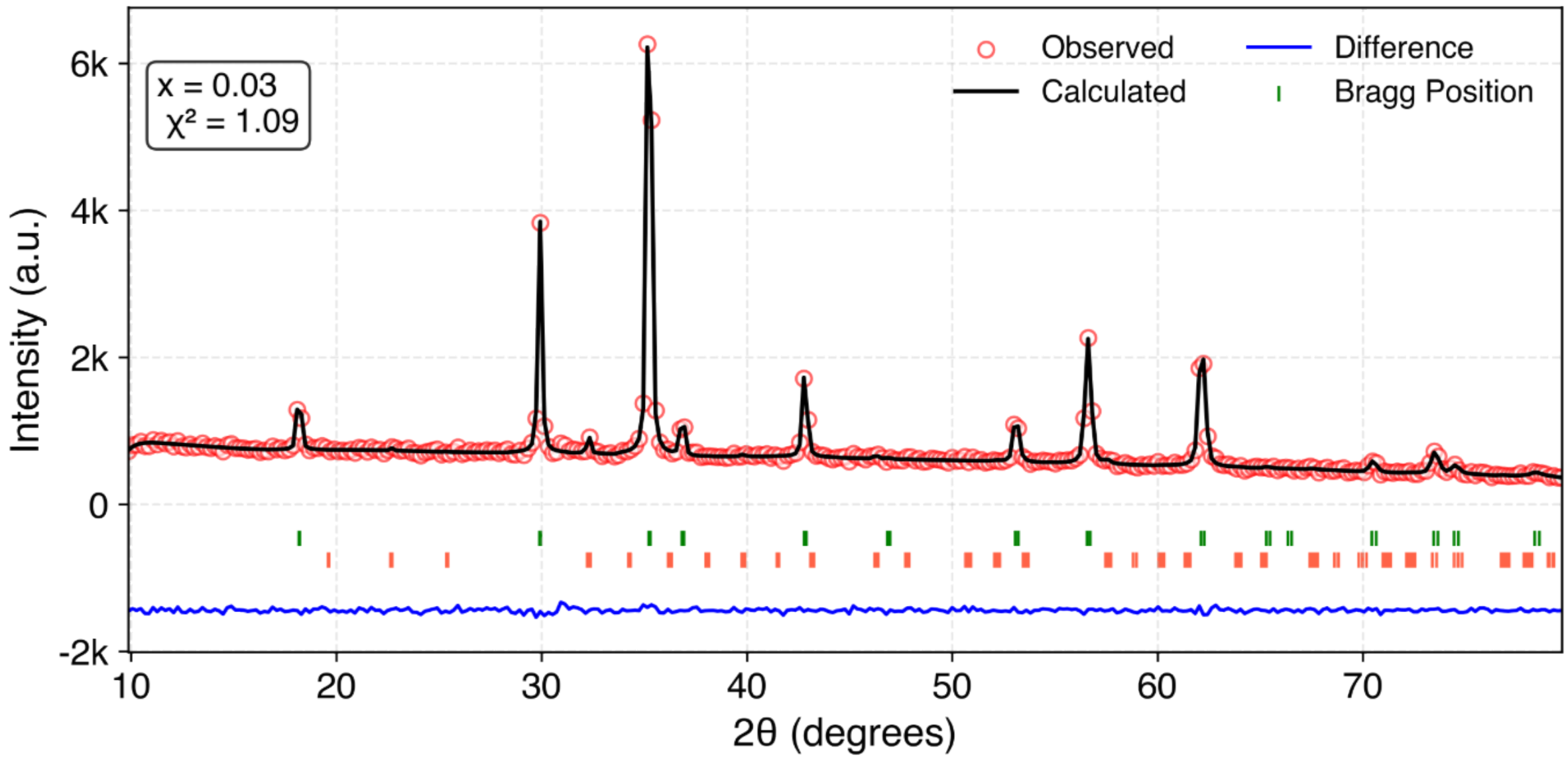

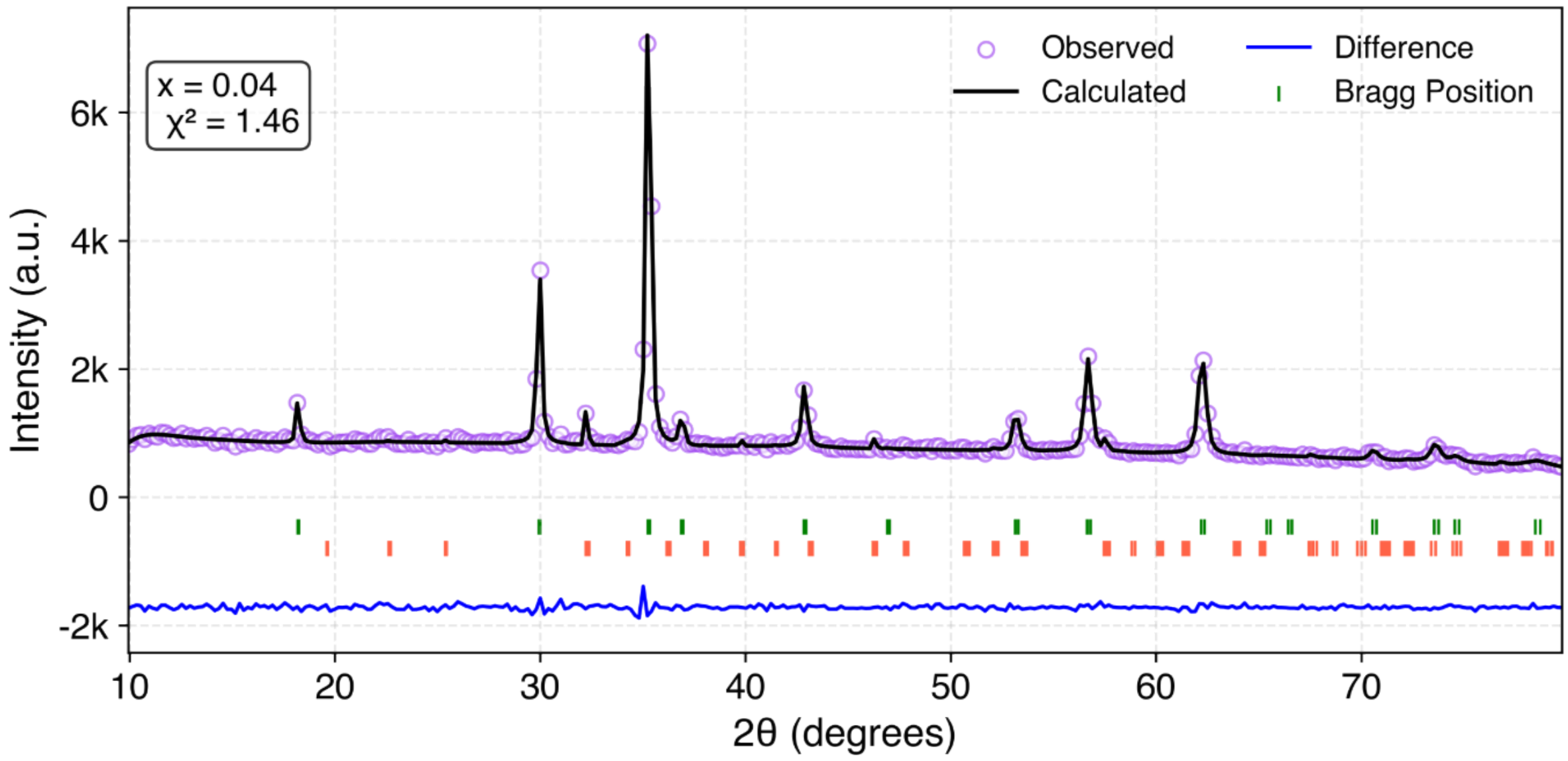

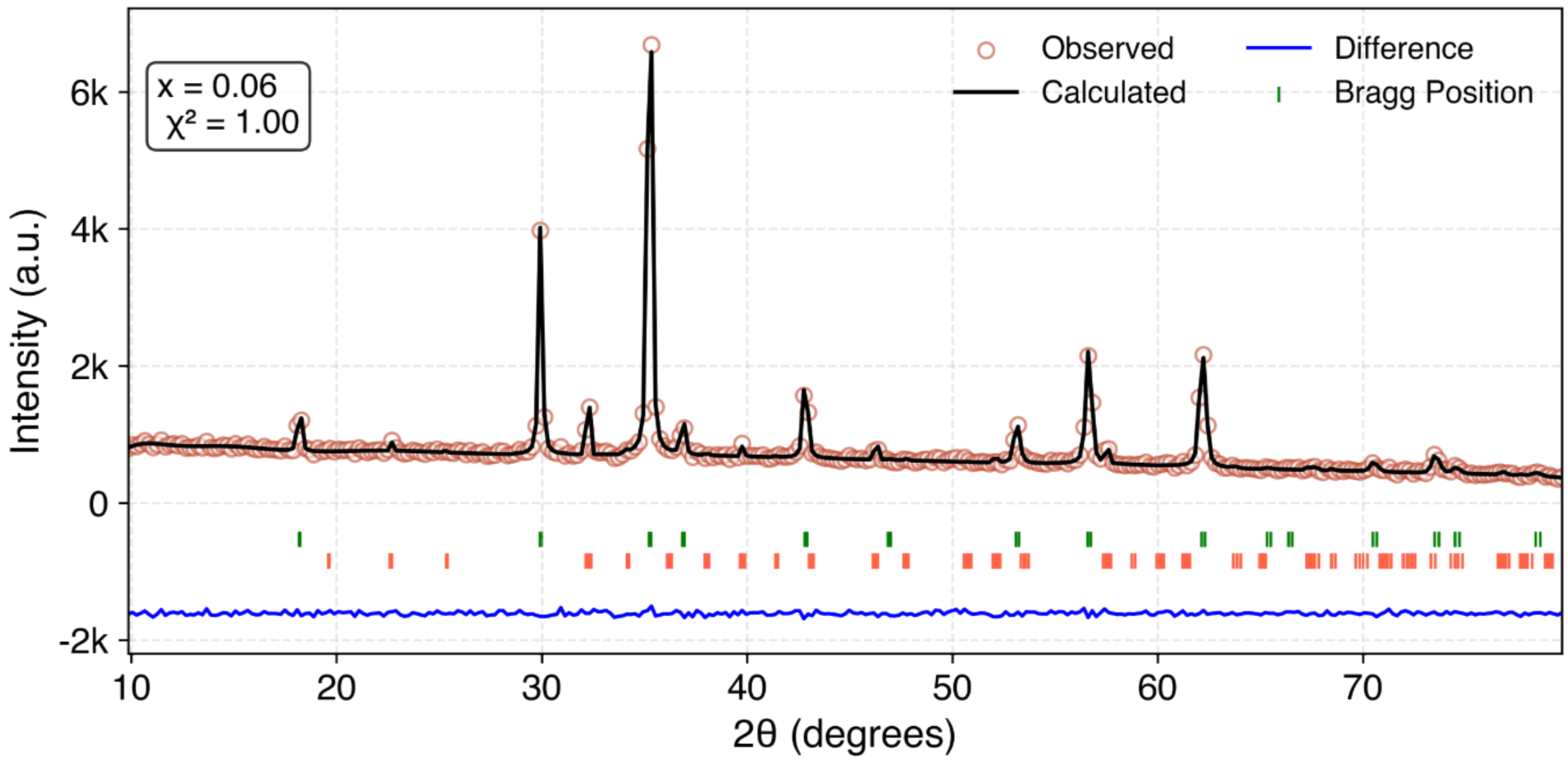

Fig. S2 continued

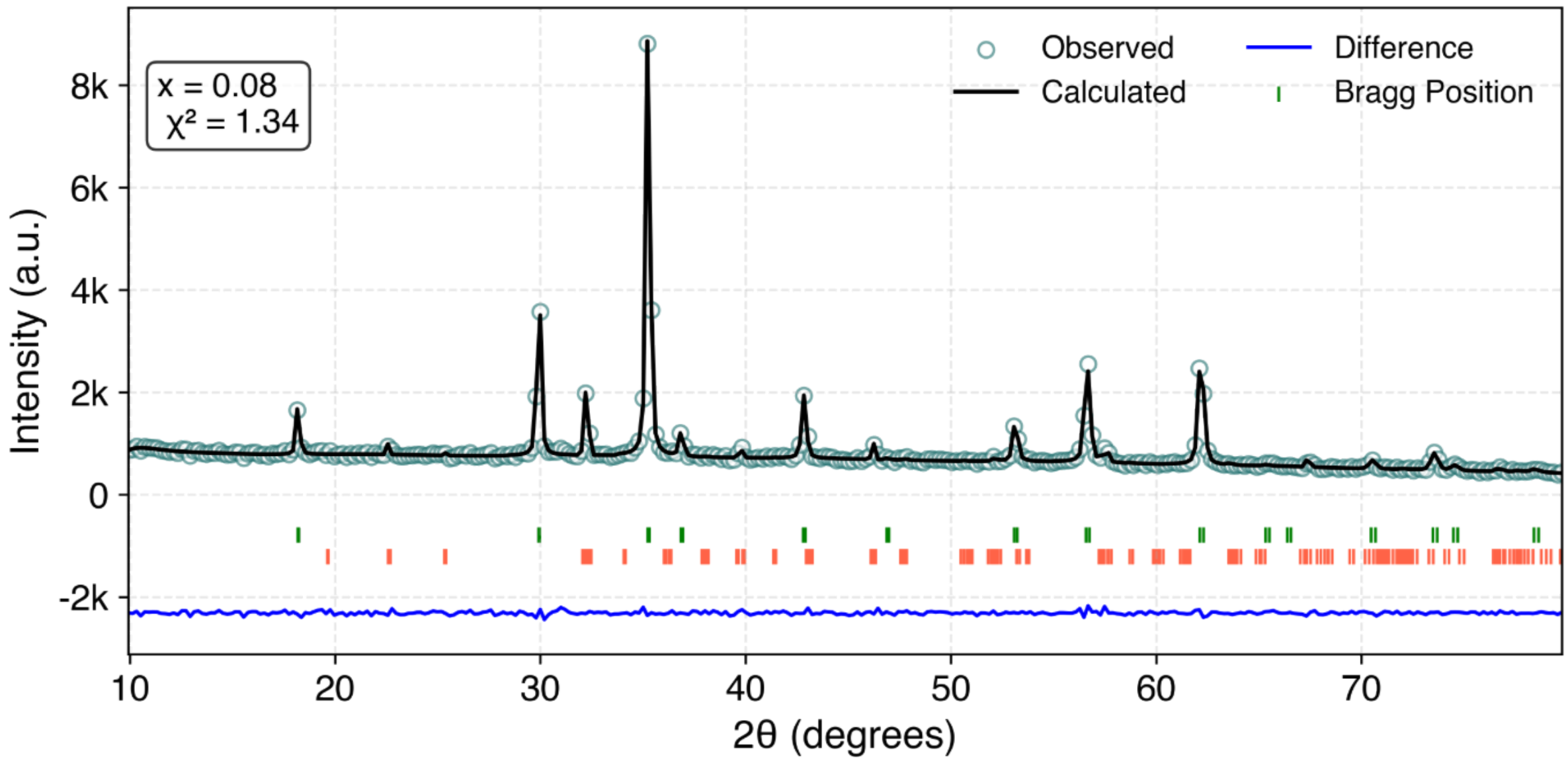

Fig. S2 Individual Rietveld refinement profiles for the MZLF series (x = 0.00 to 0.08). The plots display the experimental observed XRD data (symbols), the calculated FullProf structural model (solid line), the allowed Bragg reflection positions for the primary and secondary phases (vertical tick marks), and the residual difference between the observed and calculated patterns (bottom curve).

3. Nelson-Riley Fitting

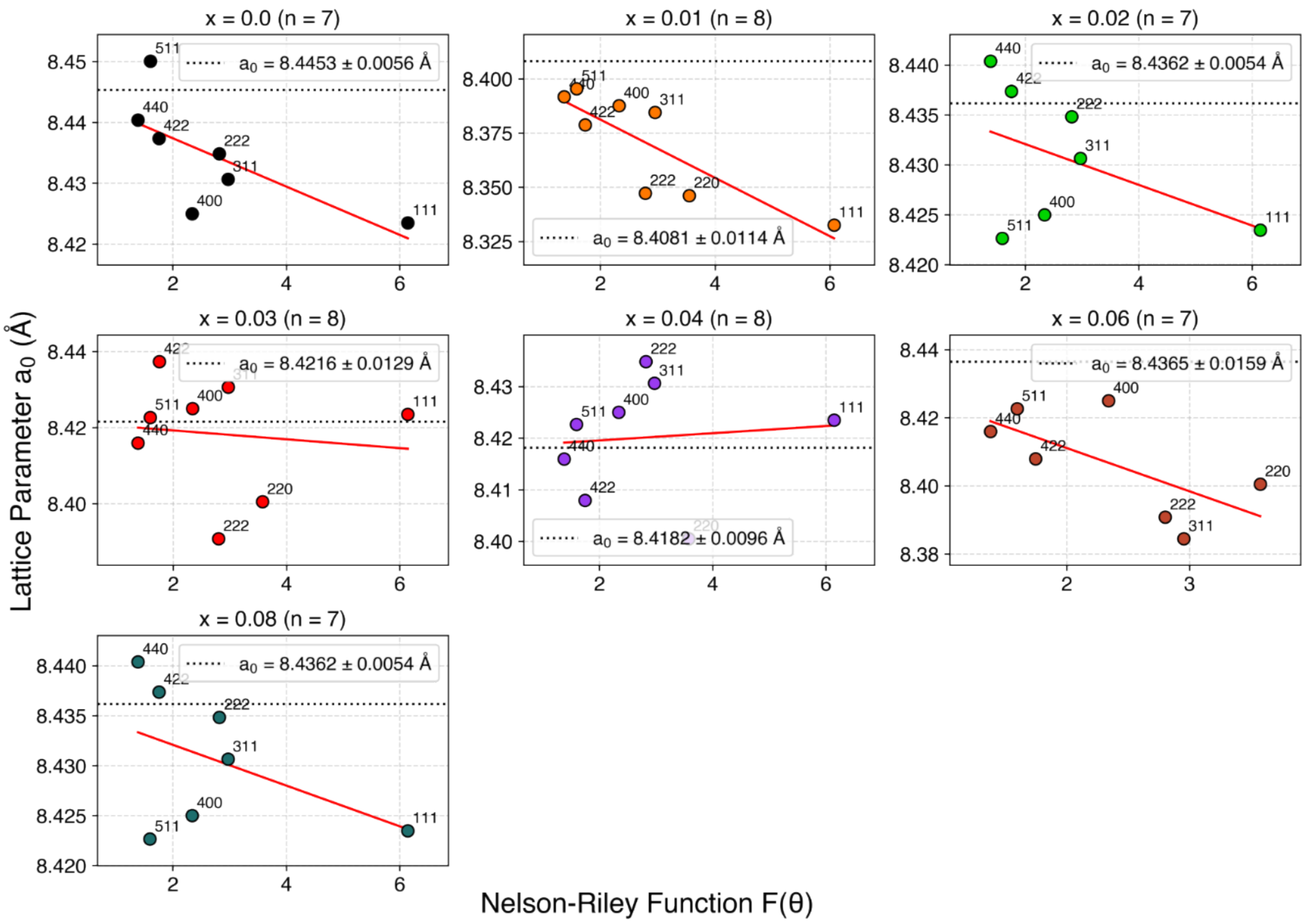

Fig. S3 Nelson-Riley function F(θ) plots for precise lattice parameter a_0 determination of MZLF samples at varying lanthanum concentrations. The solid red lines represent the linear regression fits extrapolated to F(θ) = 0, with labeled data points indicating individual (hkl) Miller indices used for the primary cubic

4. Linear regression analysis for the estimation of crystallite size and microstrain

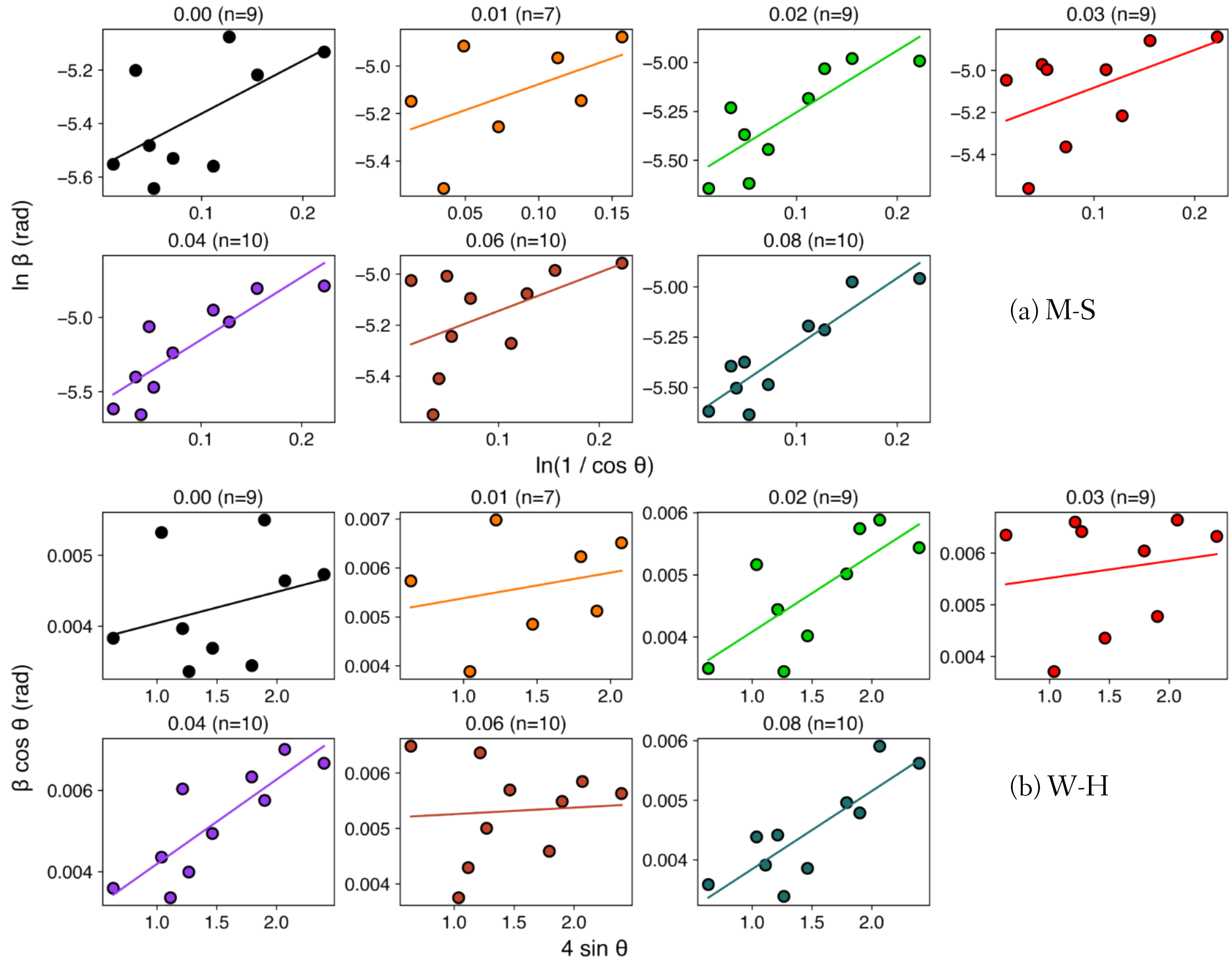

Fig. S4 Linear regression analysis for the estimation of crystallite size and microstrain using **(a)** Modified Scherrer (M-S) plots: Linear fitting of $\ln \beta$ versus $\ln\left(\frac{1}{\cos \theta}\right)$ used to extract the average crystallite size from the y-intercept. The integral breadth β is measured in radians (rad). **(b)** Williamson-Hall (W-H) plots: Linear fitting of $\beta \cos \theta$ versus $4 \sin \theta$, where the slope corresponds to the lattice microstrain and the y-intercept yields the crystallite size. The value of n denotes the total number of distinct diffraction peaks utilized for each fit. Solid lines represent the linear least-squares regression lines applied to the experimental data points.

5. Schematic geometry of the cation-anion-cation exchange pathways

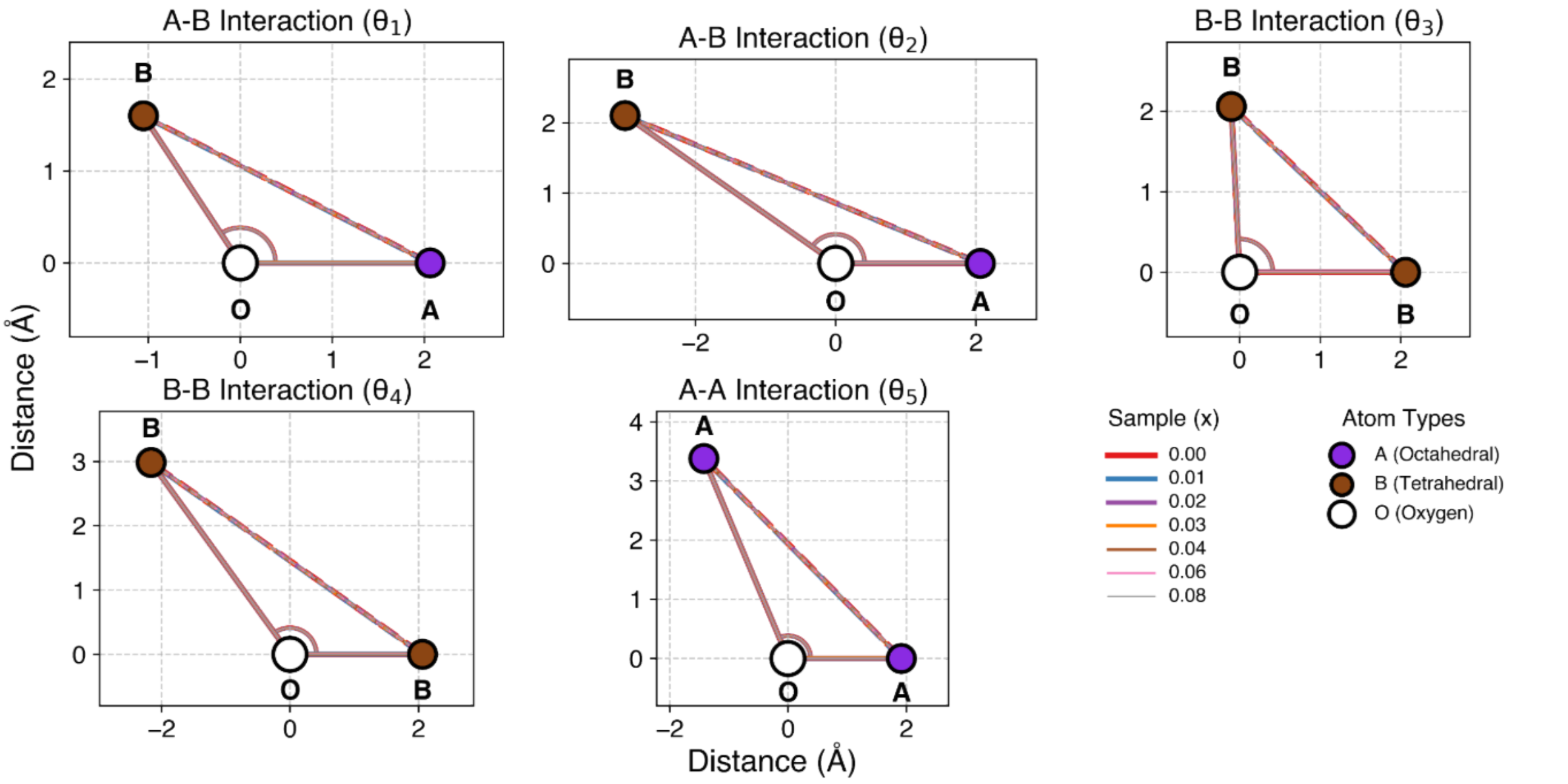

Fig. S5 Configuration of the fundamental structural bonding networks defining the host spinel lattice: A-B interactions (θ_1, θ_2), B-B interactions (θ_3, θ_4), and A-A interaction (θ_5). Both the horizontal and vertical axes quantify interatomic distances in Angstroms (Å). The color-coded profiles (see legend) trace the sub-angstrom variations across different doping concentrations x = 0.00 to 0.08. The structural sites are color-coded to distinguish between the A site (purple circles), B site (brown circles), and oxygen intermediate

6. Digital image processing workflow for microstructural pore network analysis

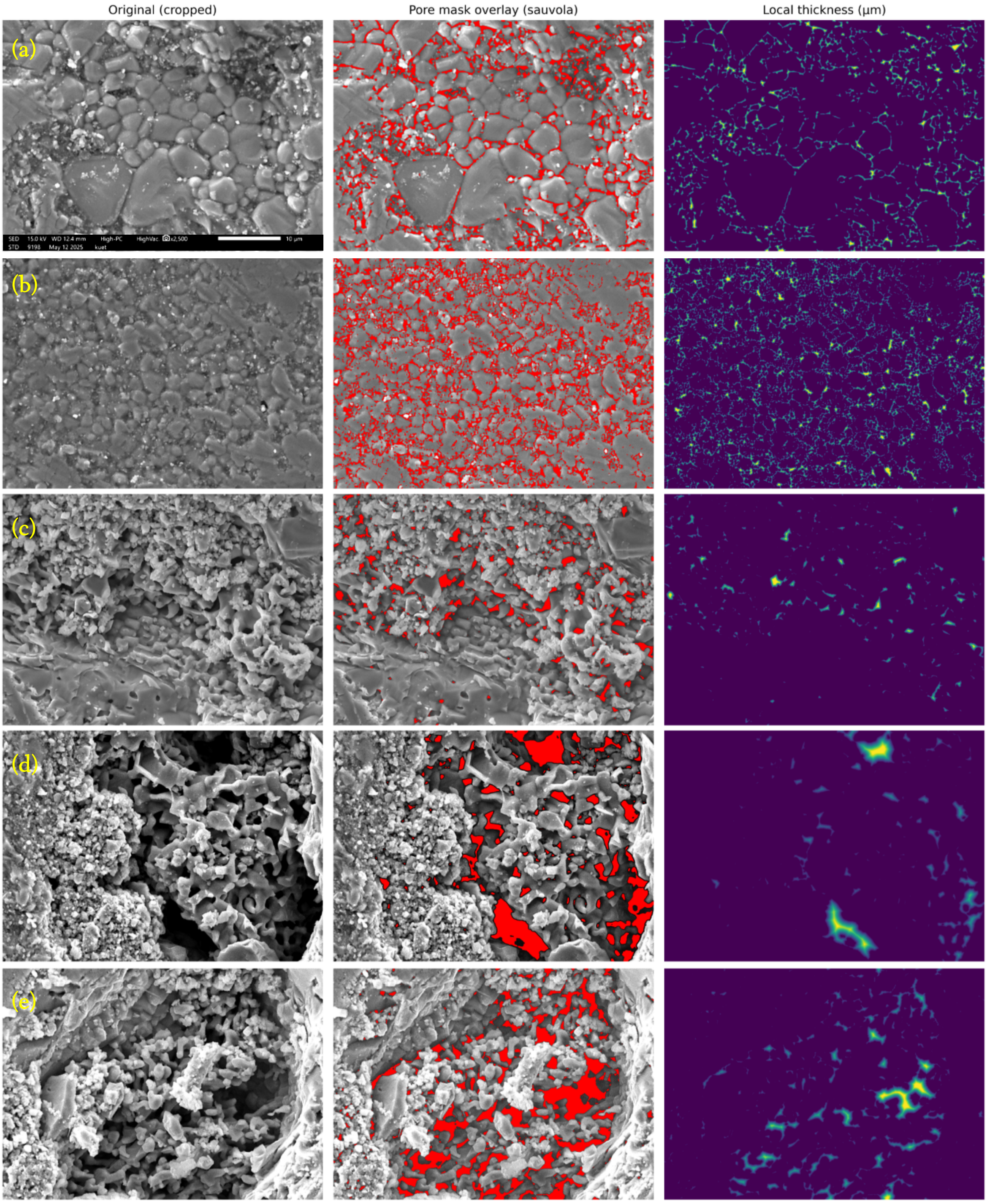

Fig S6 continued

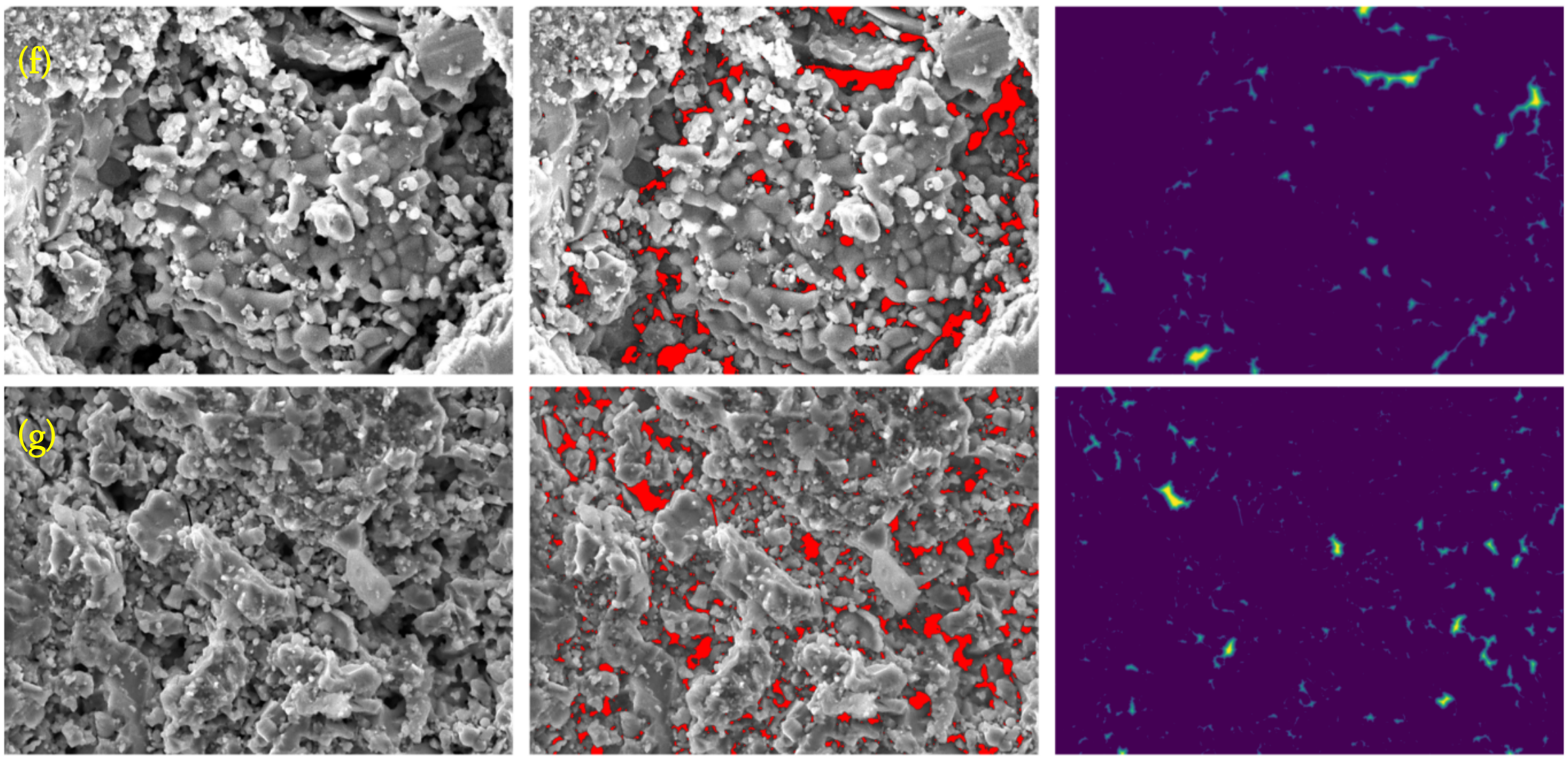

Fig. S6 Quantitative spatial mapping of the specimen porosity across compositions **(a-g)**. Left column: Cropped scanning electron microscopy (SEM) micrographs capturing the raw surface morphology and packing densities. Middle column: Segmented pore network distributions highlighted via a red binary pore mask overlay generated using the different localized adaptive thresholding algorithm. Right column: Pseudocolor heatmaps tracking the local thickness distribution measured in micrometers (μm), where the gradient from purple to yellow defines the varying size and scale of the segmented features.

7. Energy Dispersive X-ray Spectroscopy (EDXS) area-scan mapping regions for the synthesized series

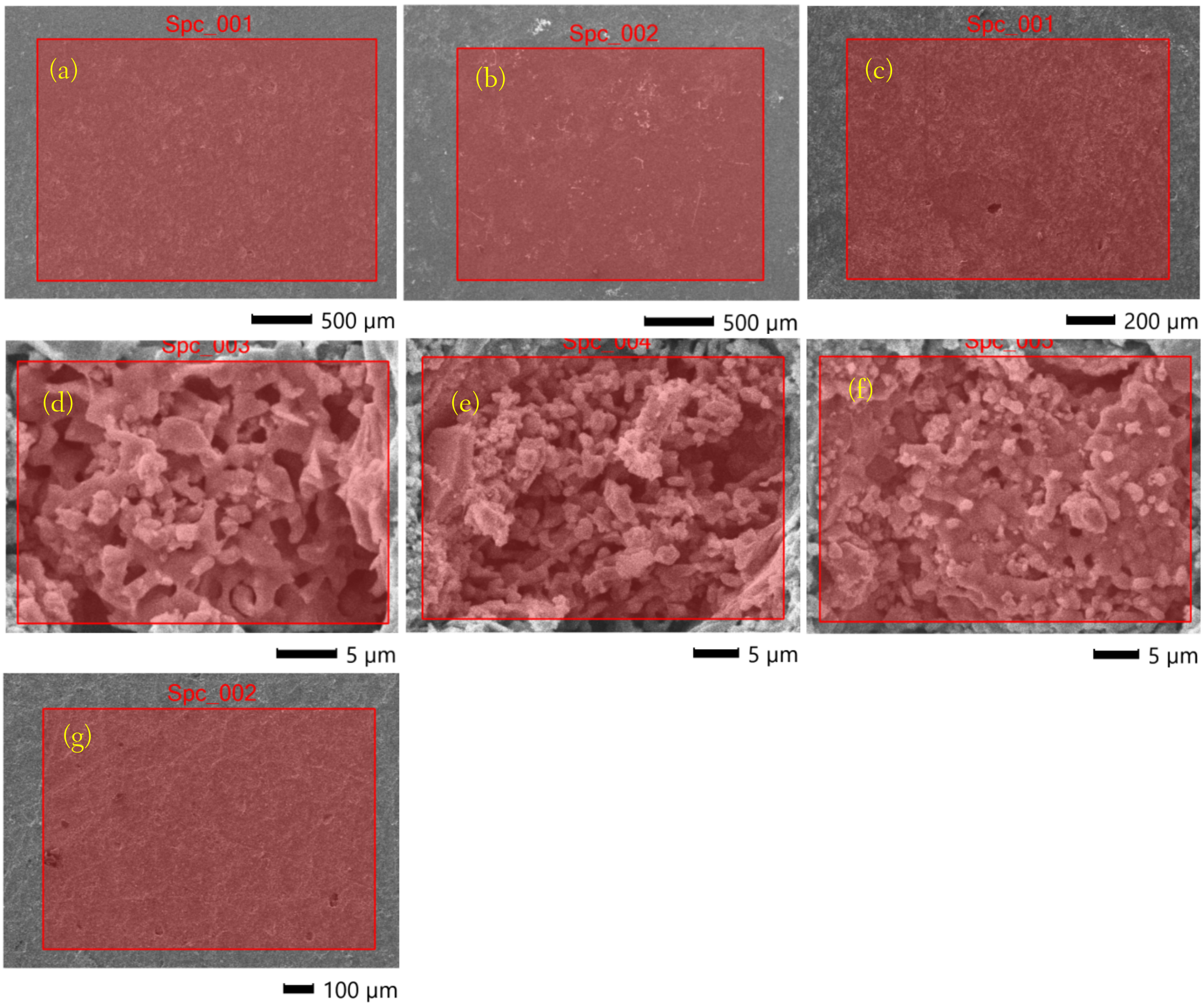

Fig. S7 Scanning Electron Microscopy (SEM) surface micrographs showing the specific red-highlighted rectangular regions utilized for the quantitative collection of Energy Dispersive X-ray Spectroscopy (EDXS) elemental spectra.

8. Compositional dependence of primary magnetic parameters derived from hysteresis loops

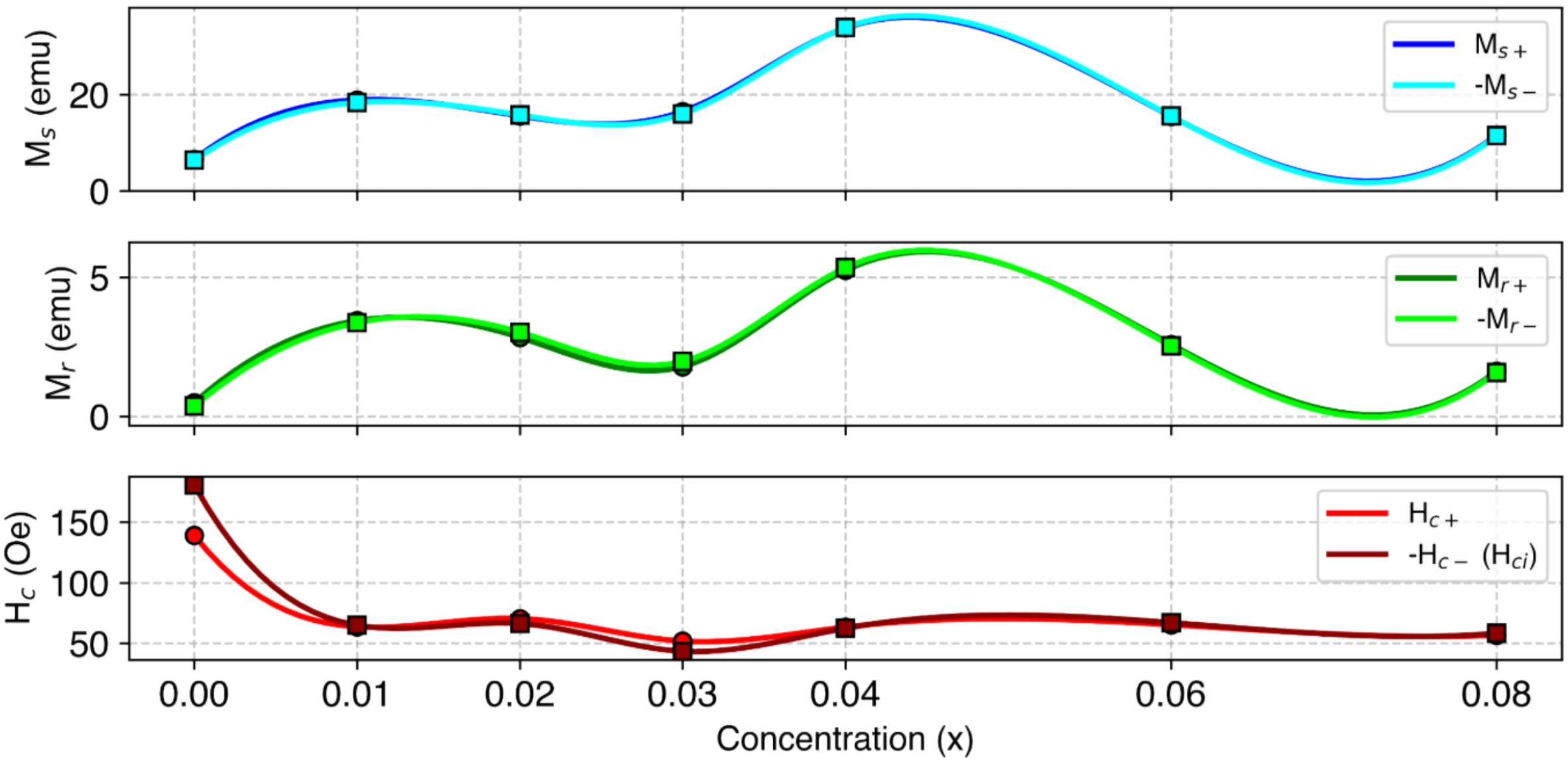

Fig. S8 Quantitative tracking of the magnetic parameter asymmetry across MZLF compositions. Top panel: Variation of positive (M_{s+}, dark blue line) and negative (- M_{s-}, cyan line) saturation magnetization measured in electromagnetic units (emu/g). Middle panel: Variation of positive (M_{r+}, dark green line) and negative (- M_{r-}, light green line) remanent magnetization in emu/g. Bottom panel: Variation of positive coercive field (H_{c+}, red line) and negative coercive field (- H_{c-} or intrinsic coercivity (H_{ci}), maroon line) measured in

9. Differential susceptibility (dM/dH) curves

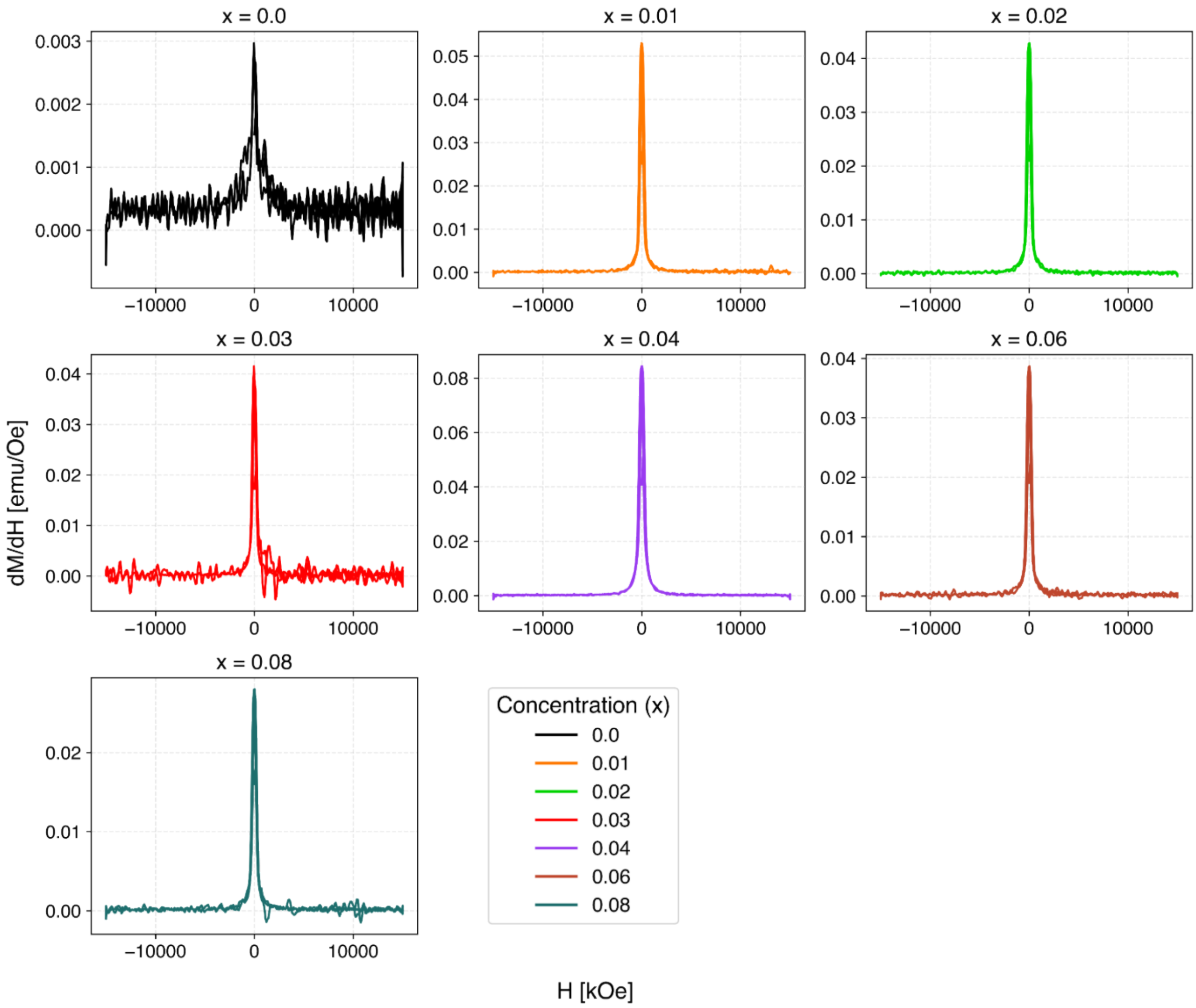

Fig. S9 Differential susceptibility (dM/dH) profiles extracted from Vibrating Sample Magnetometry (VSM) data, plotted in electromagnetic units per Oersted (emu/Oe) against the applied magnetic field (H) in Oersteds (Oe). Each sub-panel represents a specific doping concentration (x), color-coded as shown in the legend. The sharp, highly symmetric peaks centered around zero field reflect the rapid magnetization reversal process, confirming the conventional soft magnetic characteristics of the series.

10. Single Point Detection analysis

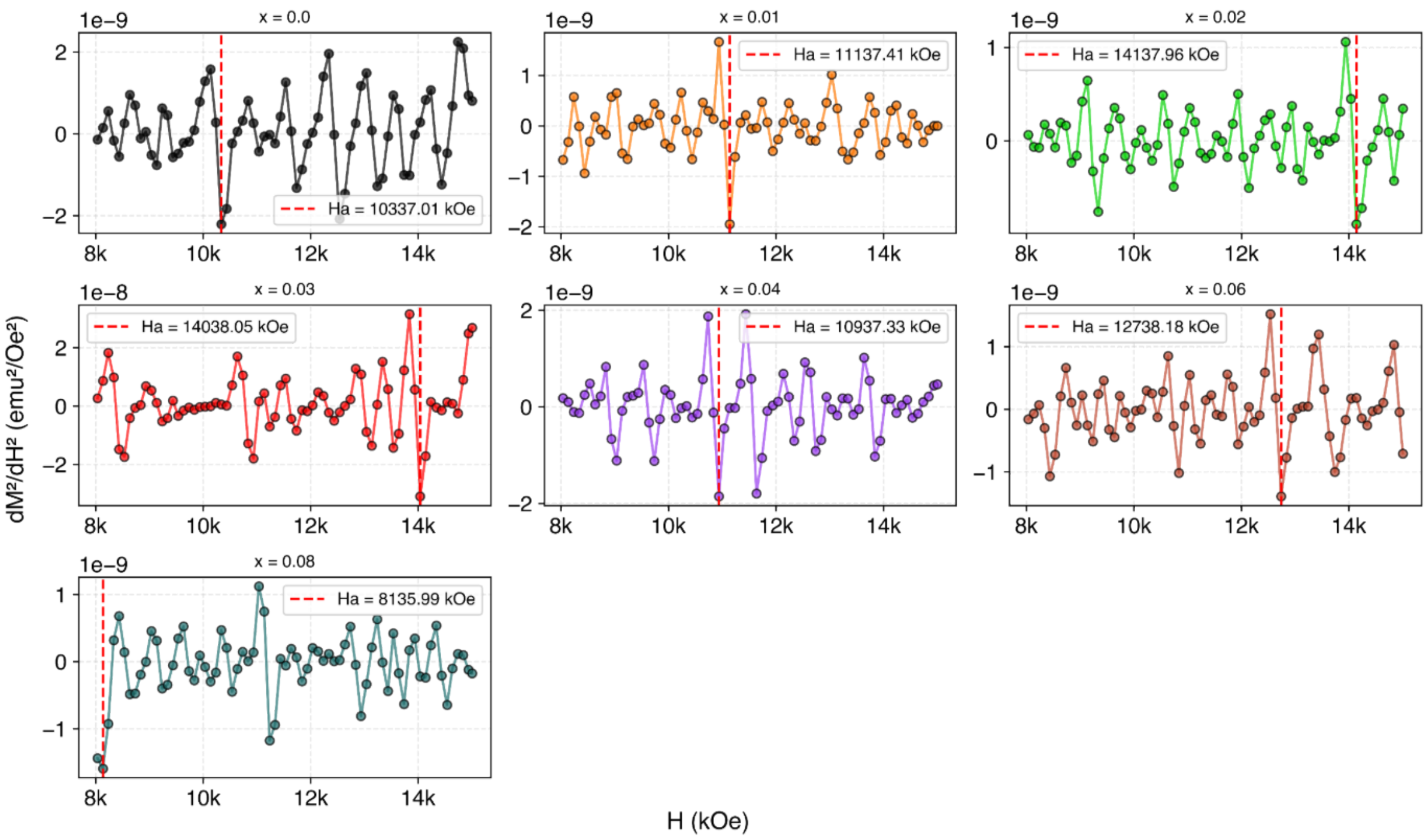

Fig. S10 Single point Detection (SPD) profiles plotted as the second derivative of magnetization ($\frac{dM^2}{dH^2}$) in (emu^2/Oe^2) versus the high applied magnetic field (H) in Oersteds (Oe). The vertical red dashed lines mark the singular minima corresponding to the intrinsic anisotropy field (H_a) for each composition (x). The field scale on the x-axis spans the high-field regime from 8,000 to 15,000 Oe.
